# Evolution and comparative genomics of the most common *Trichoderma* species

**DOI:** 10.1186/s12864-019-5680-7

**Published:** 2019-06-12

**Authors:** Christian P. Kubicek, Andrei S. Steindorff, Komal Chenthamara, Gelsomina Manganiello, Bernard Henrissat, Jian Zhang, Feng Cai, Alexey G. Kopchinskiy, Eva M. Kubicek, Alan Kuo, Riccardo Baroncelli, Sabrina Sarrocco, Eliane Ferreira Noronha, Giovanni Vannacci, Qirong Shen, Igor V. Grigoriev, Irina S. Druzhinina

**Affiliations:** 1 Microbiology and Applied Genomics Group, Research Area Biochemical Technology, Institute of Chemical, Environmental & Bioscience Engineering (ICEBE), TU Wien, Vienna, Austria; 2 Vienna, Austria; 3 Departamento de Biologia Celular, Universidade de Brasília, Brasíla, DF Brazil; 4 US Department of Energy Joint Genome Institute, Walnut Creek, CA USA; 5 Dipartimento di Agraria, Università degli Studi di Napoli „Federico II“, Naples, Portici Italy; 6 CNRS, Aix-Marseille Université, Marseille, France; 7 INRA, Marseille, France; 8 Department of Biological Sciences, King Abdulaziz University, Jeddah, Saudi Arabia; 9 Jiangsu Provincial Key Lab of Organic Solid Waste Utilization, Nanjing Agricultural University, Nanjing, China; 10 Centro Hispano-Luso de Investigaciones Agrarias (CIALE), Departamento de Microbiología y Genética, Universidad de Salamanca, Campus de Villamayor, Calle Del Duero, Villamayor, España; 11 Department of Agriculture, Food and Environment, University of Pisa, Pisa, Italy; 12 Department of Plant and Microbial Biology, University of California Berkeley, Berkeley, CA USA

**Keywords:** Ankyrin domains, CAZymes, Core genome, Environmental opportunism, Gene gain, Gene loss, SSCPs, Orphans

## Abstract

**Background:**

The growing importance of the ubiquitous fungal genus *Trichoderma* (Hypocreales, Ascomycota) requires understanding of its biology and evolution. Many *Trichoderma* species are used as biofertilizers and biofungicides and *T. reesei* is the model organism for industrial production of cellulolytic enzymes. In addition, some highly opportunistic species devastate mushroom farms and can become pathogens of humans. A comparative analysis of the first three whole genomes revealed mycoparasitism as the innate feature of *Trichoderma*. However, the evolution of these traits is not yet understood.

**Results:**

We selected 12 most commonly occurring *Trichoderma* species and studied the evolution of their genome sequences. *Trichoderma* evolved in the time of the Cretaceous-Palaeogene extinction event 66 (±15) mya, but the formation of extant sections (*Longibrachiatum, Trichoderma*) or clades (*Harzianum/Virens*) happened in Oligocene. The evolution of the *Harzianum* clade and section *Trichoderma* was accompanied by significant gene gain, but the ancestor of section *Longibrachiatum* experienced rapid gene loss. The highest number of genes gained encoded ankyrins, HET domain proteins and transcription factors. We also identified the *Trichoderma* core genome, completely curated its annotation, investigated several gene families in detail and compared the results to those of other fungi. Eighty percent of those genes for which a function could be predicted were also found in other fungi, but only 67% of those without a predictable function.

**Conclusions:**

Our study presents a time scaled pattern of genome evolution in 12 *Trichoderma* species from three phylogenetically distant clades/sections and a comprehensive analysis of their genes*.* The data offer insights in the evolution of a mycoparasite towards a generalist.

**Electronic supplementary material:**

The online version of this article (10.1186/s12864-019-5680-7) contains supplementary material, which is available to authorized users.

## Background

The Sordariomycetes, one of the largest classes in the Division Ascomycota, display a wide range of nutritional strategies including saprotrophy and biotrophic interactions with bacteria, plants, animals, fungi or other organisms [1]. Within them, the highest number of known genera is found in the order Hypocreales [2] that comprises half of the whole-genome sequenced species of Sordariomycetes (Nov. 2017, NCBI Taxonomy Browser). Molecular data suggest that the ancestors of the Hypocreales evolved some 170–200 Mya as fungi associated with plants either as parasites or saprotrophs [3]. The diversification into extant taxa was accompanied by several intra- and interkingdom host shifts involving fungi, higher plants, and animals [4]. Among them, parasites of animals likely appeared first in the Jurassic period, and specialized entomoparasitic families developed during the Cretaceous period, thereby following the diversification of herbivory insects and angiosperms [3].

Mycoparasitic fungi can be found in species from several fungal taxa [5], but only the Hypocreales contain exclusively fungicolous genera, i.e. *Hypomyces, Escovopsis,* and *Trichoderma*. The ancestor of these mycoparasitic fungi likely evolved at the same time as the entomoparasites, but the time and events of *Trichoderma* evolution are not known.

Among these fungicolous fungal genera, *Trichoderma* is the largest taxon with many ubiquitously distributed species. Detailed ecological and biogeographic surveys of *Trichoderma* [6–9] revealed that species of this genus are most frequently found on the fruiting bodies of other fungi and the dead wood colonized by them. While mycoparasitism in *Hypomyces* is frequently species-specific and restricted to fruiting body forming Basidiomycota [10], the genus *Trichoderma* is unique as many of its species can parasite also on Ascomycetes and even on phylogenetically close species [11].

**Fig. 1:**
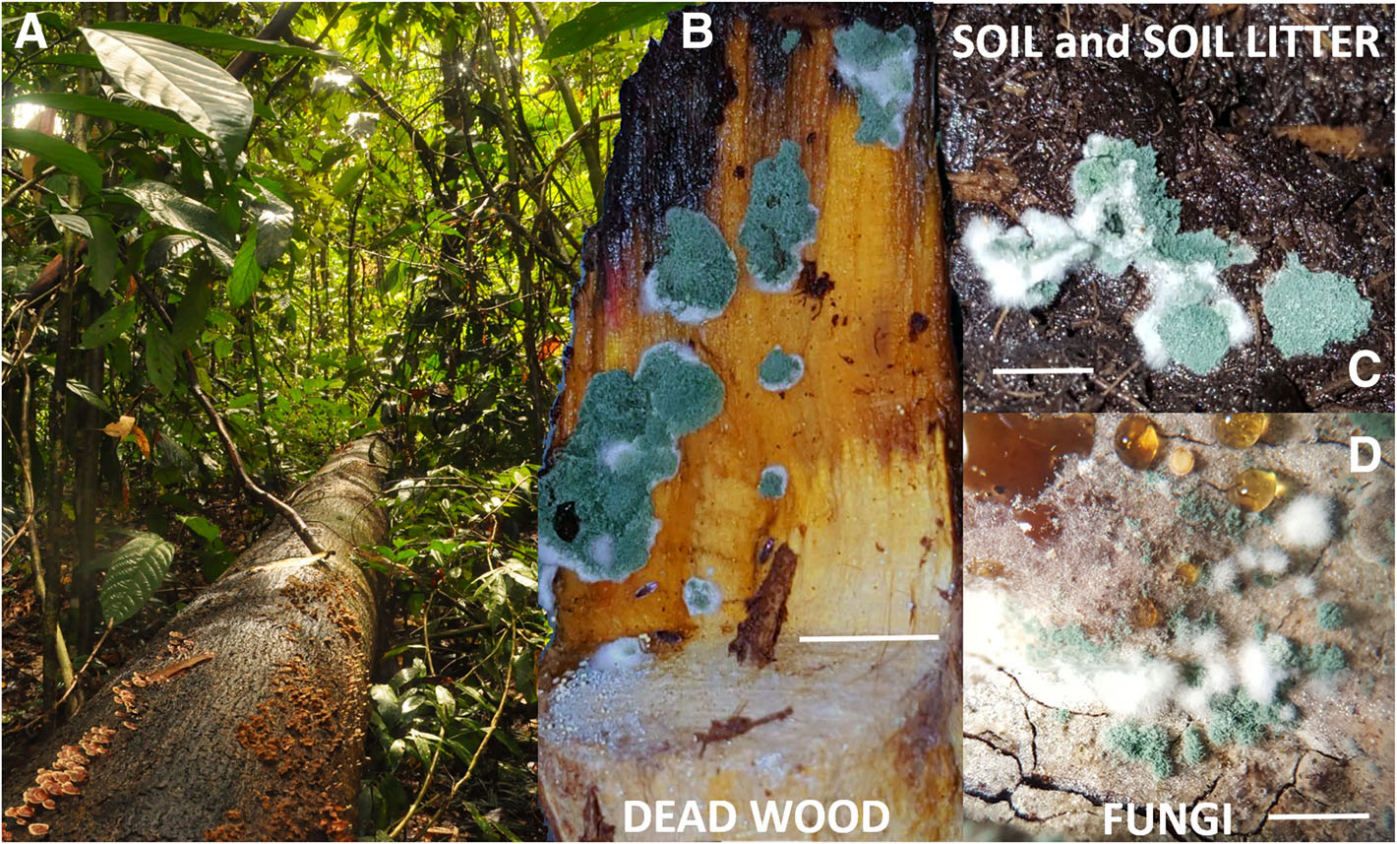
*Trichoderma* spp. in nature. **a** The fallen log of the dead wood colonized by the other fungi represents the major ecological niche for *Trichoderma* spp. **b** *Trichoderma atroviride* on dead wood. **c** *T. harzianum* on soil. **d** *T. simonsii* on the sporocarps of *Stereum* sp. Some species may also colonize soil and become endophytes. Scale bar on B and C corresponds to 1 cm

An analysis of the genomes from three species of *Trichoderma* (*T. reesei, T. virens, T. atroviride*) suggested that mycoparasitism as an innate property of *Trichoderma* [12], but these species are also characterized by considerable nutritional versatility [13]: in addition to acting as mycoparasite, which promoted its use as a biocontrol agent against plant pathogenic fungi [12, 14], *Trichoderma* has become an opportunistic infectant of humans [12]. To date, *Trichoderma* is rarely reported as a parasite on plants and invertebrates but it can colonize plants as a symptomless endosymbiont [12]. Finally, many species of the genus grow efficiently on dead plant biomass and one of its species - *T. reesei* - is a major industrial source of cellulases and hemicellulases. Interestingly, the most opportunistic *Trichoderma* species may also grow in soil where they can either establish in a bulk soil or colonize rhizosphere. As plants usually positively respond to the presence of *Trichoderma*, this property attracts raising attention for the use of these fungi in *bio*fertilizers. The fact that some *Trichoderma* species can feed on plant, fungal and animal bodies characterizes them as generalists. (Fig. 1).

It is not known how generalism evolved from the phytosaprotrophic background of the Hypocreales*.* Chaverri and Samuels [15] compared a phylogenetic tree of the genus *Trichoderma* with the habitats from which the individual species had been isolated and concluded that the evolution of the genus involved several interkingdom host jumps and that preference for a special habitat was gained or lost multiple time. It has been argued that the versatility of *Trichoderma*’s nutritional strategies can be described by the expansions of the spectrum of hosts and substrates due to enrichment of its genome by the laterally transferrered genes required for the feeding on the plant biomass [11].

The hypothesis of this work was that a comparative genomics of those species of *Trichoderma* which are most frequently sampled (and therefore must be most successful generalists) and an analysis of their pattern of gene evolution would reveal the evolutionary events that shaped the nutritional expansions and environmental generalism. In addition, identification of the gene inventory of the *Trichoderma* core genome (i.e. the genes that are present in all species) and its intersection with genomes of other fungi would reveal the specific genomic features of these industrially-relevant fungi.

Although the sequences of several *Trichoderma* genomes have already been published [11, 16–24], detailed genome wide analyses have been published for only three of them (*T. reesei, T. virens* and *T. atroviride* [11, 16, 25–27]). To test the hypothesis raised above, we have analysed the evolution and the gene inventory of the genomes from 13 *Trichoderma* isolates that represent 12 species with a worldwide distribution and are members of three major infrageneric groups [7].

## Results

### Selection of the most common *Trichoderma* species

To reveal the most frequently sampled species in the genus *Trichoderma*, we have first calculated the number of nucleotide sequences deposited for *Trichoderma* spp. in NCBI GenBank (see Methods). There is today general agreement that the new *Trichoderma* spp. can only be defined by at least three or more gDNA sequences while the analysis of usually two DNA barcode fragments is required for the species identification [7–9]. The number of gene sequences in NCBI per each species may therefore roughly correspond to the number of isolates detected for this species and thus approximate the frequency of the general species occurrence. This analysis revealed (Additional file 1) that most species (80%) were relatively rare as they were represented by < 50 gene sequences each, whereas 35 species (12% of the total number of species) were represented by more than 100 nucleotide sequences each. Of these, 84% of nucleotide sequences were attributed to a small group of common species: *T. harzianum* sensu lato (also deposited as *T. lixii* or *Hypocrea lixii*) was responsible for 32% (9532 sequences) of total sequences. This was followed by *T. asperellum, T. atroviride, T. longibrachiatum, T. gamsii,* and *T. virens* that were represented by > 1000 sequences each and therefore also frequent.

**Fig. 2:**
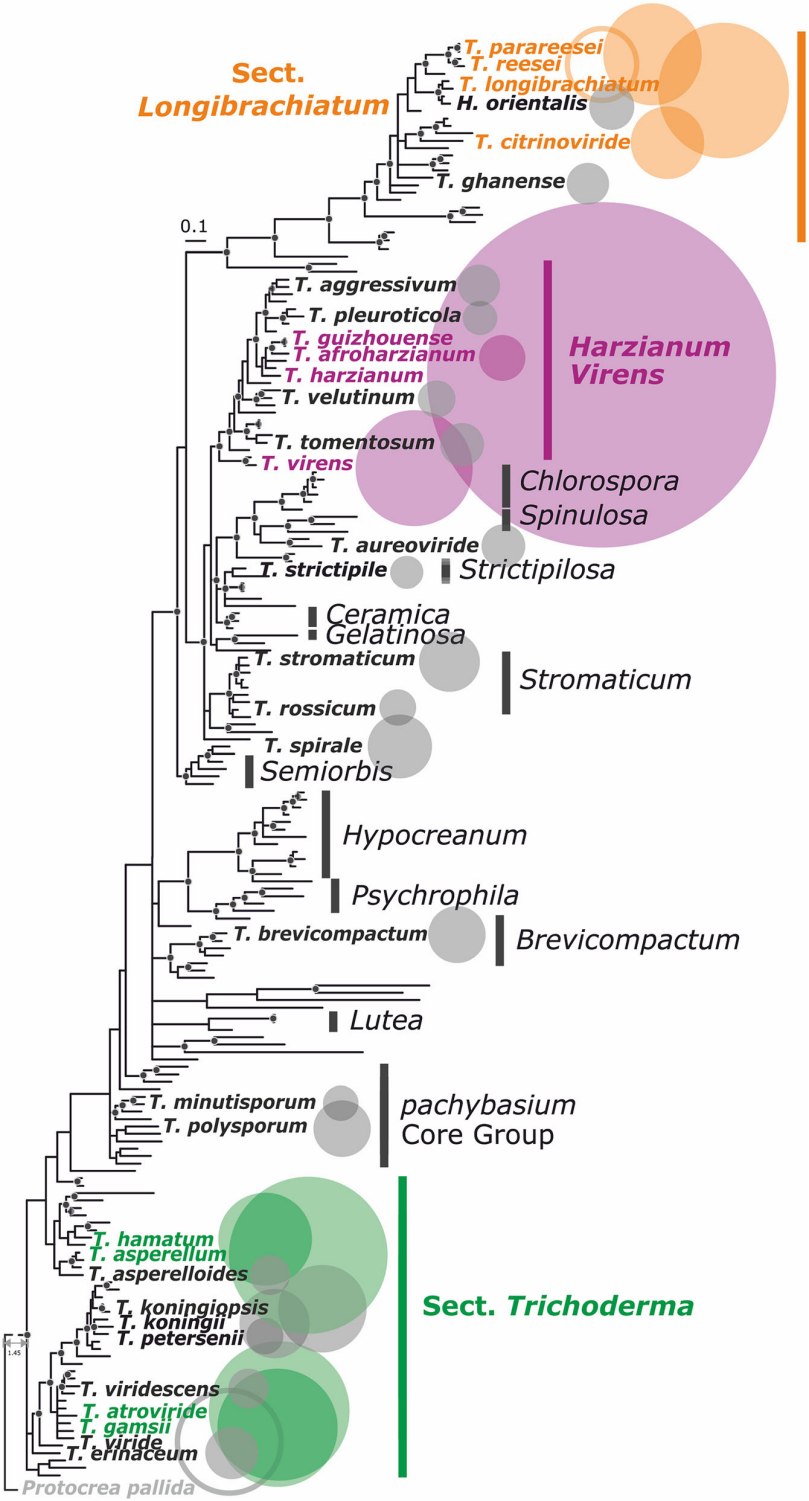
Phylogeny of the genus *Trichoderma* and occurrence of the most common species. Phylogeny of *Trichoderma* based on Bayesian analysis of the *rpb2* gene (see Methods for details). Only species with major abundance (> 100 nucleotide sequences deposited in NCBI GenBank, April 2018,) are shown. The number of core nucleotide sequences deposited in GeneBank is indicated by the size of the filled circles with *T. pleuroticola*, *N* = 103 being the smalles shown. Sections *Longibrachiatum* and *Trichoderma* and the *Harzianum/Virens* clade are indicated by colored vertical bars. Rare *Trichoderma* spp. (< 100 nucleotide sequences known in public databases) are not shown. Circles for *T. reesei* and *T. viride* likely represent false positive values as *T. reesei* is most studied species, while *T. viride* is the oldest *Trichoderma* species name that was assigned to all strains before DNA barcoding became available

The mapping of the nucleotide sequence abundance (Additional file 1) on the phylogenetic tree of the genus consisting of > 200 species showed that the most frequent species are not present in one or a few infraneric groups but are distributed among different clades (Fig. 2). The most frequent and putatively environmentally successful species are found in the *Harzianum*/*Virens* clades (HV), section *Longibrachiatum* (SL) and section *Trichoderma* (ST). As all of the common species are profound generalists with cosmopolitan distribution, this property could either be an evolutionary old adaptation lost by other (rare) *Trichoderma* species or the nutritional versatility independenty evolved in each phylogenetic group of the genus.

### General comparison of the genomes of twelve *Trichoderma* species

Based on the above analysis, we compared the genomes from 12 *Trichoderma* species: *T. reesei, T. longibrachiatum, T. citrinoviride, T. parareesei* from section *Longibrachiatum* (SL), *T. harzianum* (the the ex-type strain CBS226.95 marked with “^T^” throughout the manuscript, and strain TR274)*, T. guizouense, T. afroharzianum, T. virens* from *Harzianum* and *Virens* clades (HV), and *T. atroviride, T. gamsii, T. asperellum,* and *T. hamatum* from section *Trichoderma* (ST). The relation between the species is shown on Fig. 2. The species concept of *T. harzianum* has recently been revised [28] and it is not known what percentage of the newly defined species would account for “*T. harzianum*” entries in GeneBank. We therefore included *T. guizhouense* and *T. afroharzianum*, two species with worldwide distribution [28, 29], and two strains of *T. harzianum* (one from Northern Europe and one from Brazil [30]) in this study. *T. parareesei* and *T. gamsii* were included because they are sibling species of *T. reesei* and *T. atroviride*, respectively (Fig. 2).

As already mentioned, the genome sequencing for some of these species has been reported before. For the sequencing of the new strains and annotation improvement of previously published genomes of *T. reesei* and *T. hamatum* see Methods and Additional files 2, 3, and 4. The *Trichoderma* genomes vary in size (33 - 41 Mb), species from SL having the smallest ones (Table 1). Consequently, the number of predicted genes in *Trichoderma* varies between 9292 and 14,095, which is in the range of that in other Sordariomycetes genomes (https://genome.jgi.doe.gov/fungi/fungi.info.html) [31]). In correlation with the genome size, species from SL also contain the smallest gene inventory (9292–10,938 genes). As shown in Table 1, most of the genomes were 94 to 97% complete as predicted by BUSCO [32], only *T. longibrachiatum* displaying a lower value (86%). The absence of a gene in the latter species was therefore treated with caution if it was found in all other species from SL.

**Table 1:** Properties of the *Trichoderma* genomes and gene distribution

Clade	Species	Strain	Genome size (Mbp)	Total genes	Complete-ness (%)	Fragmen-ted (%)	Missing (%)	Orthologs and paralogs
*Longibrachiatum*	*T. reesei* ^*a*^	QM6a	32.7	9877	96.9	2.4	0.7	8090
		RUT C30	34.2	10877				
	*T. longibrachiatum*	ATCC18648	31.74	10938	86.3	7.9	5.8	8229
	*T. citrinoviride*	TUCIM 6016	33.2	9737	94.1	3.1	2.8	7834
	*T. parareesei*	CBS125925	32.07	9292	95.3	3.7	1	8328
*Harzianum/Virens* Clades	*T. harzianum*	CBS 226.95	40.9	14095	98.1	1.4	0.5	9921
		TR257	39.4	13932	97.2	2	0.8	9870
	*T. afroharzianum*	T6776	39.7	11297	95.1	1.8	3.1	9541
	*T. guizhouense*	NJAU4742	38.8	11297	98.3	1.2	0.5	9246
	*T. virens*	Gv29–8	40.52	12427	97.8	1.9	0.3	9795
*Trichoderma*	*T. atroviride*	IMI 206040	36.4	11863	97.5	2.1	0.4	9301
	*T. gamsii*	T6085	37.9	10709	94.1	2.1	3.8	8825
	*T. asperellum*	CBS433.97	37.66	12586	97.9	1.4	0.7	9143
	*T. hamatum*	GD12	38.43	10520	98.6	0.7	0.7	9030

^a^ numbers show data (from left to right) obtained in this paper and by Li et al. [27] (if available)

The average protein sequence similarity within *Trichoderma* orthologues ranges from 90% (SL vs ST) and 92.5% (SL vs HV) to 97–99% within species from the same sections/clades (Table 2). Amino acid similarity with other Hypocreales was still high (75–78.4%), but considerably lower with *Neurospora crassa* and *Chaetomium globosum* (58–60%) (Additional file 5).

**Table 2:**
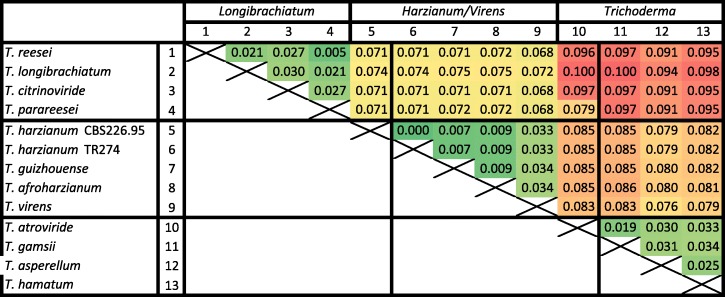
Pairwise genetic distance between orthologous proteins from 13 *Trichoderma* strains^a^

^a^colors show relative high (red), intemediate (yelow) and low (green) values

### Evolution of the twelve *Trichoderma* species

**Fig. 3:**
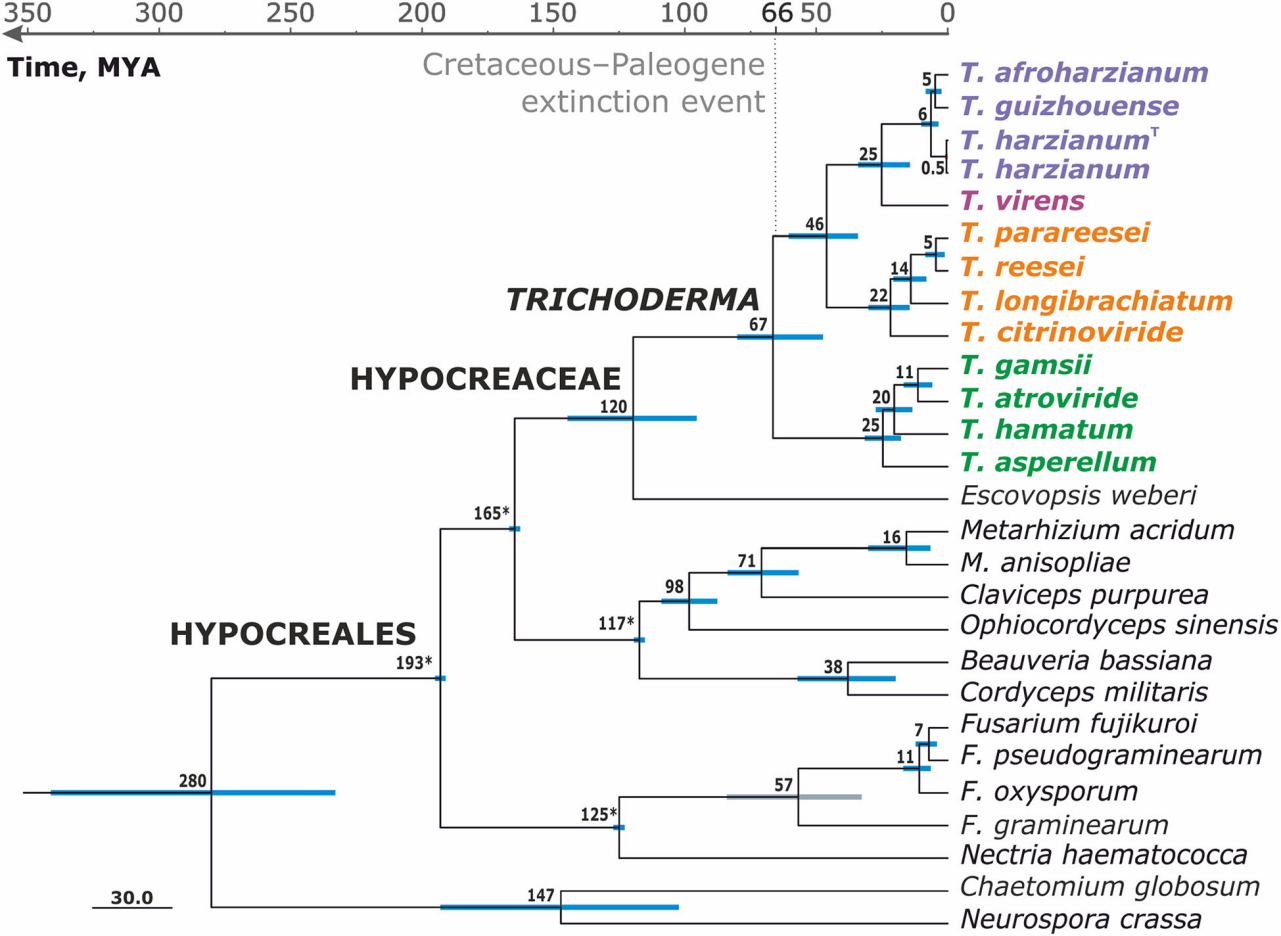
Bayesian chronogram obtained based on the concatenated alignment of 638 core orthologous proteins of Hypocreales and the two other Sordariomycetes. All nodes were supported with PP = 1. Chronological estimations are given in a geological time scale in Mya, and the numbers represent the corresponding node age. Numbers with asterisks at nodes indicate calibration points against the origin of Hypocreales (see Methods for details). Bars correspond to 95% confidence interval in time estimation based on the lognormal relaxed clock

To learn the evolution of *Trichoderma,* we subjected the 13 strains (twelve species), twelve other fungi of the Hypocreales, and two phylogenetically more distant Sordariomycetes - *N. crassa, C. globosum* (outgroup) - to a time-scaled phylogenetic analysis using 638 orthogous genes (see Methods). The resulting tree (Fig. 3) shows that *Trichoderma* evolved 66.5 (± 15) mya, next to the Cretaceous-Paleogene (K-Pg) extinction event characterized by massive extinction of plants and large animals [33]. The two sections (SL, ST) and the HV clade of extant *Trichoderma* species appear to have arisen 25–21 mya what corresponds to the late Paleogene/early Miocene. The tree thereby confirms the ancient nature of section ST [12], and documents that SL and HV are monophyletic and evolved later. The divergency between the two strains of *T. harzianum* (i.e. one from Europe, UK, and one from South America, Brazil) was calculated to have occurred 460,000 years ago. Speciation of *T. afroharzianum* and *T. guihouense* can be dated around 5–6 Mya, which is comparable to that between *T. reesei* and *T. parareesei,* what justifies their recognition as separate species [28].

### *Trichoderma* gene inventory

To analyze and compare the gene inventory of *Trichoderma*, all putative proteins from the thirteen *Trichoderma* genomes and the fourteen other Sordariomycete species shown in Fig. 3 were analysed by the Markov Cluster Algorithm (MCL). We detected 19,332 clusters (Additional file 6), of which 7923 clusters contained genes from at least two *Trichoderma* species from each section. Further, 2095 clusters were shared by species from one or two sections/clades. HV exhibited the highest number of unique genes and also shared the highest number of genes with ST (Table 3). No PFAM domain could be assessed to 1980 of the above 19,332 clusters of which 1485 were present in single copies in each strain (or absent from only one species) and therefore putative orthologs.

**Table 3:** Distribution of *Trichoderma* genes in sections, clades and species

Clade	Species	Present in			Absent from			Total:
		All clusters with at least one gene from *Trichoderma*	At least two species from each clade	the clade only	SL	HV	ST	
		13,089	7923	80/745/286	1083	68	335	
SL	*T. reesei*	8775	8176	80		38	232	8526
	*T. longibrachiatum*	8636	7951	80		46	249	8326
	*T. citrinoviride*	9205	8436	81		48	275	8840
	*T. parareesei*	8757	8105	81		37	234	8457
HV	*T. harzianum* ^*T*^	12737	9419	778	1038		300	11535
	*T. harzianum*	12698	9392	763	1016		296	11467
	*T. guizhouense*	10996	9036	533	826		230	10625
	*T. afroharzianum*	10811	8802	500	867		232	10401
	*T. virens*	11474	9341	410	875		276	10902
ST	*T. atroviride*	10738	9009	267	841	54		10171
	*T. gamsii*	10039	8501	267	780	31		9579
	*T. asperellum*	10595	8886	266	839	47		10038
	*T. hamatum*	10164	8694	241	833	29		9797

MCL clusters with the highest number of genes (> 2000 in all 13 *Trichoderma* strains) comprised those encoding the fungal-specific Zn_2_Cys_6_ transcriptional regulators, solute transporters of the major facilitator superfamily (MSF) and short-chain dehydrogenases/reductases (SCDR) (Table 4). In addition, the 13 *Trichoderma* genomes had more than thousand genes that encoded proteins with ankyrin repeats, alpha/beta type hydrolases, protein kinases, zinc-dependent alcohol dehydrogenases, FAD-binding oxidases, methyltransferases and AAA + -ATPases. With respect to clade-specific distribution, the highest number of genes for individual protein families were present in species of HV and ST and were in 1.5–2-fold excess over those in SL. Proteins with a NmrA domain (regulators of GATA-type transcriptional regulators; [34]) were present in HV and ST even in threefold numbers (Table 4). Eighty percent of the genes encoding ankyrins, heteroincompatibility (HET) proteins, zinc-dependent alcohol dehydrogenases, cytochrome P450 monooxygenases and NmrA-like proteins were present in multigene clusters, suggesting that their evolution involved gene duplications. On the other hand, gene clusters encoding protein kinases, AAA + -ATPases, amino acid transporters, DEAE-box helicases or proteins wth an RMM_1 (ribonucleotide reductase M1) domain occurred in the same number in all species (Table 4).

**Table 4:** PFAM group members with more than 500 genes in the 13 *Trichoderma* isolates

		clusters	genes per cluster	total genes	genes/ species	HV/SL^a^	HV/ST^a^	ST/SL^a^	singletons^b^	multiples^c^	C/S^d^ (%)
Zn2Cys6 transcriptional regulators	PF04082	238	12.3	2929	225.3	1.69	1.21	1.40	84	154	64.7
MFS permeases	PF07690	172	17.3	2972	228.6	1.55	1.06	1.46	67	105	61.0
short-chain dehydrogenases/reductases	PF00106	121	17.6	2129	163.8	1.68	1.18	1.42	46	75	62.0
ankyrin-containing proteins	PF00023	106	14.4	1524	117.2	1.84	1.05	1.75	20	86	81.1
alpha-beta-hydrolases	PF00561, 07859, 02230	96	14.4	1382	106.3	2.02	1.27	1.59	29	67	69.8
protein kinases	PF00069	98	12.4	1218	93.7	1.15	1.11	1.03	60	38	38.8
zinc-dependent alcohol dehydrogenases	PF00107	66	17.6	1159	89.2	1.89	1.17	1.61	15	51	77.3
FAD-binding oxidases	PF01494, 01565	84	13.8	1158	89.1	1.56	1.21	1.29	25	59	70.2
methyltransferases	PF00891	91	12.7	1157	89.0	1.24	1.09	1.14	46	45	49.5
AAA + -ATPasesAAA+ − ATPases	PF00004	85	13.3	1130	86.9	1.13	1.06	1.07	54	31	36.5
cytochrome P450 monooxygenases	PF00067	59	15.9	940	72.3	1.65	1.69	0.97	14	45	76.3
sugar transporters	PF00083	65	14.2	923	71.0	1.53	1.17	1.31	33	32	49.2
ABC-transporters	PF00005	48	16.3	780	60.0	1.22	1.13	1.07	21	27	56.3
vegetative heteroincompatibility (HET) proteins	PF06985, 07217, 17,108	72	10.5	753	57.9	1.94	1.24	1.56	13	59	81.9
aminotransferases	PF01490	49	13.8	675	51.9	1.47	1.18	1.25	21	28	57.1
amino acid permeases	PF00324	48	13.1	630	48.5	1.17	1.10	1.06	30	18	37.5
amidases	PF01979, 04909	37	17.0	629	48.4	1.93	1.20	1.62	12	25	67.6
acetytransferase	PF00583, 00797, 13,302, 13,523	49	12.2	600	46.2	1.26	1.10	1.15	23	26	53.1
DEAD-box helicases	PF00270	42	13.5	567	43.6	0.97	0.96	1.01	33	9	21.4
NmrA-like proteins, NAD-binding negative regulators of GATA-binding proteins	PF05368	41	13.5	552	42.5	3.01	1.11	2.70	10	31	75.6
DnaJ molecular chaperone	PF00226	42	12.8	537	41.3	1.02	0.99	1.04	19	23	54.8
RRM_1 RNA binding proteins	PF00076	42	12.8	537	41.3	1.00	0.99	1.01	40	2	4.8

^a^ -ratio of the number of genes in all species belonging to one of the *Trichoderma* sections or clades

^b^ - genes which are present in one a single copy per cluster

^c^ - genes that occur in more than one copy per cluster in at least one species

^d^ - percentage of clusters containing multiple genes

A comparison of the clusters found in *Trichoderma* to the other 14 Sordariomycetes showed that 2584 gene clusters were present in all *Trichoderma* spp. and at least one of the other 14 Sordariomycetes (Table 5; Additional file 6). Of them, eighty-six clusters were shared exclusively between *Trichoderma* and the entomoparasitic Hypocreales, and 700 clusters shared only between *Trichoderma* and the Nectriaceae. Genes encoding purine/uridine nucleoside phosphorylases, and CBM1-type cellulose binding domains were enriched in *Trichoderma,* and genes containing ankyrin-containing proteins were also significantly more abundant although they did not gain scientific support with respect to the phytopathogens. The majority of the other gene families were significantly more abundant in *Trichoderma* than in the entomopathogenes, but many of them less abundant than in the phytopathogens (Table 5; Additional file 7). Some gene families (HET- and NACHT domain proteins, AB-hydrolases, Zn2Cys6 transcriptional regulators, FAD dependent oxidases, AAA+ ATPases, transporter of the major facilitator superfamily, sugar transporters, cytochrome P450 monooxygenases and 2-oxoglutarate-dependent (FeII)-dioxygenases) were significantly more abundant in *Trichoderma* than in the entomopathogenes but clearly less abundant than in the phytopathogenes (Table 5). Genes encoding proteins with CFEM-domains were present in a lower number in *Trichoderma* than the entomo- and phytopathogenes.

**Table 5:**
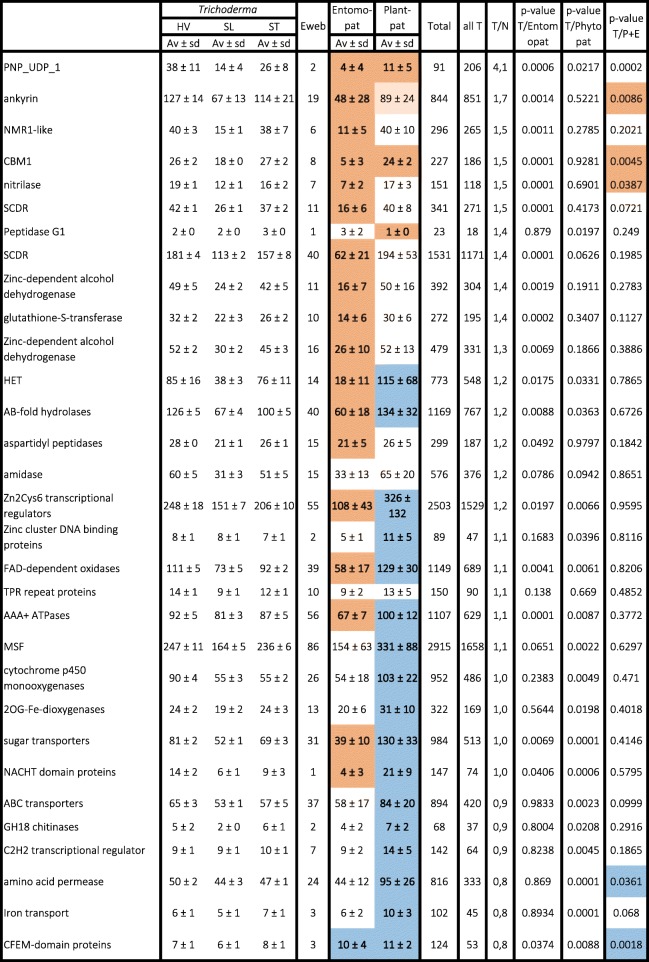
OrthoMCL clusters shared between *Trichoderma* and other Sordariomycetes Fungi

Entomopat. - six species of Entomopathogenic Hypocreales, Plant pat. - five species of plant pathogenic Hypocreales, Eweb - *Escovopsis weberi*, Av ± sd – average ± standard deviation; For strain abbreviations, see Methods. T - *Trichoderma*, N, Nectriaceae. PFAM categories printed in bold specify those that are significantly (ANOVA coupled with Dunnett’s post-test, P < 0.05) different compared with *Trichoderma* species

Brown background: enriched in *Trichoderma*; blue background: less abundant in *Trichoderma*

### The *Trichoderma* core genome

**Fig. 4:**
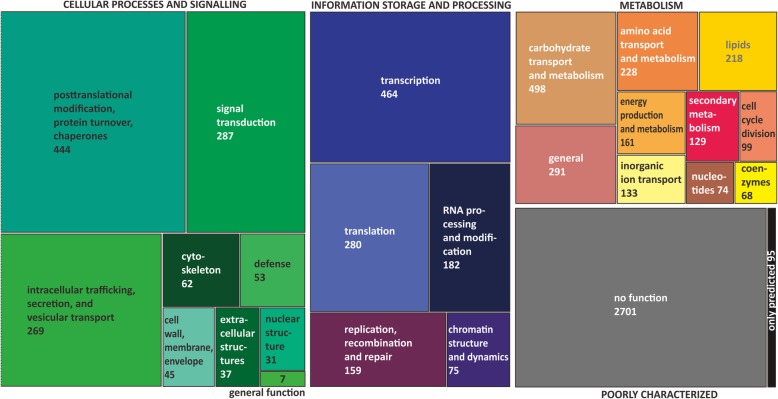
The structure of *Trichoderma* core genomes as revealed based on 13 strains. The number of genes of the core genome for which a KOG classification was obtained. The total number of genes in the core genome is 7000. The size of the boxes represents the abundance of the genes within the main KOG classifications (Cellular processes and signaling – green shades; Information storage and processing – violet shades; Metabolism – reddish shades; Poorly characterized -Grey shade. Predicted ORFs are shown in black). The numbers specify the numbers of core genome genes that belong to the respective functional groups

Exactly seven thousand genes had orthologs in all twelve *Trichoderma* species, and therefore represent the *Trichoderma* core genome. The automatically predicted encoded proteins were manually curated (Additional file 8) and at least a putative function based on a conserved protein domain could be attributed to 4413 of them. Using the KOG (eukaryotic orthologous groups) classification scheme [35], the genes classified as “metabolism” (1809) and “poorly characterized” (2587) constituted the two largest groups (Fig. 4). At the level of individual KOG families, “posttranslational modification, protein turnover and chaperones”, “transcription” and “carbohydrate transport and metabolism” contained more than 400 genes (Fig. 4). Genes encoding glycoside hydrolases (191) and fungal specific Zn_2_Cys_6_ transcription factors (173) were the most abundant protein families in the *Trichoderma* core genome (Table 5), followed by glycoside transferases [36], and C2H2-type transcription factors (45). Among the group of proteins for which only a general function could be predicted, solute transporters of the major facilitator superfamily (151), and short-chain dehydrogenases/reductases [37] contributed to a major part of the KOG group “Metabolism”.

**Fig. 5:**
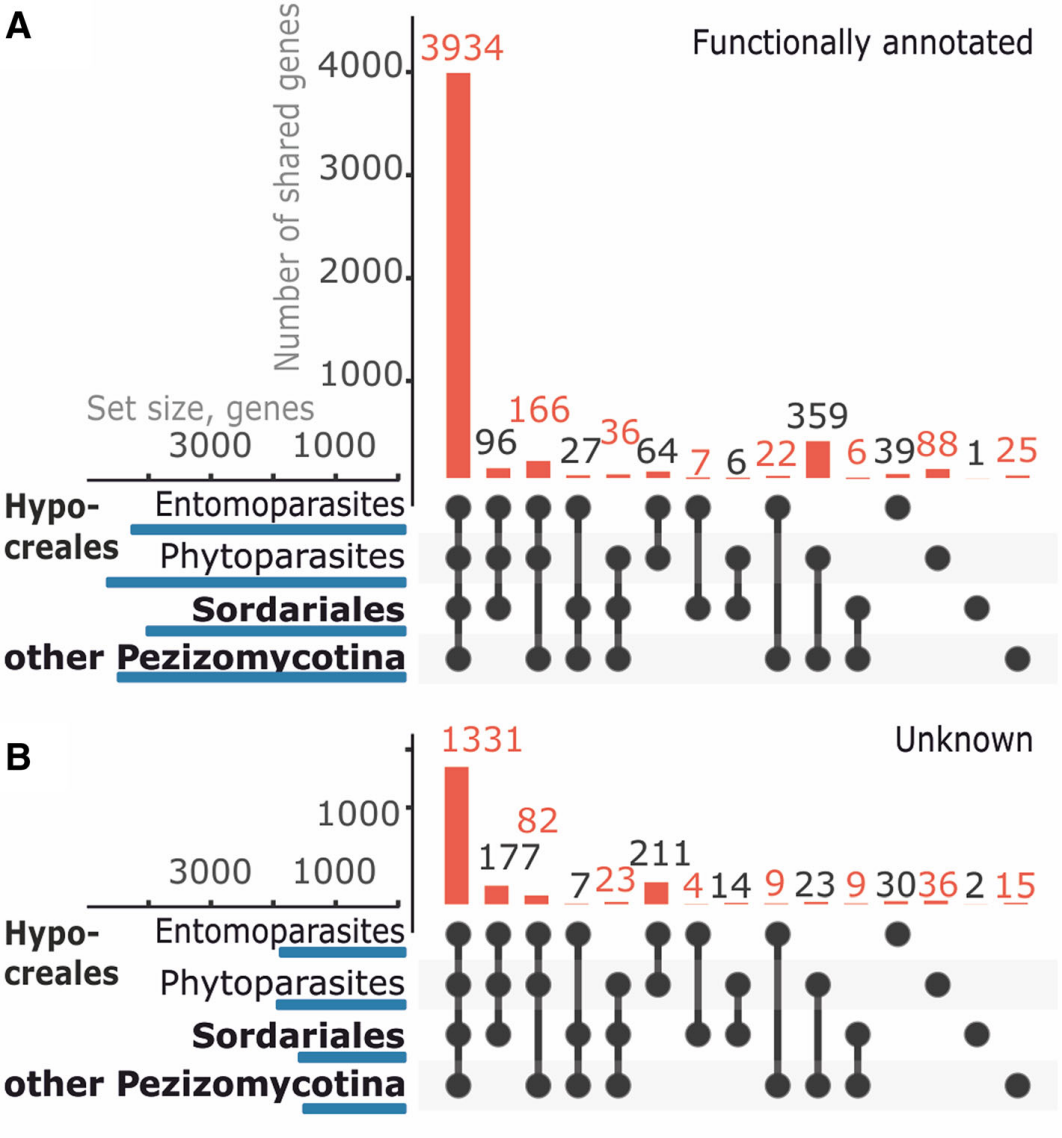
The share of *Trichoderma* core genome with genomes of other fungi. Genes of the *Trichoderma* core genome which have orthologs in other fungi (**a**, functionally annotated genes; **b**, unknown genes). The analysis was performed with Intervene [39]. The vertical bars and numbers indicate the number of genes that are shared by the fungal groups as indicated by the circles below the graph. The horizontal bar over the fungal groups indicates the total number of genes with orthologs in *Trichoderma.* Hypocreales entomoparasites were estimated based on the genomes of *Beauveria bassiana*, *Cordyceps militaris*, *Metarhizium acridum* and *M. robertsii*; Hypocreales phytoparasites were estimated based on the genomes of *Fusarium graminearum*, *Nectria haematococca*, and *F. verticillioides*; for Sordariales the genomes of *Neurospora crassa* and *Chaetomium globosum* were used. Other Pezizomycotina were assessed based on the analysis of the genomes of *Chochliobolus heterostrophus*, *Exophiala xenobiotica*, *Aspergillus fumigatus*, *A. oryzae* and *Oidiodendron maius* (see methods for details)

We compared the *Trichoderma* core genome to the complete genome of other fungi (nine of the Sordariomycetes investigated above, two species from the Eurotiomycetes, one of the Dothidiomycetes and one of the Leotiomycetes; see Methods) using a reciprocal BLAST approach and Intervene [38] (Fig. 5). This showed that 3934 of those 4413 genes of the core genome, for which a putative function could be identified during manual annotation, had orthologs in all of them (Fig. 5). In addition, 642 were shared between a subset of the fungal orders and families tested. The largest number (359) was shared between *Trichoderma* and all other fungi except for the entomoparasites, and 166 genes were only present in the Sordariomycetes but not in fungi from other classes.

A similar search for the functionally uncharacterized proteins revealed that 1331 of them were shared with all other fungi. The number of those shared only between some orders or families suggests a phylogenetic relationship: 211 of them were present only in the Hypocreaceae, and 177 in all Sordariomycetes (Fig. 5).

We conclude from these data that 80.7% of the genes encoding functionally predictable proteins and 67.4% of the genes encoding functionally not predictable proteins in the *Trichoderma* are already been present in the ancestor of Eurotiomycetes and Sordariomycetes and are therefore at least 250 million years old.

Comparing the intraspecific genome differences between the two isolates of *T. harzianum* showed that 1699 genes of *T. harzianum*^T^ (12%) were absent from the other strain, and 1419 genes present in the latter (10.1%) absent from the type strain. Most of these genes encoded orphan proteins for the species, and a function could only be predicted for 158 and 160 genes in *T. harzianum*^T^ and *T. harzianum* TR247, respectively. Their properties are described in Additional file 9.

We also compared the genomes of *T. longibrachiatum* and *T. citrinoviride -* the two species that are more frequently encountered as opportunistic pathogens of immunocomproized humans [40] - and identified 94 genes that were only present in these two species but absent from all others and could therefore belinked for their pathogenicity (Additional file 10).

105 genes of the core genome were present in all 12 *Trichoderma* species but not found in any other fungus. They thus fulfil the criterium of “genus specific orphans” and we will use the term “orphans” for them further throughout the manuscript. No function could be predicted for any of these genes.

### Gene expansion and contraction during evolution of *Trichoderma* species

**Fig. 6:**
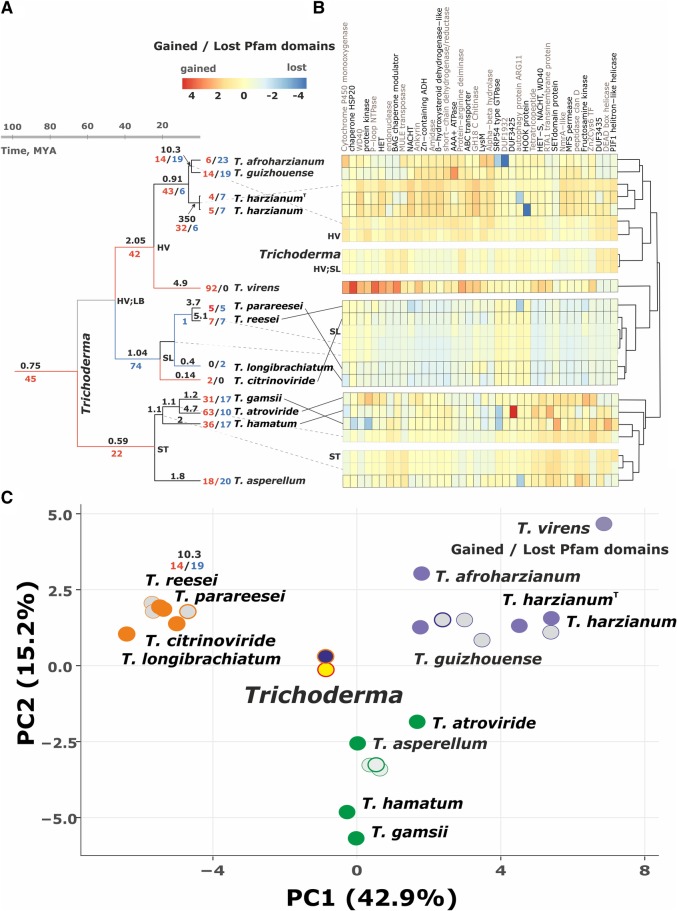
Genome evolution in *Trichoderma.* **a** time scaled evolutionary tree: red branches indicate only gains; blue branches only losses; black branches both gains and losses. Numbers over the branches indicate the number of gene changes per Mya; numbers below the branches indicate the number of gains (red) and losses (blue). **b** Heat map representing Pfam domains identifiend for OrthoMCL clusters that we gained or lost in the course of *Trichoderma* evolution. Framed rectangules correspond to extant species. Pale color used for hypothetical taxonomic units (HTUs, ancestral states). **c**. Principal component analysis based on the number of genes per each Pfam group that have been influenced by gene gain and loss in *Trichoderma*. Filled cicles correspond to extant *Trichoderma* species as shown on A. Grey circles correspond to HTUs per each infrageneric group (see **a** and **b**). Bold lined circles show the ancestral node for the respective section or clade. Circles with red/yellow and orangy/blue color show the ancestral node for the genis *Trichoderma* and SL/HV groups

We used the likelihood approach implemented in CAFÉ to identify individual gene families that evolved at rates of gain or loss that were significantly higher than the genome-wide averages in *Trichoderma* [41]. As shown in Fig. 6a, the origin of *Trichoderma* from its ancestor started with significant gene expansion (13 gene clusters comprising 45 genes gained, and none lost), and this expansion continued in the ancestors of ST (22 genes) and HV (42 genes) (Fig. 6). The rate of gene changes per mya increased significantly during the evolution of HV. The highest number of gene families gained by the *Trichoderma* ancestor were those encoding HET/NACHT and ankyrin proteins, and these two groups became further enriched during the evolution of HV and ST (Fig. 6b). The ancestor of SL, on the other hand, lost 74 genes and did not gain any. Interestingly, they also comprised a high number of genes for ankyrin and HET/NACHT domain proteins.

While these data show that the origin the genus *Trichoderma* and two of its clades/sections (HV, ST) underwent strong gene expansion whereas SL exhibits significant gene contraction, a deeper look into the gene evolution at the level of individual species revealed a mosaic of gain and loss events (Fig. 6a and b). Exceptions were *T. longibrachiatum* which shows only gene losses (but these data must be viewed with caution because of the higher incompleteness of its genome; see above), and *T. citrinoviride* which displays only gains. These data suggest that the extant taxa of *Trichoderma* are reforming their genomes at an increased rate, which is particularly reflected in *T. harzianum* because the two isolates of this species differed remarkably in their gene loss and gain.

The principal component analysis revealed that the tree different strategies in gene gain and loss that are characteristic for each section or clade (Fig. 6c). As all the tested species are nutritionally versatile, common and cosmopolitan, this pattern of group-specific evolution points to the importance of the core genome is the basis for the generalism.

Since the evolution of the *Trichoderma* genomes from their ancestor from 120 (±21) to 66 (±15) mya occured entirely by gene expansion (no gene losses revealed by the CAFÉ analysis, Figure 6a,b), we wondered whether this was due to a small genome in its putative ancestor. We therefore extended the CAFE analysis to all available *Hypocreales* genomes. Unfortunately, at the 99% probability used for *Trichoderma*, this analysis yielded no data which is probably due to the insufficient number of genomes that are currently available for the predictions over such long evolutionary interval. Reducing the probability level to 95%, however, revealed that the evolution after the split from the entomoparasite branch (184.6 ± 8 mya; see Fig. 3) and the obligate mycoparasite *Escovopsis weberi* (119.8 ± 21 mya) was accompanied by a total of 23 gain losses and only a single gain (Additional file 11). The ancestors of the genus *Trichoderma* may therefore have indeed been subject to a significant genome contraction.

### The *Trichoderma* genomes reveals the potential for heterothallic sexual reproduction

Most species of *Trichoderma* are found in nature in their sexual form (teleomorph) [8, 9], although the most generalist species are frequently and some even exclusively been isolated as anamorphs. Among the strains investigated in this paper, teleomorphs were only known for *T. reesei* (most frequent), *T. citrinoviride* (frequent)*, T. virens* (very rarely) and *T. atroviride* (rarely) [42–44]. Population genetic evidence for the absence of sexual recombination has been shown for *T. longibrachiatum, T. parareesei, T. harzianum* and *T. afroharzianum* [29, 45]. The structure of the population of other species is not known. We therefore looked for the presence of mating type genes in the thirteen strains. As can be seen in Table 6, we found either MAT1–1 or MAT1–2 idiomorphs in all of them, consistent with the view that *Trichoderma* is heterothallic. The distribution of the known sexually recombining and non-recombining species investigated in this study on the phylogenetic tree suggests that the gain or loss of this trait occurred several times during the evolution of *Trichoderma*.

**Table 6:** Mating type genes in *Trichoderma*

	mating protein	mating protein	mating protein	mating protein
	MAT1-2-1	MAT1-1-1	MAT 1-1-2	MAT 1-1-3
*T. reesei*	*124341*			
*T. longibrachiatum*		1427955	1467528	1388533
*T. citrinoviride*	*1107806*			
*T. parareesei*		-^b^	-^b^	OTA08401
*T. harzianum* ^*T*^	*104176*			
*T. harzianum*		434806	863060	863056
*T. guizhouense*	OPB38549			
*T. afroharzianum*	KKO 5631			
*T. virens*	60622			
*T. atroviride*	33998			
*T. gamsii*	TGAM01v2_08385			
*T. asperellum*		64910	158842	451243
*T. hamatum*		12232	12231^a^	12231^a^

^a^ annotated as one protein

^b^ no ortholog detected by Blastp against NCBI database

Sensing of a potential mating partner is a prerequisite for sexual reproduction and fulfilled by the pheromone system [46]. The genes involved in this process were found in all *Trichoderma* spp. and are given in Additional file 12.

### Major aspects of *Trichoderma* metabolism

#### Carbon metabolism

Carbon metabolism of *Trichoderma* has so far mainly been studied in *T. reesei* only and with respect to the catabolism of hemicellulose and pectin monomers [39, 47, 48]. We have therefore manually annotated all genes of the core genome that are putatively involved in carbon metabolism. The majority of these genes has already been described in detail for *T. reesei*, *T. atroviride* and *T. virens*, and we refrain from repeating these data here [25]. Yet we detected some novel features, such as the presence of an extracellular glucose oxidase, D-xylulose-5-phosphate/D-fructose-6-phosphate ketolases, enzymes for D-erythroascorbic acid biosynthesis, and a glutathione-linked methanol degradation pathway. These findings are described in some detail in Additional file 13.

#### Extracellular polymer hydrolysis

**Fig. 7:**
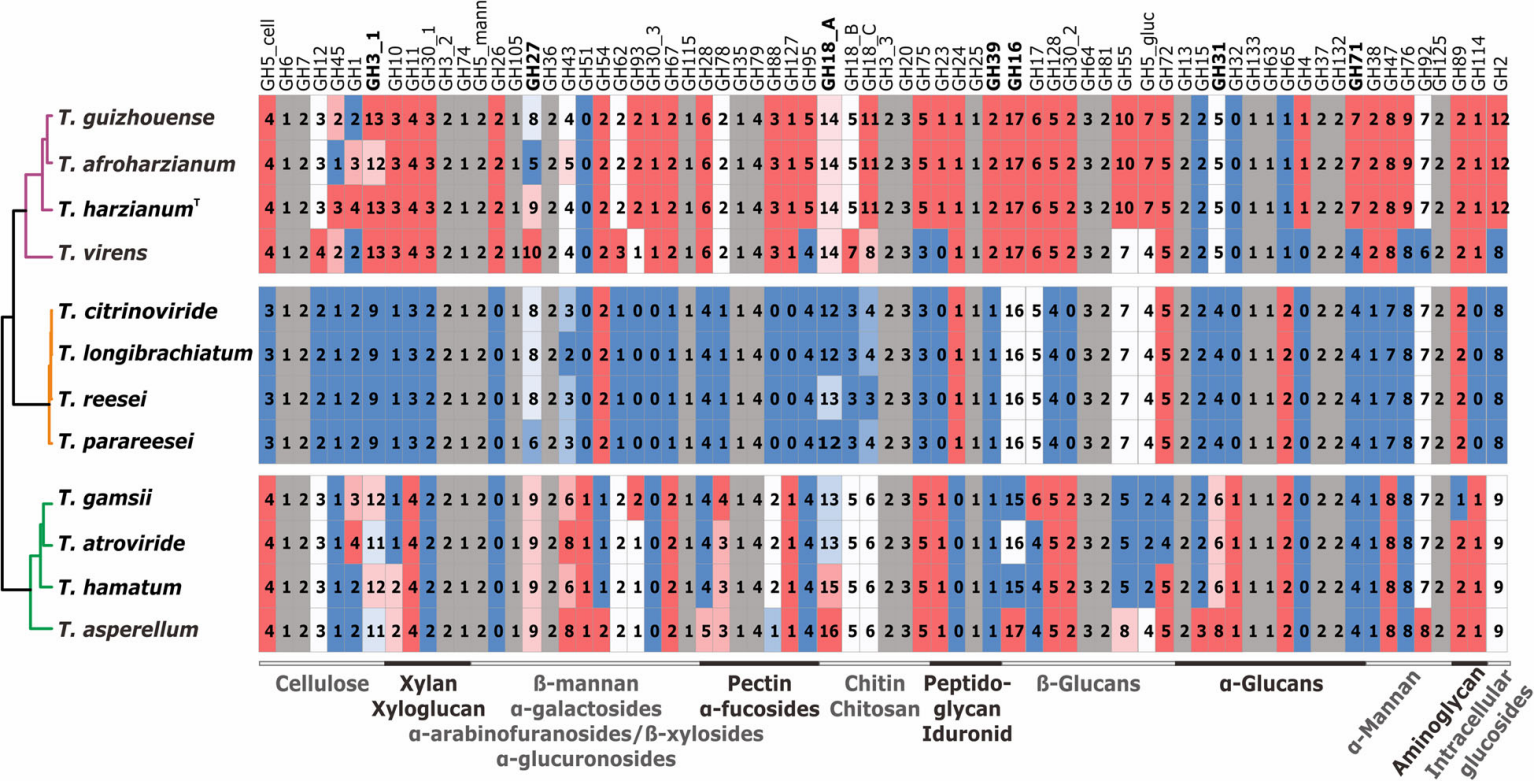
The glycosyl hydrolase (GH) inventory of *Trichoderma*. GHs are ordered according to their substrate (in broader sense), which is given on the right side. GHs which contain enzymes that act on different substrates (GH3, GH5, GH30) are indicated by extended numbers. GH18 chitinases are grouped into A (no binding domain), B (attached CBM1) and C (attached GH18 and/or GH50; see Fig. 8). Numbers mean the number of genes belonging to the respective GH family in their genomes. The cladogram on the left is based on the total number of genes per each GH family, complete linkage, Eucleadian distance

**Fig. 8:**
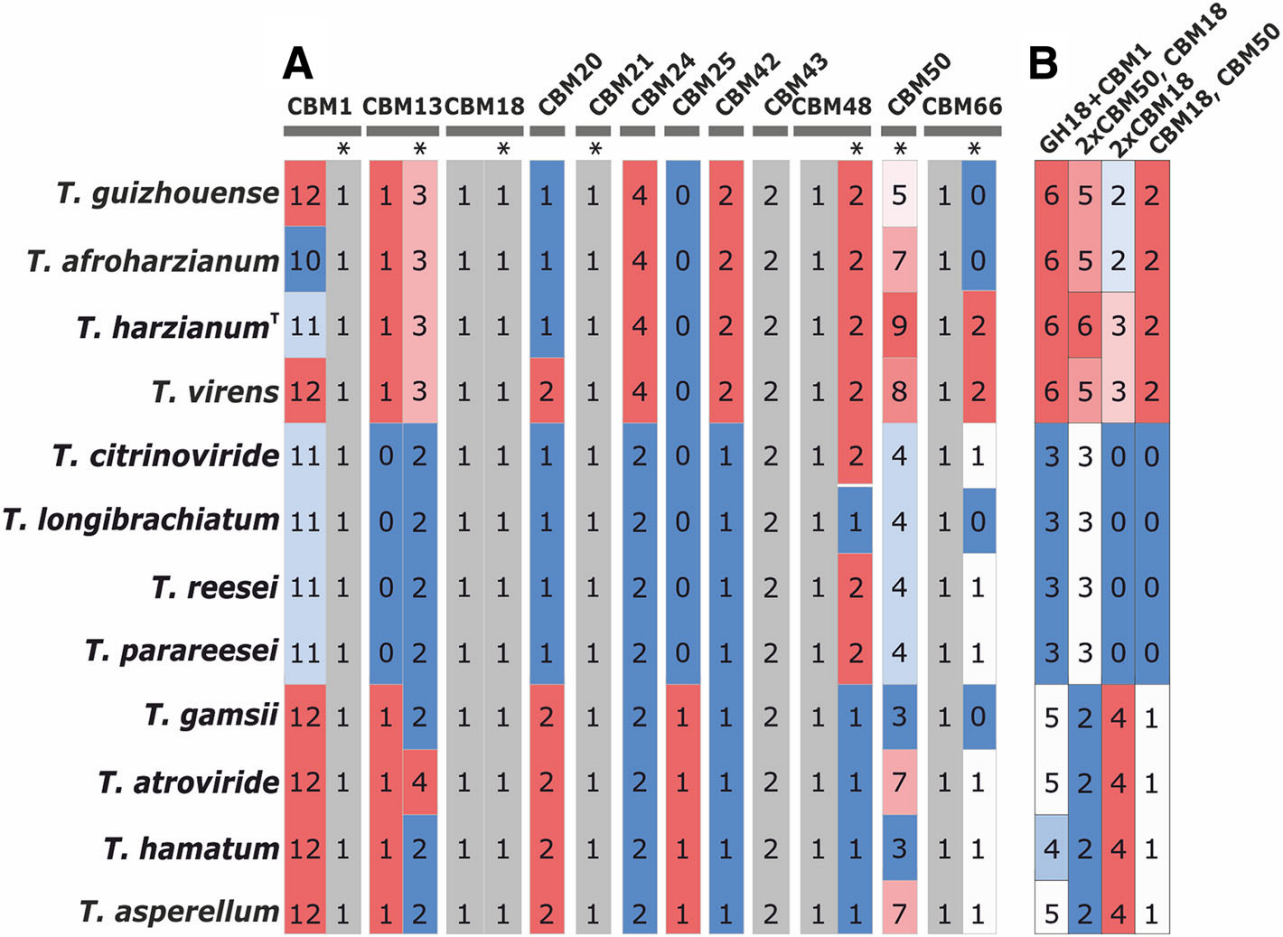
Type and presence of carbohydrate binding domains in *Trichoderma*. **a** The summary of domains: those columns marked with an asterisk indicate individual domains, i.e. domains which occur as separate proteins and are not attached to another enzyme. **b** Patterns of CBMs in GH18 chitinases

A unique feature of filamentous fungi is that they live in their mostly macromolecular substrates, and therefore must have efficient extracellular systems for the hydrolysis of the respective polymers (mainly polysaccharides and proteins). In *Trichoderma*, this is nicely reflected in the glycoside hydrolases (GHs) which comprise one the most abundant groups of its genome. In addition, accessory enzymes for the GHs (polysaccharide lyases, polysaccharide binding proteins, carbohydrate esterases, and auxiliary oxidative enzymes), which are classified in the CAZy (carbohydrate active enzymes) database [49] are required to aid in the hydrolysis of the respective polymeric substrates. The distribution of these genes in individual *Trichoderma* taxa is shown in Figs. 7 and 8, Additional file 14:.as can be seen, *Trichoderma* comprises between 269 and 361 CAZyme genes (glycoside transferases not counted), of which 195 were found in the core genome. GHs accounted for more than 50% of the CAZymes, GH18 chitinases, GH16 ß-1,3/1,4-glucanases and GH3 ß-glycosidases being present in the highest numbers. When the GHs are sorted according to the type of their polysaccharide substrates, GHs acting on chitin and ß-glucan again comprised the highest numbers, followed by enzymes acting on α-mannan (Fig. 7). Many of the latter are probably involved in cell wall and glycoprotein modification. The function of these genes and their distribution in the 12 *Trichoderma* species is described in detail in Additional file 14. Interestingly, the composition of CAZomes (estimated based on the respecive GH families) is almost invariable in SL, although the phylogenetic analysis revealed genetic distances between species in this section to be similar to those between species in other groups (compare the CAZyme-based cladogram on Fig. 7 and the phylogram on Fig. 3). The CAZymes of HV is significantly enriched compared to ST and SL. This enrichment is not biased towards a special substrate.

Apart of polysaccharides, proteins hydrolyzed by various proteases provide a major nutritional source for fungi. Some of protease families are also important for the digestion of proteins secreted by competing organisms [50–52] or hosts. We screened the secretome of the 12 *Trichoderma* genomes for proteases using the MEROPS database (see Methods for details). This demonstrated the presence of A1 aspartyl proteases, G1 eqolisins (previously termed “pepstatin-insensitive aspartyl proteases”), C13 legumain-type cysteine proteases, eight metalloprotease families (InhA-like peptidases, M6; carboxypeptidases, M14; glutamate carboxypeptidases, M20; methionine aminopeptidases, M24; aminopeptidase Y, M28; deuterolysin, M35 and fungalysin, M36), and six families of serine proteases (S1 chymotrypsins, S8 subtilisins, S10 carboxypeptidases, S28 an S51 dipeptidases, and S53 sedolisins) in *Trichoderma* (Additional file 15). Aspartyl proteases, subtilisins, sedolisins, and aminopeptidase Y were present in the highest numbers of isoenzymes. Family S10 was particularly abundant in HV, and S53 in HV and ST. In summary, however, the number of *Trichoderma* proteases is comparable to that of many other fungi [50–52], and we found no protease family that was specifically expanded or contracted in *Trichoderma*. Proteases have been speculated to be a component allowing niche differentiation between the ascomycetes and the basidiomycetes, particularly towards adaptation to pathogenicity by the former [52]. However, our data suggest that the primeval proteolytic arsenal of *Trichoderma* was sufficient for the acquisition of the mycoparasitic lifestyle and its more recent expansion towards generalism.

#### Secondary metabolism

Secondary metabolites (SM) are an intrinsic feature of most Pezizomycotina, because they participate in cellular signalling, competition, pathogenicity, and metal ion uptake [53]. *Trichoderma* too has been shown to be a proliferic producer of SMs [54, 55]. Unfortunately, the genes encoding these SMs and even the species identity of the SM producing isolates are in most cases unknown. We identified 10–25 polyketide synthase (PKS), 12–34 non-ribosomal polypeptide synthetase (NRPS)-, and 6–14 terpenoid synthase (TS) encoding genes in the 12 species (see Additional files 16 and 17), of which 6 PKS, 10 NRPS and 3 TS genes were present in the core genome.

In contrast to PKS, NRPS and TS, *Trichoderma* seems not to synthesize alkaloids, as we could not find the genes encoding the precursor dimethylallyl tryptophan synthases (DMATS; [56]) in any of studied genomes.

#### Small cysteine-rich secreted proteins

Fungi have developed several families of small, secreted proteins that are characterized by an enhanced content of cysteines, and which are believed to function in various ways in the communication between the fungi and other organisms [57]. Three different protein families can be distinguished in this group: small (< 300 amino acids) secreted and cysteine rich proteins (SSCPs); hydrophobins; and cerato-platanins (Table 7). The number of so detected SSCPs was surprisingly diverse (Additional file 18), but the variation was species- and not section-specific. *Trichoderma* is rich in hydrophobins, ranging from 7 in all species of section SL to 16 in *T. atroviride*. Six of them are conserved across all twelve *Trichoderma* species (see Additional file 18). As for cerato-platanin proteins, which represent fungal-specific, small and secreted proteins that are believed to be important for interaction with other organisms and eliciting defense reactions in plants [58–60], three genes (*epl1, epl2, epl3)* are present in the core genome. A detailed description of the small cysteine rich proteins is presented in Additional file 18.

**Table 7:** Number and types of small secreted cystein-rich proteins in *Trichoderma*

		SSCPs	HFBs		Cerato-platanins
			class II	pseudo-class I	
SL	*T. reesei*	39	7		3
	*T. longibrachiatum*	89	7		3
	*T. citrinoviride*	50	7		3
	*T. parareesei*	27	7		3
HV	*T. harzianum*	113	12	3	3
	*T. afroharzianum*	66	9	3	3
	*T. guizhouense*	44	10	2	3
	*T. virens*	65	12	3	3
ST	*T. atroviride*	75	13	3	3
	*T. gamsii*	42	12	3	3
	*T. asperellum*	125	11	3	3
	*T. hamatum*	62	11	3	3

#### *Trichoderma* orphan genes

The *Trichoderma* core genome contained 105 orphan genes (vide supra; Additional file 19). While they comprised only 1.5% of the genes in the core genome, orphan genes restricted to sections/clades or evensingle species were much more abundant (on the average 17.4, 13.0 and 10.1% in SL, ST and HV, respectively; and even higher within the pool of species-specific genes (see also Additional file 3).

**Fig. 9:**
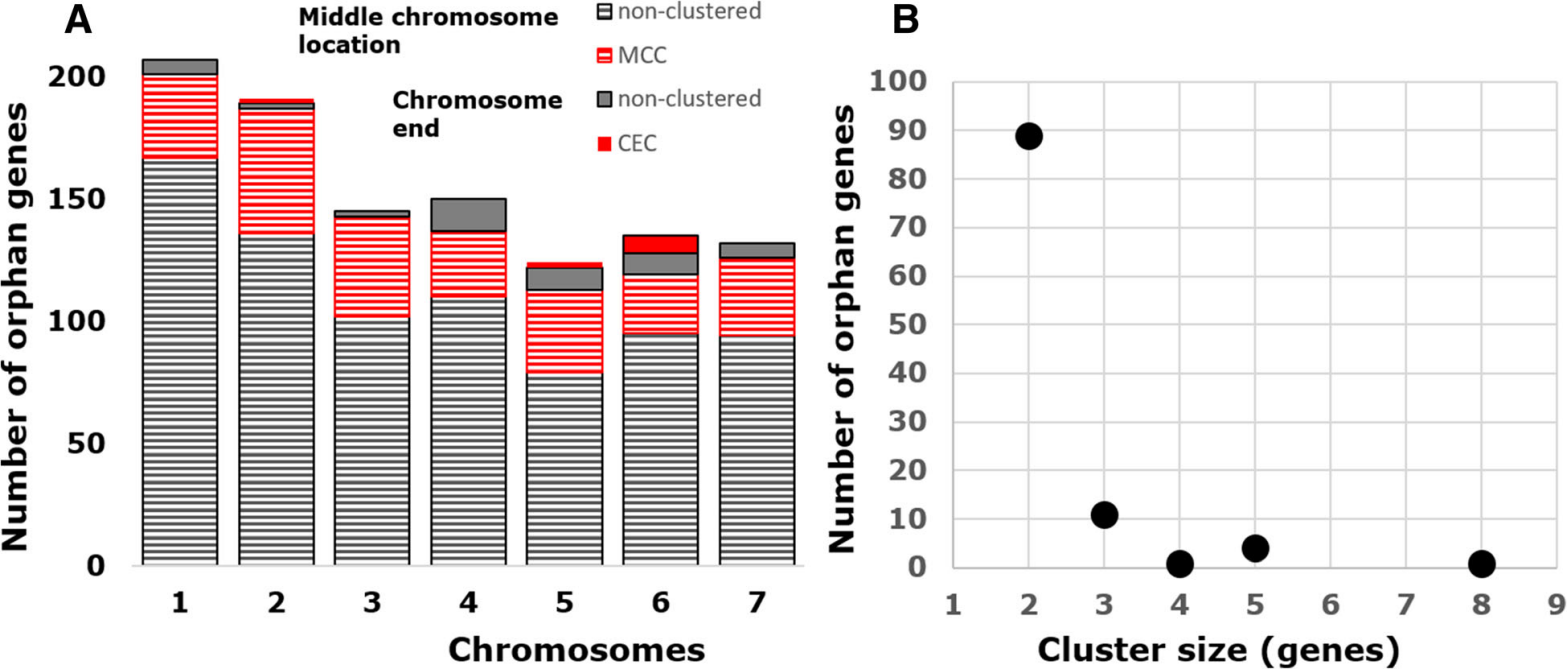
Numbers and clusters of orphan genes in the chromosomes of *T. reesei*. **a** Numbers and clusters; “chromosome end” specifies those orphans that are located with 100 kb from either chromosome end; “middle” specifies those that are located in the remaining area. **b** Number of orphan genes in clusters of varying size. The *T. reesei* chromosomes, published by Li et al. [27] were used for these investigations

Subjecting the orphans to analysis in the conserved domain database [61] failed to detect any known domain. Orphans have been shown to occur in gene clusters and to be enriched in subtelomeric regions in *Plasmodium*, yeast, *Aspergillus* and *Neurospora* [62–64]. To test whether this is also the case for *Trichoderma,* we made use of the complete annotated chromosomes of *T. reesei* [26, 27], and mapped its 1126 orphans on them (Additional file 19). Most of them occurred as single genes, and less than 10% of them were located within 100 kb from the chromosome ends (Additional file 19). Twentyfive to 30% of the orphan genes indeed occurred in gene pairs or clusters which in majority comprised two, but sometimes more and in a single case even eight genes. The proportion of the clusters that occurred in subtelomeric regions was again lower than 10% (Fig. 9; Additional file 19), suggesting that – similarly to the single orphan genes - the clustered orphans of *T. reesei* are not enriched near the chromosome ends. Interestingly, none of the *T. reesei* orphan genes that were present in the core genome was located near the chromosome ends, suggesting that the latter location is a species- or clade-specific feature. We however note that in several cases single genes in the core genome occurred as doublettes or triplets in *T. reesei*.

To analyse the evolution of the orphans, we measured the selection pressure acting on them by calculating the ratio of non-synonymous and synonymous amino acid substitution (dN/dS) for 53 orphan genes that are present in the *Trichoderma* core genome and whose nucleotide sequence could be unambiguously aligned. A dN/dS = 1 would indicate neutral evolution and dN/dS < 1 can be interpreted as evidence for purifying selection [65]. The data obtained (Additional file 20) show that most of these genes is under strong purifying selection. An assessment of dN/dS for the clade-specific orphans was not possible, because their nucleotide sequences were too polymorphic to be aligned.

## Discussion

This study is based on the genome sequences of 12 of the most common *Trichoderma* species. Although this number represents only a few percent of *Trichoderma* spp. described today, the selected species are members of three phylogenetically distant sections and clades, and the results therefore enable a broader insight of the genus. Also, these species were most frequent in our own studies of soil or rhizosphere sampled in different geographic regions such as the Canary Islands, Sardinia, Columbia, Egypt, China, Israel, South-East Asia, Siberia and many others [6–8, 13] and may therefore be called cosmopolitan. Because several of the twelve species that were selected by this study are used as bioeffectors in biocontrol products against plant pathogenic fungi, stimulate plant growth and immunity, are opportunistic pathogens of immunocompromised humans and are causative agents of the green mold disease on mushroom farms [6, 13], they can be considered as environmental opportunists in a broad sense. Although species in the each of the sections and clades have unique morphological features, their overall ecological features are similar: they are mycoparasites, can feed on cellulolytic material, and can establish themselves in soil and colonize the rhizosphere. This may suggest that these species maintained the “opportunistic” features from a common ancestor what may be reflected in the core genome.

We have therefore investigated the evolution and the therefrom arosen changes in the gene inventory of the selected 12 species. Although all the genomes were still incomplete, the small predicted percentage of missing genes (2–5% for all species except *T. longibrachiatum*) makes it probable that we have identified all gene families that are relevant for the interpretations and conclusions in this paper. We particularly emphasize that the differences in gene numbers that we considered relevant were in most cases several folds higher than the number of putatively missed genes.

Our results reveal that the the mycoparasitic Hypocreales deversified between 100 and 140 mya, the ancestor of *Trichoderma* evolved around the time of the K-Pg, and the formation of the three infrageneric groups studied (ST, SL and HV) occured 40–45 mya after the K-Pg event. The uncertainty in chronological dating makes it impossible to decide whether the genus *Trichoderma* arose before or after K-Pg. However, we have recently proposed that the genus *Trichoderma* has obtained most of the genes encoding plant cell wall degrading CAZymes required for phytosaprotrophic growth, by the lateral gene transfer [11] that likely took place before the diversification into infrageneric groups. The most likely interpretation of these data is therefore that *Trichoderma* was one of the fungal genera that participated in the strong burst in fungal populations that fed on the decaying biomass of the plants killed by the K-Pg [66]. Whether or not this increase in the number of fungi stimulated mycoparasitism can only be speculated, but clearly a successful antagonism and the ability to kill its competitor may have aided *Trichoderma* in establishing a high population density on decaying plant biomass. Moreover, the ability to endoparasitise closely related species (up to adelphoparasitism) could favor host/parasite DNA exchanges and further contribute to the formation of the unique core genome of *Trichoderma* [11].

Despite the standard deviation in the dating of fungal phylogenies, our data strongly suggest that the evolution of the three *Trichoderma* sections/clades investigated in this study occured after the K-Pg event. The origin of extant species in the three sections/clades occured in the early oligocene (20–30 mya), a phase characterized by cooler seasons and a significant extinction of the invertebrate marine fauna [67]. It is intriguing that this split led to an increased rate of gene gain and genome expansion in HV, whereas the formation of SL was accompanied by a significant gene loss. Kelkar and Ochman [68] reported that Pezizomycotina genomes in the size of 25- to 75 Mb (which includes all *Trichoderma* spp. investigated in this study) exhibit a positive correlation between decreased genome size and increased genetic drift, and vice versa. On a first glance, this observation may not be applicable to genome contraction in SL, because it concerned genes from nearly all functional categories and thus was not specifically directed to support a certain trait. Alternatively, our results could be explained by the streamlining hypothesis [69], which considers selection for a more economical lifestyle as the major driving force for genome reduction. According to this model, the presence or absence of multiple genes for the same function may produce only a small effect on the performance of the organism and thus have only little benefits for the cell. Sun and Blanchard [70] considered that this scenario would most likely occur in relative stable environments where competition for nutrients is severe, and where a smaller genome has the ecological advantage of spending less energy for growth and development. We speculate that HV and ST – but not SL - used this alternative for further ecological success.

One of the hypotheses for this work was that gene families that were gained during *Trichoderma* evolution and are more abundant in *Trichoderma* than in other related fungi could give further insights about how this genus became an environmental opportunist. Gene families that were gained in highest number by *Trichoderma* were those encoding proteins with an ankyrin-repeat, proteins with a HET domain and MSF transporters. In addition, protein families that were present in higher numbers than in other Sordariomycetes were PNP_UDP_1 nucleotide phosphorylases, and NmrA-like transcriptional regulators.

The ankyrin repeats - tandemly repeated modules of about 33 amino acid that form two α-helices separated by a loop – are among the most common protein-protein interaction motifs known. They occur in a high number of proteins mainly from eukaryotes and have functions in cell cycle regulation, mitochondrial enzymes, cytoskeleton interactions, signal transduction and stress resistance [71, 72]. So far, proteins with ankyrin repeats have not been systematically characterized from Pezizomycotina, but an expansion of proteins containing ankyrin repeat proteins has been reported for the insect endosymbiotic bacterium *Wolbachia* [73]*.* Ankyrins have therefore been suggested to play an important role in endosymbiosis of this bacterium [73]. The higher number of proteins with this protein-protein interaction module in *Trichoderma* than in other fungi (with *Nectria* being the only exception) may suggest that its signalling and metabolic processes are more tightly coordinated than in other fungi which could ultimately result in enhanced fitness in its habitat.

Another group of proteins that made up for a significant portion of the genes gained by HV ans ST are the fungal HET (heterokaryon incompatibility) proteins. They have already received considerable attention of fungal genetisists because of their role as key players in recognition and response to non-self during cell fusion, which allows different individuals of the same species to maintain intergity and individuality [74–76]. HET proteins that contain an N-terminal HET effector domain, a central GTP binding site and a C-terminus consisting of highly conserved WD40 tandem repeats have been defined as HNWD protein family [77]. Lamacchia et al. [78] recognized that proteins of this family have similarity to pathogen-recognition receptors in plant and animals and proposed that these genes might also have a function in the recognition and response to other pathogenic species [78, 79]. With respect to *Trichoderma*, we extend this hypothesis by speculating that they could also play a role in recognition of mycoparasitic hosts, which is a challenging objective for further studies.

Apart of these two striking examples, the expansion of genes encoding NmrA-like proteins (which function as repressors of GATA-type transcription factors; [34]) and Zn_2_Cys_6_-transcriptional regulators in HV is also of interest, because we did not notice an expansion of protein kinase families. We therefore assume that speciation in this clade is accompanied by a diversification and fine tuning of transcriptional regulation, whereas regulation at the posttranscriptional level occurs mainly by the canonical signalling pathways in a similar way as in other fungi.

Based on the analysis of *T. reesei, T. virens* and *T. atroviride* it was previously concluded that the genus has only a small arsenal of secondary metabolite synthases [12]. The present comparison shows that this only true for the PKS and NRPS in species of SL. Compared with the *Aspergillus* spp., which are considered as being particularly rich in secondary metabolites [80], *T. harzianum* has a higher number of NRPS (twenty-nine). The number of PKS in *T. virens* is in the average [25] of those present in Aspergilli. In the case of terpenoid synthases, *Trichoderma* contains 12 to 17 genes and therefore clearly outnumbers the *Aspergillus* spp. that have only 2 to 10. However most of these secondary metabolite synthases – especially those for terpenoids - have not yet been characterized. The relation between the many secondary metabolites reported in *Trichoderma* and the genes responsible for their synthesis is therefore not known, which defines yet one more intriguing field for further studies.

We have also annotated the complete CAZome of all 12 *Trichoderma* species, which revealed the presence of some proteins like the GH4 α-glucosidases or the AA11 chitin monooxygenases that have not yet been described to occur *Trichoderma*. In addition, we detected that *Trichoderma* possesses a rich arsenal of carbohydrate-binding domains, which occur as fusions to GHs, CEs or AAs, but also as individual secreted proteins. The CBM1 cellulose-binding domain and the CBM50/LysM chitin/peptidoglycan binding domains have been already described in detail [81, 82], but we also found a high number of additional CBMs that putatively bind to starch, fructans and hemicelluloses. It therefore appears that *Trichoderma* makes significant use of these domains, and this could result in faster and more competitive degradation of the respective polymers. In these regards it is also of interest that HV possesses GH18 group C chitinases that contain both CBM18 as well as CBM50/LysM chitin binding domains, which have not yet been reported elsewhere. The possible differences in binding of CBM18 and CBM50/LysM to chitin are not known, however, which makes a speculation about the advantage of their arrangement in GH18 group C chitinases of HV *Trichoderma* difficult.

Finally, a striking feature in all *Trichoderma* genomes was the high number of orphan genes, of which only a very small number is also present in the core genome. The origin of orphan genes has been postulated to be either the consequence of gene duplication events and rearrangement processes followed by fast divergence, or of de novo evolution out of non-coding genomic regions [83]. Our data showed that - in the case of *T. reesei* - only a fifth of the orphan genes occured in clusters that could be indicative of gene duplications, and only a very small portion of orphans (clustered and non-clustered) occured near the telomeres, a frequent area for gene duplications. Our data therefore do not support gene duplication as the major mechanism for the emergence of orphan genes. The question whether the *Trichoderma* orphans originate de novo (see above) cannot be answered by our data. Published transcriptome data from *T. reesei* and *T. virens* [84, 85] show that about 40% of the orphan genes are indeed expressed, and therefore represent protogenes [86]. Our data suggest that the *Trichoderma* species-specific orphan genes evolve so fast that their sequences diverge beyond recognition, as already discussed for insects [87]. The biological merit, if any, needs further investigations to become understood, however.

## Conclusions

This paper highlights the evolution of twelve *Trichoderma* species that are most frequently observed in nature and which belong to three different *Trichoderma* sections/clades and documents the gene inventory of the core genome and the individual species. The data reveal a high genomic diversity both at the section and clade level and on the species-level, which is reflected by the fact that only 50–75% of the genes are conserved in all twelve species. The high polymorphism in ankyrin and HET genes, but also of such encoding transcription factors, enzymes for carbohydrate and secondary metabolism illustrates that *Trichoderma* belongs to those genera of fungi which constantly re-shape their genome for fast responses and successful competition in potentially novel habitats. These properties are exactly what one would also expect from an environmental opportunist and generalist.

The data presented in this paper will likely become a starting point for mining *Trichoderma* genomes for enzymes or secondary metabolites, and for selection of candidate genes for manipulating strains towards desired behaviour in their application. Sequencing and annotation of genomes of species outside the currently investigated clades will be facilitated by the curated protein identification encoded by the core *Trichoderma* genome. This may likely lead to the detection of still new features not seen in species from sections *Longibrachiatum*, and *Trichoderma* and in *Harzianum/Virens* clades.

Finally, our data raise the genus *Trichoderma* to the level of the few fungal taxa for which genome sequences of several different species are available, such as Aspergillus and Fusarium, and which strongly facilitated studies on various aspects of the molecular physiology of these fungi. Our data for *Trichoderma* now offers such a basis as well.

## Methods

### In silico screening for most common *Trichoderma* species

An *in-silico* screening for most common *Trichoderma* species whose nucleotide sequences are deposited in GenBank yielded 29,911 sequences for 292 species (April 2018). Sequences collected for undefined species (“cf. *Trichoderma*” or “*Trichoderma* sp.”), poorly characterized species (i.e. that are represented by less than 3 nucleotide sequences), or sequences arising from whole genome sequencing projects were excluded. *T. reesei* is a special case, because most of its sequences represented genes of only a single isolate (QM6a and its mutants), what is related to its industrial application. The total number of sequences from individual *T. reesei* isolates is estimated to be 30, which is rather small. *T. reesei* was nevertheless included in this study becuase its genome sequence and annotation were already available [16] and considered to be a good basis for comparison to the more abundant species of section SL.

### *Trichoderma* genomes

All but one (*T. harzianum* TR274) *Trichoderma* genome sequences were taken from JGI and NCBI databases (see “data access” below for numbers), and have been published [11, 12, 16, 17, 19, 22, 23].

*T. harzianum* TR274 has been isolated from soil in southeast of Brazil [29]. The genome was sequenced paired-end 2 × 250 bp using Miseq technology (Illumina™) and assembled with AllpathsLG [88] using maximum coverage of 120X. The genome was annotated using the Mycocosm annotation pipeline [31], and all data generated are available at the Mycocosm portal (https://genome.jgi.doe.gov/mycocosm/home).

#### Re-annotation of the *T. hamatum* GD12 genome

The *T. hamatum* genome is available in the public domain only in the form of assembled nucleotide scaffolds (accession number ANCB00000000.2). We performed structural annotation using the MAKER genome annotation pipeline v2.31.8 [89] with the gene predictor Augustus (http://bioinf.uni-greifswald.de/augustus/) trained with gene models from *Fusarium graminearum*. All proteins and transcripts from the *Trichoderma* ssp. analyzed in this study were used as gene model support. For functional annotation of translated proteins in the *T. hamatum* GD12, we performed InterProScan5 (http://www.ebi.ac.uk/interpro/) annotation, using stand-alone version 55 with the following embedded programs: SignalP4.1 [90], PFAM v.29 [91], Interpro [92] and GeneOntology (http://www.geneontology.org/).

#### Other fungal genomes analysed

The Ascomycota that were used in this study in comparison to *Trichoderma,* their habitats, taxonomic position and published genome sequences are given in Additional file 21.

### Annotation of the *Trichoderma* proteomes

We first searched all 13 *Trichoderma* genome databases for orthologs in the *T. reesei* QM6a and RUT C-30 genome by reciprocal blastp, using a treshhold of < E^-35^ (this value turned out to retrieve the highest percentage of hits that were confirmed by reciprocal blastp in a series of trials with different E treshhold values). Data obtained for *T. reesei* QM6a and RUT C-30 were combined and pruned to contain individual genes only once. The BLAST servers of the Joint Genome Institute were used for most *Trichoderma* spp. A local blastp for *T. parareesei* and *T. guizhouense* was established at the server of the Institute of Chemical, Environmental and Bioscience Engineering, TU Wien. For *T. gamsii*, *T. afroharzianum*, and *T. hamatum* no individual BLAST server was available, and their predicted proteome therefore re-assessed by blastp in the NCBI Blast server. The so predicted proteins were cross-checked by Pfam v. 29 [91] using a TimeLogic Decypher machine and an <E-^35^ treshold, and Interpro [92].

Conserved protein domains in proteins were further veryfied by Blastp against NCBI’s conserved domain database (https://www.ncbi.nlm.nih.gov/Structure/cdd/wrpsb.cgi; [61], using a treshhold of < E^-05^. Putative localization of proteins was analyzed using SignalP (for secreted proteins; http://www.cbs.dtu.dk/services/SignalP/), TargetP (for possible mitochondrial location; http://www.cbs.dtu.dk/services/TargetP/) and TMHMM (for preduction of transmembrane helixes in proteins; http://www.cbs.dtu.dk/services/TMHMM/). In all three methods, only hits with *p* < 0.05 were used.

In addition, we performed Ortho MCL clustering [93] with an inflation parameter of 1.5 on protein sequences from 26 predicted full proteomes (thirteen *Trichoderma* spp. and 13 from Hypocreales and Sordariomycete outgroups). A protein was considered specific to an organism subset if it was found at least in all but one of the organisms of the subset, but not in any organisms outside the subset.

### Identification of specific protein families

Annotation of the genes encoding carbohydrate active enzymes (CAZymes) in the 13 *Trichoderma* genomes was performed using the Carbohydrate-Active Enzyme database and CAZy nomenclature (http://www.cazy.org/), by comparing each protein model from the genome by the sequence similarity search tool (BLAST) to a collection of protein modules corresponding to catalytic and carbohydrate-binding modules derived from CAZy. Individual hits were then compared by HMMer to models corresponding to each CAZy family to allow an assignment of each identified protein.

Proteases were identified by analysis of the proteomes of the 13 strains in the MEROPs database (https://www.ebi.ac.uk/merops/) and the corresponding nomenclature used to specify them.

Identification of PKS, NRPS and terpenoid synthases was performed with Antismash [94] and SMURF (http://www.jcvi.org/smurf). Potential orthologs of PKS genes in different *Trichoderma* spp. were determined by phylogenetic analysis, using the KS domain (PKS) and adenylation domain (NRPS). The Maximum Likelihood method, based on the Poisson correction model [88], was used to infer the evolutionary history. Branches corresponding to partitions with a boostrap coefficient of < 50% (1000 replicates) are collapsed.

To identify SSCPs, the proteomes of the 13 *Trichoderma* strains were first filtered with Microsoft Excel for those that have a protein size less then 300 amino acids and contain ≥5% cysteines and the detected candidates then subjected to SignalP analysis [90]. Among this subset of proteins, hydrophobins were visually identified by the presence of 8 cysteines, of which C2/C3 and C6/C7 occured as pairs. Ceratoplatanins were identified by the presence of 4 cysteines and blastp against the NCBI database. The remaining proteins were considered as uncharacterized SSCPs.

### Analysis of genome completeness

To access the completeness of the genomes, we conducted a BUSCO v2 (Benchmarking Universal Single-Copy Orthologs) search our genomes for orthologues to each of 3725 Sordariomycete orthologous genes [32].

### Generation of a time-scaled phylogeny of the Hypocreaceae

We estimated the phylogeny of the 27 Ascomycota species in our analysis using the protein sequences of 638 orthologs present in single copy in all species, identified using Proteinortho5 [95]. For each set of orthologous proteins, we produced multiple alignments using MAFFT [96] with the auto settings and identified conserved alignment blocks using Gblocks v0.19b [97]. The final concatenated alignment used for phylogenetic reconstruction analysis consisted of 259,738 amino acid positions. Clade ages were estimated using the tool CladeAge [98] described in Matschiner et al. [99]. Four ancestral nodes were used for the time calibration: a common ancestral node of the order Hypocreales was calibrated for a central 95% range of 190–196 Mya [3], a common ancestral node between families Hypocreaceae, Ophiocordycipitaceae and Clavicipitaceae was calibrated for a central 95% range of 162–168 Mya [100], a common ancestral node of Clavicipitaceae crown group for a central 95% range of 114–120 Mya [100] and a common ancestral node of Nectriaceae crown group for a central 95% range of 122–128 Mya [3]. Species within these clades were forced to form a monophyletic group to constrain the tree topology. The selection of best amino acid substitution model was done using ProtTest 3 [171} based on BIC criterion. A MCMC analyses were carried out with a chain length of 20,000,000 sampling on every 1000 generation in BEAST V2.4.0 [98], using JTT I + G + F and the lognormal relaxed clock was used for determination of the clade ages. Their combined logs for the analyses for each dataset were diagnosed using Tracer v1.6 to confirm that the effective sample size is above 200 for the estimated parameters. In TreeAannotator v2.4.0 (in the BEAST package [98]), 25% of the first total trees were discarded, 0.9 was used as posterior probability limit and node heights were estimated using mean heights in order to obtain the maximum clade credibility tree. The final tree with node ages and an automatic reverse scale axis was visualized and obtained using FigTree v1.4.2 (http://tree.bio.ed.ac.uk/software/figtree/). Approximate 95% confidence interval was obtained by selecting “Height Highest Probable Density of 95%” for node bars in FigTree to show the age in the chronogram.

### Analysis of *Trichoderma* phylogeny

The nucleotide sequence of a fragment of the *rpb2* (RNA-polymerase II encoding gene) was retrieved from NCBI GenBank for 196 species of *Trichoderma*, and aligned. 808 nucleotides were then used for Bayesian analysis. Two independent MCMC runs were performed with 10 million generations and sampling frequency after each 100 generations; the first 800 trees have been removed. An earlier version of this tree, which does not make reference to the abundancy of species, has been published [7].

### Analysis of protein family evolution

The evolution of protein family size variation (expansion or contraction) was analyzed by CAFÉ [41] (using the orthoMCL table with an e-value ≤1e-20, and an inflation parameter of 1.5) with a *p*-value of 0.01 and applying a stochastic model of gene death and birth.

### Analysis of dN and dS

We estimated non-synonymous nucleotide substitutions (dN) and synonymous substitutions (dS) using PAML [101] with model M0 in pairwise mode implemented with custom shell scripts and calculated average dN/dS.

### Estimates of evolutionary divergence between protein sequences

Analyses were conducted using the JTT matrix-based model and the rate variation among sites was modeled with a gamma distribution (shape parameter = 4). The analysis involved 27 species, same used to build time-scaled phylogenetic tree. All positions containing gaps and missing data were eliminated. There was a total of 380,905 positions in the final dataset. Evolutionary analyses were conducted in MEGA7 [101].

### Data access

Genome assembly and annotations are available at at the JGI fungal genome portal MycoCosm [31] and are available at DDBJ/EMBL/GenBank under the following accessions: *T. reesei*, PRJNA225530; *T. parareesei*, LFMI00000000, *T. longibrachiatum*, MBDJ00000000; *T. citrinoviride*, MBDI00000000; *T. harzianum* CBS226.95, MBGI00000000; *T. harzianum* TR274, NQLC00000000; *T. guizhouense*, LVVK00000000; *T. afroharzianum*, JOKZ00000000; *T. virens*, PRJNA264113; *T. atroviride*, PRJNA164112; *T. gamsii*, JPDN00000000; *T. asperellum*, MBGH00000000; *T. hamatum*, ANCB00000000. The revised protein sequences and annotations of the *T. reesei* and *T. hamatum* genomes are included in the paper (Additional files 3 and 4).

## Additional files

Additional file 1: Abundancy of genes for *Trichoderma* species in GenBank. (XLSX 17 kb)

Additional file 2: Improvement of published genome annotations and verification of strains. (PDF 185 kb)

Additional file 3: Manually annotated genomes of *T. reesei* and *T. hamatum. (XLSX 982 kb)*

Additional file 4: Protein sequences of *T. hamatum,* fasta format. (TXT 5440 kb)

Additional file 5: Pairwise amino acid distances between *Trichoderma* and other *Sordariomycetes* fungi. (XLSX 16 kb)

Additional file 6: OrthoMCL clusters of proteins encoded by *Trichoderma* and other fungi. (XLSX 6578 kb)

Additional File 7: OrthoMCL clusters shared between *Trichoderma* and other Sordariomycete fungi. (XLSX 35 kb)

Additional file 8: The *Trichoderma* core genome. (XLSX 1010 kb)

Additional file 9: Intraspecific variation in *Trichoderma harzianum* as estimated based on the analysis of the two strains. (PDF 232 kb)

Additional file 10: Unique genes shared only between facultative pathogenic *Trichoderma* species. (PDF 165 kb)

Additional file 11: Gene clusters gained or lost in the evolution of *Hypocreaceae* before the origin of *Trichoderma. (XLSX 24 kb)*

Additional file 12: *Trichoderma* genes involved in sensing of the mating partner. (PDF 285 kb)

Additional file 13: Carbon metabolism in *Trichoderma. (PDF 224 kb)*

Additional file 14: Polysaccharide decomposition by *Trichoderma. (PDF 201 kb)*

Additional file 15: Extracellular proteolytic enzymes in *Trichoderma. (XLSX 19 kb)*

Additional file 16: Secondary metabolism genes of *Trichoderma. (PDF 318 kb)*

Additional file 17: PKS, NRPS, PKS-NRPS hybrids and terpenoid synthases in *Trichoderma. (XLSX 45 kb)*

Additional file 18: Small cysteine-rich secreted effector proteins in *Trichoderma. (PDF 210 kb)*

Additional file 19: Location of orphan genes and orphan gene clusters on the chromosomes of *T. reesei. (XLSX 40 kb)*

Additional file 20: Evolution of the orphan genes present in the *Trichoderma* core genome. (XLSX 13 kb)

Additional file 21: Ascomycete genomes that were used in this study as a comparison to Trichoderma. (XLSX 14 kb)
