# Supplementary material for: Evolution and comparative genomics of the most common Trichoderma species
Source: BMC Genomics. 2019 Jun 12;20:485. doi: 10.1186/s12864-019-5680-7 (PMC6560777; doi:10.1186/s12864-019-5680-7)
Supplement: Supplementary file 2 — Improvement of published genome annotations and verification of strains. (PDF 185 kb) [file 12864_2019_5680_MOESM2_ESM.pdf]

Improvement of published genome annotations and verification of strains

Contents

Strain verification 1

Improvement of the *T. reesei* genome annotation 1

Improvement of the *T. hamatum* genome annotation 1

Strain verification

Species identities of the strains used in this study were determined by sequence analysis of a diagnostic sequence of the long intron of the *tef1* gene encoding translation elongation factor 1-alpha as described in (Druzhinina *et al.*, 2010, Chaverri *et al.*, 2015). All but one species were found to be correctly identified. The genome of *T. afroharzianum* has been published as "*T. harzianum*" (Baroncelli *et al.*, 2015), but sequence analysis of the above specified *tef1* gene fragment clearly identified the isolate as *T. afroharzianum.*

Improvement of the *T. reesei* genome annotation

The genome of *T. reesei* QM6a, sequenced in 2004 (Martinez *et al.*, 2008), was compared to that of its mutant RUT C-30 (sequenced in 2009;(Le Crom *et al.*, 2009, Koike *et al.*, 2013)), and genes present in the latter but missing in the former (679 genes) included in its annotation. This combined *T. reesei* genome now contained 9,886, instead of the previously annotated 9,132 genes (Additional File 3).

In the later course of this work, we became aware of the paper by Li et al. (Li *et al.*, 2017), who used single-molecule real-time (SMRT) sequencing to detect even 10877 genes in *T. reesei*. A comparison of their annotation with that performed in the present study revealed that 823 of the 889 additional proteins detected in (Li *et al.*, 2017) were unidentified proteins. Two thirds of the functionally predicted 62 genes (=0.6 % of the genome annotated by us) encoded proteins putatively involved in nucleic acid, ribosome or mitochondrial metabolism. No proteins that would have significant impacted the results or interpretations of this study were detected, so we refrained from re-doing all analyses. Yet we introduced their data where this is necessary for interpretation of our results.

Improvement of the *T. hamatum* genome annotation

In addition, the genome sequence of *T. hamatum* GD12 (Studholme *et al.*, 2013), which was only available in the form of nucleotide scaffolds, was re-analysed (see Materials and Methods) and found to putatively contain 10,520 protein coding genes (Additional File 9).

References

Baroncelli R, Piaggeschi G, Fiorini L, Bertolini E, Zapparata A, Pe ME, Sarrocco S & Vannacci G (2015) Draft Whole-Genome Sequence of the Biocontrol Agent *Trichoderma harzianum* T6776. *Genome Announc* **3**.

Chaverri P, Branco-Rocha F, Jaklitsch W, Gazis R, Degenkolb T & Samuels GJ (2015) Systematics of the *Trichoderma harzianum* species complex and the re-identification of commercial biocontrol strains. *Mycologia* **107**: 558-590.
Druzhinina IS, Kubicek CP, Komon-Zelazowska M, Mulaw TB & Bissett J (2010) The *Trichoderma harzianum* demon: complex speciation history resulting in coexistence of hypothetical biological species, recent agamospecies and numerous relict lineages. *BMC Evol Biol* **10**: 94.
Koike H, Aerts A, Labutti K, Grigoriev I & Baker S (2013) Comparative Genomics Analysis of *Trichoderma reesei* Strains. *Industrial Biotechnology* **9**: 6.
Le Crom S, Schackwitz W, Pennacchio L*, et al.* (2009) Tracking the roots of cellulase hyperproduction by the fungus *Trichoderma reesei* using massively parallel DNA sequencing. *Proc Natl Acad Sci U S A* **106**: 16151-16156.
Li WC, Huang CH, Chen CL, Chuang YC, Tung SY & Wang TF (2017) *Trichoderma reesei* complete genome sequence, repeat-induced point mutation, and partitioning of CAZyme gene clusters. *Biotechnol Biofuels* **10**: 170.
Martinez D, Berka RM, Henrissat B*, et al.* (2008) Genome sequencing and analysis of the biomass-degrading fungus *Trichoderma reesei* (syn. *Hypocrea jecorina*). *Nat Biotechnol* **26**: 553-560.
Studholme DJ, Harris B, Le Cocq K, Winsbury R, Perera V, Ryder L, Ward JL, Beale MH, Thornton CR & Grant M (2013) Investigating the beneficial traits of *Trichoderma hamatum* GD12 for sustainable agriculture-insights from genomics. *Front Plant Sci* **4**: 258.
