# Supplementary material for: Evolution and comparative genomics of the most common Trichoderma species
Source: BMC Genomics. 2019 Jun 12;20:485. doi: 10.1186/s12864-019-5680-7 (PMC6560777; doi:10.1186/s12864-019-5680-7)
Supplement: Supplementary file 9 — Intraspecific variation in Trichoderma harzianum as estimated based on the analysis of the two strains. (PDF 232 kb) [file 12864_2019_5680_MOESM9_ESM.pdf]

Intraspecific variation in *Trichoderma harzianum* as estimated based on the analysis of the two strains

Contents

Th_T 1

Th 5

Having two isolates from the same species (*T. harzianum*) available, we were interested in the possible differences in their genomes. A comparison showed that 1 699 genes of strain T. *harzianum* CBS 226.95 (type - Th_T) (12 %) were absent from strain *T. harzianum* (Th), and 1 419 genes of the latter (10.1 %) absent from Th_T. Most of these genes encoded orphan proteins, and a function could only be predicted for 158 and 160 genes in Th_T and Th, respectively (Table 1). In both cases, proteins with ankyrin repeats (34 vs. 23, respectively) accounted for the highest number. Besides this, the composition of the two gene pools was different: Th had 11 unique Zn_2Cys_6 transcriptional regulators, whereas Th_T only three. On the other hand, the unique genes of the latter contained three genes encoding proteins of the Dimer_Tnp_hAT (zinc fingers/transposases) and six genes encoding EEP-superfamily AP-endonucleases, which were absent from Th.

Table 1: Orphan genes in individual strains of *T. harzianum*

Protein ID	E-Value	Bitscore	Accession	Short name
Th_T				
5	3,31E-43	173.233	cl12078	p450 superfamily
505941	3,64E-136	448.046	cd05930	A_NRPS
509829	9,75E-118	367.607	cd13970	ABC1_ADCK3
479909	3,62E-07	618.295	cl21494	Abhydrolase superfamily
493457	4,03E-13	756.242	pfam00583	Acetyltransf_1
555787	7,51E-08	614.079	cl08282	Acyl_transf_1 superfamily
502041	2,43E-33	130.82	cd01428	ADK
76331	4,49E-44	156.311	cl17173	AdoMet_MTases superfamily
7387	6,70E-27	116.06	cl26705	AIR1 superfamily
55675	1,59E-23	104.504	cl26705	AIR1 superfamily
68770	4,37E-39	139.826	cd00204	ANK
63203	4,55E-31	131.737	cd00204	ANK
96791	9,99E-31	119.796	cd00204	ANK
16486	2,70E-29	127.5	cd00204	ANK
551129	1,97E-27	118.255	cd00204	ANK
66970	2,95E-25	104.773	cd00204	ANK

44203	5,52E-25	103.232	cd00204	ANK
39922	6,05E-24	997.654	cd00204	ANK
95090	1,74E-23	110.936	cd00204	ANK
91098	5,13E-23	100.151	cd00204	ANK
485886	1,03E-22	108.625	cd00204	ANK
66778	1,13E-22	943.726	cd00204	ANK
97129	1,20E-22	95.143	cd00204	ANK
74810	1,25E-20	916.762	cd00204	ANK
42246	1,30E-20	920.614	cd00204	ANK
76492	3,20E-20	928.318	cd00204	ANK
546880	7,97E-18	924.466	cd00204	ANK
36422	3,09E-17	828.166	cd00204	ANK
554495	2,48E-16	89.365	cd00204	ANK
96787	3,14E-16	89.365	cd00204	ANK
335626	8,18E-16	882.094	cd00204	ANK
53553	5,08E-13	708.754	cd00204	ANK
62568	2,56E-12	677.938	cd00204	ANK
513177	6,21E-12	754.978	cd00204	ANK
486578	8,93E-11	674.086	cd00204	ANK
75602	1,61E-10	647.122	cd00204	ANK
44411	2,80E-10	64.327	cd00204	ANK
505651	3,69E-10	616.306	cd00204	ANK
62242	9,26E-10	631.714	cd00204	ANK
43331	1,34E-08	597.046	cd00204	ANK
46571	2,95E-08	589.342	cd00204	ANK
46902	3,43E-06	535.414	cd00204	ANK
504418	2,52E-05	52.771	cd00204	ANK
307354	3,25E-11	769.871	cl26073	Ank_2 superfamily
397141	0	551.021	pfam10351	Apt1
514125	9,89E-47	166.662	COG0656	ARA1
181629	1,70E-94	283.307	pfam05856	ARPC4
506612	5,10E-15	718.095	pfam04627	ATP-synt_Eps
510620	4,17E-18	792.094	pfam04911	ATP-synt_J
75711	7,18E-08	627.374	cd14688	bZIP_YAP
2327	5,29E-104	312.2	smart01085	CK_II_beta
509278	7,64E-09	586.637	pfam14420	Clr5
81754	7,55E-145	440.825	pfam08318	COG4
488820	7,54E-28	119.585	cl09938	cond_enzymes superfamily
3574	2,00E-09	602.692	pfam05051	COX17
9653	1,20E-20	983.257	cl25401	DEXDc superfamily
102899	6,91E-12	649.737	pfam05699	Dimer_Tnp_hAT zin finger/transposase
102332	5,52E-06	522.621	pfam05699	Dimer_Tnp_hAT zin finger/transposase
102764	8,06E-06	514.917	pfam05699	Dimer_Tnp_hAT zin finger/transposase
102891	1,53E-05	518.769	pfam05699	Dimer_Tnp_hAT zin finger/transposase

3404	1,75E-88	290.686	cd08771	DLP_1
2723	5,15E-117	392.402	COG1112	DNA2
503495	9,17E-39	138.349	pfam06993	DUF1304
10467	1,14E-16	875.008	cl13276	DUF3328 superfamily
86546	1,78E-65	224.727	cl16553	DUF4246 superfamily
496025	1,15E-88	271.331	pfam14124	DUF4291
81722	8,20E-21	917.857	pfam14232	DUF4334
7246	2,40E-23	104.726	cl26026	ECR1_N superfamily
61981	1,53E-08	585.362	cl00490	EEP superfamily, AP-endonuclease
65765	1,53E-08	585.362	cl00490	EEP superfamily, AP-endonuclease
65915	1,53E-08	585.362	cl00490	EEP superfamily, AP-endonuclease
66670	1,53E-08	585.362	cl00490	EEP superfamily, AP-endonuclease
70599	1,53E-08	585.362	cl00490	EEP superfamily, AP-endonuclease
39707	1,40E-07	585.362	cl00490	EEP superfamily, AP-endonuclease
488543	1,28E-28	131.028	cd12148	fungal_TF_MHR
98690	3,25E-05	628.472	cd12148	fungal_TF_MHR
440598	6,27E-05	531.326	smart00066	GAL4
509533	1,05E-43	158.09	cd03139	GATase1_PfpI_2
476765	2,84E-07	61.984	pfam01465	GRIP
517912	2,33E-11	664.823	cl02776	GST_C_family superfamily
524580	4,63E-69	237.107	pfam00632	HECT
502100	1,91E-38	146.089	cl00275	Heme_Cu_Oxidase_I superfamily
118924	3,28E-13	816.359	cl06163	HET superfamily
539240	2,06E-11	720.059	cl06163	HET superfamily
268489	8,24E-05	487.698	cl09264	HTH_Tnp_Tc3_2 superfamily
493416	3,11E-09	610.842	pfam06766	Hydrophobin_2
517965	2,80E-78	259.414	pfam08659	KR
419367	1,61E-58	208.953	pfam08659	KR
454456	7,00E-52	192.389	pfam08659	KR
267637	7,65E-92	278.922	cd03357	LbH_MAT_GAT
430787	2,37E-07	615.952	cl01378	LicD superfamily
40729	3,92E-09	594.348	pfam00538	Linker_histone
259757	1,48E-29	136.005	cl25662	Lys2b superfamily
104176	1,60E-30	120.826	cd01389	MATA_HMG-box
508975	1,74E-28	129.356	cd06174	MFS
312954	3,64E-05	577.089	cd06174	MFS
493691	1,33E-12	761.559	pfam00153	Mito_carr
498069	1,69E-18	965.318	cl28162	Mito_carr superfamily
201421	0	623.069	cd07415	MPP_PP2A_PP4_PP6
282	4,94E-06	579.708	cl13995	MPP_superfamily superfamily
383010	6,85E-105	328.029	cl27221	mRNA_cap_C superfamily
11856	0	1115.06	cl25937	MT superfamily
223156	2,70E-12	741.938	cl05445	Mt_ATP-synt_D superfamily
543016	5,33E-45	162.037	cl25327	NACHT_N superfamily

513767	9,86E-31	126.135	cl21454	NADB_Rossmann superfamily
142220	1,18E-69	245.991	cl27296	NarK superfamily
519762	4,12E-127	377.598	cd07564	nitrilases_CHs
146347	4,87E-33	128.715	pfam04248	NTP_transf_9
490220	5,79E-40	164.374	cl12078	p450 superfamily
3182	2,52E-34	145.315	cl26005	PLN03088 superfamily
19562	1,78E-16	875.465	cl21455	P-loop_NTPase superfamily
133075	1,13E-12	843.419	cl21455	P-loop_NTPase superfamily
95721	8,49E-07	646.967	cl21455	P-loop_NTPase superfamily
508167	4,30E-06	512.296	pfam01679	Pmp3
513372	2,96E-53	180.001	cl25685	PNPOx_C superfamily
549944	7,59E-59	213.4	cl27709	Proton_antipo_M superfamily
102912	1,02E-12	802.501	cl27709	Proton_antipo_M superfamily
477096	5,45E-62	200.866	cd03013	PRX5_like
502098	2,03E-18	902.264	cl27766	QcrB superfamily
553234	2,88E-15	826.416	cl27766	QcrB superfamily
95382	3,52E-49	178.546	cl28885	RecA-like_NTPases superfamily
508360	3,09E-18	988.938	cl20311	Ribophorin_II superfamily
138179	2,98E-71	225.307	cd01417	Ribosomal_L19e_E
493064	6,99E-09	552.672	pfam00832	Ribosomal_L39
514402	6,62E-16	822.455	cl14782	RNase_H_like superfamily
248881	3,57E-05	510.645	cl14782	RNase_H_like superfamily
74909	2,52E-07	572.709	COG0333	RpmF
367278	7,73E-25	114.311	cd01650	RT_nLTR_like
91095	3,65E-59	207.077	cl25409	SDR superfamily
482875	6,08E-53	192.987	cl25409	SDR superfamily
488648	6,63E-20	922.874	cl25409	SDR superfamily
486613	1,56E-11	711.508	cl25409	SDR superfamily
411621	2,93E-67	223.547	pfam06775	Seipin
368002	2,00E-11	751.236	COG1676	SEN2
231582	3,23E-111	382.14	COG1196	Smc
486332	5,04E-18	979.385	COG0513	SrmB
80799	1,29E-71	237.513	COG0009	SUA5
2876	3,69E-99	308.793	pfam09728	Taxilin
6440	1,21E-14	848.381	pfam12009	Telomerase_RBD
522572	1,74E-76	246.731	pfam00314	Thaumatin
476434	7,46E-13	719.938	cl00388	Thioredoxin_like superfamily
420653	1,26E-53	182.962	cd07758	ThTPase
509742	8,44E-10	631.148	TIGR04076	TIGR04076
7871	4,75E-08	556.814	pfam10642	Tom5
510039	1,34E-14	72.134	pfam08038	Tom7
125203	4,29E-90	279.789	pfam02676	TYW3
478617	1,39E-28	120.037	pfam03981	Ubiq_cyt_C_chap
551846	2,21E-38	157.113	cl27298	UhpC superfamily

83733	4,77E-24	984.429	pfam05254	UPF0203
68176	7,86E-19	906.735	cl22977	UvrD_C_2 superfamily
100842	5,79E-35	137.852	cl25539	WD40 superfamily
485860	0	800.645	cl28315	XylB superfamily
129913	1,82E-12	723.391	cl10015	YjgF_YER057c_UK114_family superfamily
488545	3,16E-10	754.983	COG3386	YvrE
508791	5,54E-08	619.408	pfam01428	zf-AN1
7976	2,86E-19	950.101	pfam00645	zf-PARP
488615	9,44E-29	120.907	cl00064	ZnMc superfamily
509677	4,62E-19	87.352	cl00064	ZnMc superfamily
Th				
1819495	3,64E-78	252.548	cd05339	17beta-HSDXI-like_SDR_c
1819342	4,64E-17	947.256	cl28897	7tm_GPCRs superfamily
1819618	0	963.614	PHA00002	A
181852	6,53E-09	631.336	cl21494	Abhydrolase superfamily
1819103	2,98E-125	403.541	pfam01602	Adaptin_N
1819328	1,64E-132	407.635	pfam03155	Alg6_Alg8
1823859	1,79E-05	501.077	pfam10471	ANAPC_CDC26
1720709	1,59E-30	119.025	cd00204	ANK
1719114	3,88E-24	997.654	cd00204	ANK
1683779	1,06E-22	974.542	cd00204	ANK
1668933	8,44E-22	947.578	cd00204	ANK
1655210	4,48E-20	909.058	cd00204	ANK
1735942	8,69E-20	897.502	cd00204	ANK
1658401	1,22E-19	909.058	cd00204	ANK
1682284	2,22E-18	858.982	cd00204	ANK
1659581	4,63E-18	866.686	cd00204	ANK
1655576	7,48E-18	843.574	cd00204	ANK
1675034	1,79E-17	83.587	cd00204	ANK
1679631	3,87E-16	801.202	cd00204	ANK
1707891	4,27E-16	79.735	cd00204	ANK
1707907	1,47E-15	793.498	cd00204	ANK
1676974	5,43E-15	793.498	cd00204	ANK
1626541	1,53E-14	75.883	cd00204	ANK
1670470	2,74E-14	743.422	cd00204	ANK
1659605	5,08E-13	708.754	cd00204	ANK
1657030	1,86E-12	708.754	cd00204	ANK
1687250	8,29E-11	658.678	cd00204	ANK
1676697	4,22E-10	635.566	cd00204	ANK
1658056	7,69E-10	631.714	cd00204	ANK
1652605	1,33E-09	650.974	cd00204	ANK
1819272	4,36E-155	453.096	pfam03452	Anp1

1819362	2,19E-127	387.687	cd01085	APP
1819127	0	587.837	cl00279	APP_MetAP superfamily
1819118	1,26E-127	380.773	TIGR01850	argC
1871608	3,73E-91	288.816	cd06097	Aspergillopepsin_like
1833612	1,29E-44	171.62	pfam10377	ATG11
1656780	5,17E-17	788.242	pfam04911	ATP-synt_J
1819122	1,41E-90	298.984	COG2303	BetA
1651838	5,85E-07	594.374	pfam00144	Beta-lactamase
1819327	3,69E-160	471.89	cd09245	BRO1_UmRIM23-like
1712215	1,20E-10	692.568	smart00225	BTB
1819159	6,16E-21	108.11	cl25570	Cation_efflux superfamily
1786288	4,62E-15	779.999	pfam03879	Cgr1
1819322	9,21E-31	129.968	pfam13838	Clathrin_H_link
1819084	4,69E-69	273.083	cl26330	COG5219 superfamily
1819120	0	822.708	cl26699	COP-gamma_platf superfamily
167062	2,81E-13	698.991	pfam05051	COX17
1819292	0	696.216	cl27876	Cse1 superfamily
1819326	9,59E-77	260.083	COG2072	CzcO
1787236	3,83E-102	301.471	PHA00006	D
1819410	4,50E-11	725.191	pfam08590	DUF1771
1838481	1,73E-05	52.478	pfam08648	DUF1777
1542378	9,57E-42	159.742	cl25578	DUF2855 superfamily
1671350	2,34E-31	129.774	cl25843	DUF3295 superfamily
1684390	3,87E-20	93.664	cl13276	DUF3328 superfamily
1797598	7,01E-05	555.216	cl13450	DUF3505 superfamily
1819096	0	580.71	pfam12054	DUF3535
1853881	4,38E-36	140.674	pfam06441	EHN
1701784	2,31E-10	684.963	cl27552	FAD_binding_3 superfamily
1819282	3,19E-50	196.512	cd12148	fungal_TF_MHR
1819407	3,29E-20	108.686	cd12148	fungal_TF_MHR
1819125	7,88E-20	107.53	cd12148	fungal_TF_MHR
1819216	9,62E-20	107.145	cd12148	fungal_TF_MHR
1819285	2,48E-19	104.834	cd12148	fungal_TF_MHR
1706949	1,01E-05	539.973	smart00906	Fungal_trans
1369275	6,51E-35	148.719	pfam11951	Fungal_trans_2
1767469	3,38E-08	639.182	smart00066	GAL4
130995	1,30E-07	61.607	smart00066	GAL4
1258521	7,91E-05	515.918	smart00066	GAL4
1819235	8,83E-11	704.249	pfam00320	GATA
1720020	2,81E-117	356.507	cd08990	GH43_AXH_like
1819325	4,91E-99	313.356	cd09001	GH43_XYL_2
1819466	1,46E-11	818.648	COG0277	GlcD
1722194	3,39E-18	908.121	cl14813	GluZincin superfamily
1819194	3,89E-121	375.711	pfam03659	Glyco_hydro_71

1854309	1,09E-39	160.239	cl02091	Glyco_transf_15 superfamily
1819476	6,64E-34	132.733	cl02130	Got1 superfamily
1819279	4,01E-27	129.735	cl25775	GppA superfamily
1274609	1,76E-25	100.695	pfam04568	IATP
1819179	0	816.381	pfam06437	ISN1
1819317	3,19E-82	277.508	cl04955	LanC_like superfamily
1783638	5,10E-22	964.139	pfam00538	Linker_histone
1788216	5,59E-06	624.353	cl00013	Lyase_I_like superfamily
1819124	0	539.905	COG0833	LysP
1863060	5,59E-28	121.49	pfam17043	MAT1-1-2
1863056	5,27E-27	108.115	cd01389	MATA_HMG-box
1434806	1,43E-62	211.613	pfam04769	MATalpha_HMGbox
1828769	0	2163.59	cl26761	MDN1 superfamily
1848906	1,16E-07	65.283	cl16912	MDR superfamily
1819452	2,24E-28	120.514	cl10917	Med8 superfamily
1808158	1,69E-20	957.104	pfam13649	Methyltransf_25
1787235	0	576.039	pfam04687	Microvir_H
1819471	1,97E-21	103.505	pfam00153	Mito_carr
1819375	0	566.969	cd07414	MPP_PP1_PPKL
1819168	5,30E-61	219.478	cl13995	MPP_superfamily superfamily
1692229	2,39E-19	897.798	cl21454	NADB_Rossmann superfamily
1819459	8,44E-23	116.247	cl20384	NatB_MDM20 superfamily
1819214	0	529.901	cl17037	NBD_sugar-kinase_HSP70_actin superfamily
1819365	2,32E-15	913.288	cl17037	NBD_sugar-kinase_HSP70_actin superfamily
1819391	3,12E-37	148.734	pfam05224	NDT80_PhoG
1819356	9,06E-58	204.878	cl17190	NK superfamily
1819145	1,23E-100	315.364	pfam04981	NMD3
1819313	3,59E-23	123.549	cl24232	Nup188 superfamily
1819218	0	680.057	cl14607	OPT superfamily
1866754	1,20E-06	579.597	cl14607	OPT superfamily
1707536	7,49E-08	547.884	pfam10215	Ost4
1819510	1,10E-53	200.968	cl12078	p450 superfamily
1819163	3,35E-54	194.121	COG3491	PcbC
1819490	3,62E-20	103.599	COG3491	PcbC
1819224	1,09E-105	343.432	cd07489	Peptidases_S8_5
1778820	9,98E-14	790.719	pfam04695	Pex14_N
1787237	0	730.511	cl15846	Phage_F superfamily
1787234	7,47E-81	244.646	cl11610	Phage_G superfamily
1819465	0	640.477	cl27322	PheS superfamily
1819415	4,97E-26	115.51	cl17171	PH-like superfamily
1819397	1,78E-28	127.387	cd00180	PKc
1819161	2,24E-23	112.705	cl21453	PKc_like superfamily
1819436	6,25E-118	389.223	pfam08429	PLU-1
1819139	2,34E-14	760.296	pfam07491	PPI_Ypi1

1819301	1,35E-10	714.314	pfam07915	PRKCSH
1819109	5,00E-128	371.577	cd03761	proteasome_beta_type_5
1819182	3,62E-70	243.324	pfam08324	PUL
1819428	1,42E-78	274.201	pfam10257	RAI16-like
1799442	3,55E-24	979.758	pfam01781	Ribosomal_L38e
1786425	6,99E-09	552.672	pfam00832	Ribosomal_L39
1752888	8,92E-20	896.883	cd05831	Ribosomal_P1
1786896	2,18E-28	111.586	cd05833	Ribosomal_P2
1836906	1,43E-07	558.235	smart00659	RPOLCX
1819255	1,68E-132	429.533	cl25971	RSN1_7TM superfamily
1809165	1,93E-05	534.233	cl21549	rve superfamily
1785912	3,24E-24	977.059	cd04457	S1_S28E
1785916	3,54E-22	107.31	cl25409	SDR superfamily
1587482	1,74E-15	827.036	cl25409	SDR superfamily
1819447	1,30E-49	177.091	cd05233	SDR_c
1819469	1,04E-12	820.084	cl21737	Sds3 superfamily
1819440	6,17E-125	392.602	cl27768	SelB superfamily
1867908	3,73E-09	647.685	cl27768	SelB superfamily
1819354	1,75E-39	156.341	smart00317	SET
1819149	0	584.37	COG1196	Smc
1805959	5,85E-06	643.187	cl25732	SMC_N superfamily
1819104	4,86E-24	109.684	cl28099	SmtA superfamily
1819277	0	1121.81	cl26465	SNF2_N superfamily
1819226	6,02E-84	276.066	pfam04086	SRP-alpha_N
1819281	2,21E-158	473.174	cd07830	STKc_MAK_like
1819223	1,94E-80	272.608	pfam00083	Sugar_tr
1819251	0	574.115	cl26602	SunT superfamily
1819258	3,26E-101	314.415	cd00757	ThiF_MoeB_HesA_family
1853856	7,01E-21	962.614	cl00388	Thioredoxin_like superfamily
1719589	1,22E-11	679.725	cl00388	Thioredoxin_like superfamily
1784854	1,29E-05	532.562	pfam03647	Tmemb_14
1809815	4,75E-08	556.814	pfam10642	Tom5
1811287	1,34E-14	72.134	pfam08038	Tom7
1819175	0	542.824	cl27547	TruB_N superfamily
1779516	1,66E-06	544.367	cd14361	UBA_HYPK
1819230	5,52E-33	133.822	cl00155	UBQ superfamily
1819135	5,36E-43	172.961	pfam00443	UCH
1819268	2,55E-33	144.402	cl27298	UhpC superfamily
1819080	7,43E-17	915.033	pfam02204	VPS9
1584519	7,89E-40	142.445	cl10015	YjgF_YER057c_UK114_family superfamily
1668876	1,17E-06	535.288	pfam13917	zf-CCHC_3
