# Supplementary material for: Evolution and comparative genomics of the most common Trichoderma species
Source: BMC Genomics. 2019 Jun 12;20:485. doi: 10.1186/s12864-019-5680-7 (PMC6560777; doi:10.1186/s12864-019-5680-7)
Supplement: Supplementary file 10 — Unique genes shared only between facultative pathogenic Trichoderma species. (PDF 165 kb) [file 12864_2019_5680_MOESM10_ESM.pdf]

Unique genes shared only between facultative pathogenic *Trichoderma* species

We were also interested whether the two species that are most frequently identified as opportunistic pathogens of immunocompromised (and rarely immunocompetent) humans, – *T. longibrachiatum* and *T. citrinoviride* (Kuhls *et al.*, 1999) – would contain unique genes. Reciprocal BLASTP identified 94 genes that were only present in *T. citrinoviride* and *T. longibrachiatum*, and not present in any other *Trichoderma* spp. We could identify putative functions for 53 of them (Table 1). Besides ankyrin-containing proteins and Zn_2Cys_6 transcriptional regulators, we detected two genes that encoded regulators of the heat shock response (Trilo3: 1397814 and Trilo3: 1337649), and which could theoretically play a role during growth of these two species at temperatures of the human body. In addition, two genes encoding proteins with CFEM (common in fungal extracellular membranes; (Kulkarni *et al.*, 2003)) domains (Trilo3:38581 and Trilo3:4108), and three genes that encode unknown proteins with a single transmembrane domain (Trilo3:583, Trilo3:1187332 and Trilo3:1398588) could play a role in signaling the presence of the animal host. Because of the incompleteness of the *T. longibrachiatum* genome there may be additional genes not detected. Yet, the results obtained open the possibility to investigate *Trichoderma* pathogenicity on humans at the gene level.

Table 1. Unique genes shared by *T. longibrachiatum* and *T. citrinoviride*

Tlong3.0	E-Value	Bitscore	Accession	Short name	location	TMs*
4108	7,96E-03	482.696	cl02770	CFEM superfamily	secreted	1 TM o
5483				ND**		1 TM i
18283	1,41E-08	622.669	pfam09135	Alb1		
22351	1,75E-02	457.235	cl28542	BUR6 superfamily		
38581	7,50E+00	366.498	pfam05730	CFEM	secreted	
48520	1,34E-07	58.49	pfam10235	Cript		
299214				ND**		2TM i
345516	2,12E-31	128.082	cl00224	PLPDE_IV superfamily		
362931	8,53E+00	435.598	cl23766	Zn2Cys6 transcriptional regulator		
1040237	0.00757436	362.919	cl26247	DNA_pol3_delta2 superfamily		
1187332				ND**		1 TM o
1296664	2,73E-15	774.238	cd00204	ANK		
1304722	3,02E-31	120.566	cd00204	ANK		
1321854	4,76E-22	105.745	cd00306	Peptidases_S8_S53		
1323003	3,85E-25	102.621	pfam04568	IATP		
1328464	1,68E-25	104.773	cd00204	ANK		
1331520	6,21E-17	796.646	pfam11034	Grg1		

1333087	3,06E-50	181.628	cl16912	MDR superfamily		
1337649	5,70E-09	594.673	pfam06825	HSBP1		
1338738	4,86E+00	446.121	cd06174	MFS		8TM i
1339497	1,14E-13	797.068	pfam11905	DUF3425		
1342261	8,51E-03	547.581	cd12148	Zn2Cys6 transcriptional regulator		
1344902	1,27E-19	903.629	cl14632	VOC superfamily		
1347255	5,29E-04	515.918	smart00066	Zn2Cys6 transcriptional regulator		
1367383	9,96E-01	420.845	pfam12937	F-box-like		
1375601	5,26E-09	606.562	pfam05347	Complex1_LYR		
1376707	4,11E-10	705.306	pfam13847	Methyltransf_31		
1378765	3,21E-01	477.048	cl00459	MIT_CorA-like superfamily		2TM o
1390335	5,38E-06	553.099	cl21453	PKc_like superfamily		
1395561	1,65E-21	999.159	cl16912	MDR superfamily		
1397290	3,24E-24	977.059	cd04457	S1_S28E		
1397814	2,91E-05	558.038	cd14688	bZIP_YAP		
1398588				ND**		1TM o
1398848	8,23E-03	492.654	cd06661	GGCT_like		
1399326	2,45E-20	875.942	pfam05365	UCR_UQCRX_QCR9		
1401724	1,22E-32	126.098	pfam08651	DASH_Duo1		
1404215	2,15E-08	638.233	cl02518	BTB superfamily		
1418460	8,73E-123	363.447	PRK08259	PRK08259		
1424428				ND**	secreted	
1426776	4,95E-03	470.491	cl01553	GFA superfamily		
1431211	3,04E-30	128.207	COG0596	MhpC		
1437539				ND**	secreted	
1439387				ND**		2TM i
1440742				ND**	secreted	
1448718	6,66E-01	432.029	cl13276	DUF3328 superfamily		
1461835	1,13E-46	160.53	pfam14832	Tautomerase_3		
1463224	1,39E-17	923.659	cl25409	SDR superfamily		
1464596	9,77E-15	822.849	cl21454	NADB_Rossmann superfamily		
1466625	1,07E-22	942.688	pfam08991	MTCP1		
1473118	1,64E-64	214.321	cl27753	adh_short superfamily		

* TM, transmembrane helices; o and i point to the location of the N-terminus (outside or inside the cell, respectively;

**ND, no conserved domain detected

References:

Kuhls K, Lieckfeldt E, Borner T & Gueho E (1999) Molecular reidentification of human pathogenic *Trichoderma* isolates as *Trichoderma longibrachiatum* and *Trichoderma citrinoviride*. *Med Mycol* **37**: 25-33.

Kulkarni RD, Kelkar HS & Dean RA (2003) An eight-cysteine-containing CFEM domain unique to a group of fungal membrane proteins. *Trends Biochem Sci* **28**: 118-121.
