# Supplementary material for: Evolution and comparative genomics of the most common Trichoderma species
Source: BMC Genomics. 2019 Jun 12;20:485. doi: 10.1186/s12864-019-5680-7 (PMC6560777; doi:10.1186/s12864-019-5680-7)
Supplement: Supplementary file 12 — Trichoderma genes involved in sensing of the mating partner. (PDF 285 kb) [file 12864_2019_5680_MOESM12_ESM.pdf]

Trichoderma genes involved in sensing of the mating partner

Sensing of a potential mating partner is a prerequisite for sexual reproduction, and is fulfilled by a pheromone system consisting of peptide pheromone precursors and pheromone receptors (Nieuwenhuis & Aanen, 2012). Genome analysis shows that all 12 *Trichoderma* spp. have genes encoding the α-type peptide pheromone precursor PPG1, which is comparable to its homolog in other fungi.

	T. reesei	*T. longibrachiatum*	*T. citrinoviride*	*T. parareesei*	*T. harzianum*	*T. guizhouense*	*T. afroharzianum*	*T. virens*	*T. atroviride*	*T. gamsii*	*T. asperellum*	*T. hamatum*
PPG1, α type	104292	317925	1198762	OTA03422	86570	OPB47026	1241	44505	297240	18666200	144740	4957
HPP1	34493	1420161	1128304	*	463451	OPB39925	830	91991	*	*	*	*
STE2 = HPR2	64018	29766	1115433	OTA07042	96494	OPB41683	4616	21417	17079, 36032	18661910	44057	7747
STE3 = HPR1	57526	1322746	1207301	OTA04631	107686	OPB39349	6116	39157	147894	18660796	127205	1425

* no annotation, gene presence confirmed by translation of nt sequences

In contrast, *Trichoderma* does not contain orthologs of the **a**-type peptide pheromone precursors, but instead has h-type precursors (HPP1), which share characteristics of **a**- and α-types by containing the consensus motif (LI)GC(TS)VM (Schmoll *et al.*, 2010). Interestingly, the HPP1 orthologs of members of ST are shorter because they contain only 1 or 2 copies of the consensus motif: In addition, all *Trichoderma* spp. contain the respective peptide pheromone precursors bind to pheromone receptors HPR1 (*ste3*) and HPR2 (*ste2*), which typical for heterothallic ascomycetes, all *Trichoderma* spp. have one MAT1-1 associated STE2p-type peptide pheromone receptor (HPR2), and one MAT1-2 associated STE3p-type peptide pheromone receptor (HPTR1).

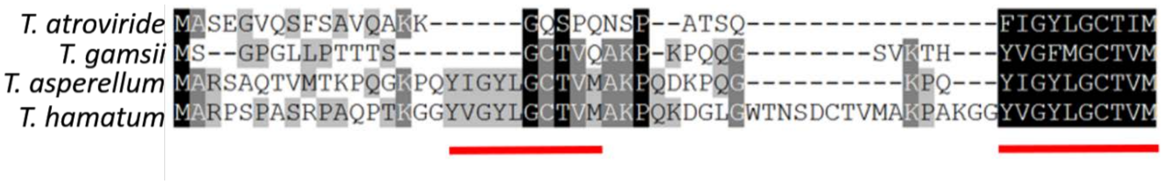

Sequence of the HPP1 proteins from *Trichoderma* spp. of ST. The conserved YVGYLGCTVM motif is underlined.

References

Nieuwenhuis BPS & Aanen DK (2012) Sexual selection in fungi. *J Evolution Biol* **25**: 2397-2411.
Schmoll M, Seibel C, Tisch D, Dorrer M & Kubicek CP (2010) A novel class of peptide pheromone precursors in ascomycetous fungi. *Mol Microbiol* **77**: 1483-1501.
