# Supplementary material for: Evolution and comparative genomics of the most common Trichoderma species
Source: BMC Genomics. 2019 Jun 12;20:485. doi: 10.1186/s12864-019-5680-7 (PMC6560777; doi:10.1186/s12864-019-5680-7)
Supplement: Supplementary file 13 — Carbon metabolism in Trichoderma. (PDF 224 kb) [file 12864_2019_5680_MOESM13_ESM.pdf]

Carbon metabolism in *Trichoderma*

Carbon metabolism of *Trichoderma* has so far been studied only in *T. reesei* and with respect to the catabolism of hemicellulose and pectin monomers (Seiboth & Metz, 2011, Mojzita *et al.*, 2012, Druzhinina & Kubicek, 2016). Although the core genome encodes several alcohol dehydrogenases with high similarity to ethanol dehydrogenases, previous studies have shown that the catabolism of D-glucose is strongly dependent on aerobic conditions (Bonaccorsi *et al.*, 2006, Jouhten *et al.*, 2009). We detected several copies of some of the enzymes involved in glycolysis (hexokinases and enzymes acting at the triose phosphate stage) and the pentose phosphate pathway (transaldolases and transketolases) suggesting strong glycolytic and pentose catabolic and interchanging activities. Interestingly, we also found a glucose oxidase (GOD), a gluconolactonase, and two gluconate kinases indicating the presence of a second oxidative D-glucose catabolizing pathway (**Additional File 9**). The presence of an extracellular GOD in *Trichoderma* was unexpected, because the GOD of *Aspergillus niger* was previously used as a reporter for gene expression in *T. atroviride* and no GOD activity was found in this species (Mach *et al.*, 1999). This can be explained by the fact that a GOD orthologue is indeed absent from species of ST, but present in SL and HV:

Strain	ID**	E-value	identity**
T. reesei	22915	0.0	1
T. parareesei	OTA01046.1	0.0	0.96
T. virens	46709	0.0	0.7
T. afroharzianum	KKP00902.1	0.0	0.68
T. guizhouense	OPB44891.1	0.0	0.67
T. harzianum	93671	0.0	0.68
T. longibrachiatum	1441805	0.0	0.94
T. citrinoviride	1143940	0.0	0.92
T. atroviride	best hit	1,2xE-34	0.41
T. asperellum	best hit	1,0xE-32	0.4
T. hamatum	best hit	4,3xE-32	0.38
T. gamsii	best hit	2,5xE-33	0.4

* Trire2:22915 was identified as GOD orthologue by BLASTP using the GOD of *A. niger* (EHA23687.1) as a bait (5.1E-112, 89.4 % coverage; 43.4 % identity); the *T. reesei* GOD was then used to blast the other species

* JGI or NCBI identifiers; best hits are shown for those cases where the presence of a GOD ortholog is rejected

***at amino acid level

Another interesting finding was the detection of two D-xylulose-5-phosphate/D-fructose-6-phosphate ketolases (XFPK) in all *Trichoderma* spp. Originally believed to occur only in lactic acid bacteria, XFPKs have also been detected in yeast (Glenn *et al.*, 2014), and offer an additional pathway for catabolism of pentoses.

We also detected the presence of two D-arabinose dehydrogenases and a D-arabino-1,4-lactone oxidase in *Trichoderma*. These enzymes are involved in the biosynthesis of D-erythroascorbic acid, an equivalent of plant ascorbic acid and which protects against oxidative stress (Spickett *et al.*, 2000)).

Finally, we detected the genes encoding the enzymes for glutathione-linked methanol degradation (see below). This pathway has been demonstrated in the methylotrophic yeast *Candida boidinii* (Yurimoto *et al*., 2003), but not yet in filamentous ascomycetes.

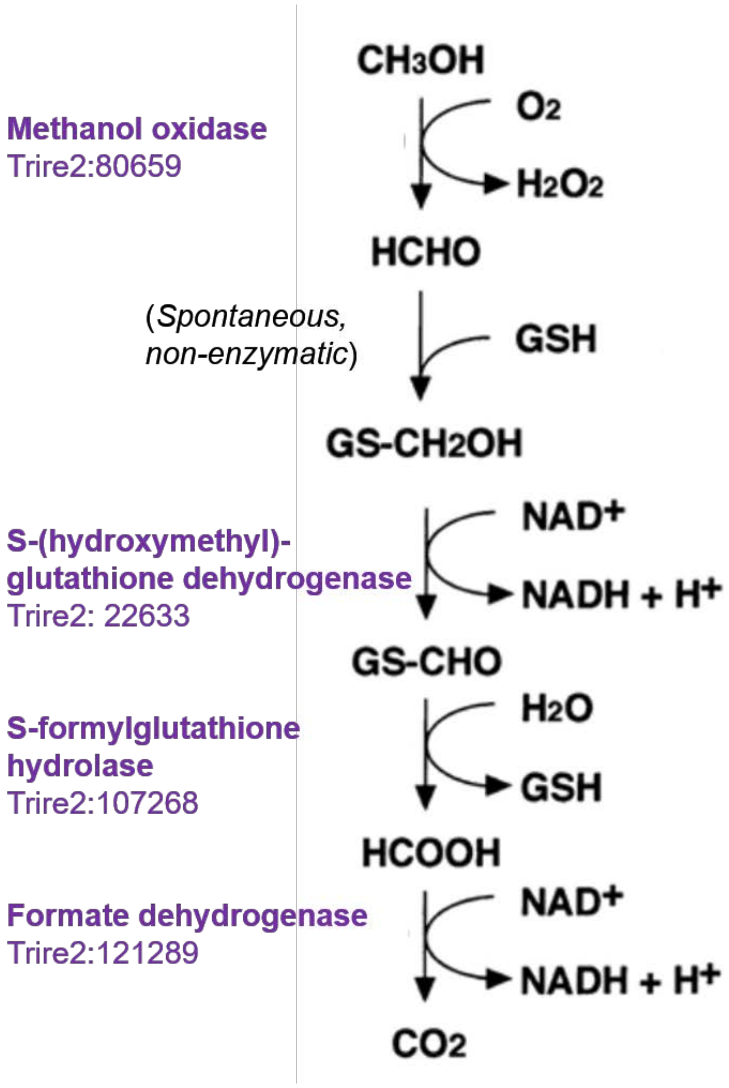

Presence of the enzymes required for methanol catabolism to carbon dioxide in the *Trichoderma* pan genome. Only *T. reesei* IDs are given but those for the orthologs in other species can be obtained from additional file 9.

Methanol is in nature formed during the defense of plants against pathogens (Lionetti *et al.*, 2017) and during the depolymerization of pectin and lignin (see (Kubicek, 2012). *T.„lignorum“* (this species name is illegimate, and the correct species identity of the isolate is unknown) has been reported to be capable of growing on methanol (Tye & Willetts, 1977), but the enzymes involved were not investigated. The presence of a methanol oxidase has been demonstrated in white rot and brown rot basidiomycetes and discussed as a mechanism for production of hydrogen peroxide required for lignin peroxidases or Fenton chemistry (Ferreira *et al.*, 2015).

References

Bonaccorsi ED, Ferreira AJS, Chambergo FS, Ramos ASP, Mantovani MC, Farah JPS, Sorio CS, Gombert AK, Tonso A & El-Dorry H (2006) Transcriptional response of the obligatory aerobe *Trichoderma*

reesei to hypoxia and transient anoxia: Implications for energy production and survival in the absence of oxygen. *Biochemistry-Us* **45**: 3912-3924.
Druzhinina IS & Kubicek CP (2016) Familiar Stranger: Ecological Genomics of the Model Saprotroph and Industrial Enzyme Producer *Trichoderma reesei* Breaks the Stereotypes. *Adv Appl Microbiol* **95**: 69-147.
Ferreira P, Carro J, Serrano A & Martinez AT (2015) A survey of genes encoding H2O2-producing GMC oxidoreductases in 10 Polyporales genomes. *Mycologia* **107**: 1105-1119.
Glenn K, Ingram-Smith C & Smith KS (2014) Biochemical and Kinetic Characterization of Xylulose 5-Phosphate/Fructose 6-Phosphate Phosphoketolase 2 (Xfp2) from Cryptococcus neoformans. *Eukaryotic Cell* **13**: 657-663.
Jouhten P, Pitkanen E, Pakula T, Saloheimo M, Penttila M & Maaheimo H (2009) C-13-metabolic flux ratio and novel carbon path analyses confirmed that *Trichoderma reesei* uses primarily the respirative pathway also on the preferred carbon source glucose. *Bmc Syst Biol* **3**.
Kubicek CP (2012) *Fungi and Lignocellulosic Biomass*. John Wiley & Sons, Inc.
Lionetti V, Fabri E, De Caroli M, Hansen AR, Willats WGT, Piro G & Bellincampi D (2017) Three Pectin Methylesterase Inhibitors Protect Cell Wall Integrity for *Arabidopsis* Immunity to *Botrytis*. *Plant Physiol* **173**: 1844-1863.
Mach RL, Peterbauer CK, Payer K, Jaksits S, Woo SL, Zeilinger S, Kullnig CM, Lorito M & Kubicek CP (1999) Expression of two major chitinase genes of *Trichoderma atroviride* (T-harzianum P1) is triggered by different regulatory signals. *Appl Environ Microbiol* **65**: 1858-1863.
Mojzita D, Herold S, Metz B, Seiboth B & Richard P (2012) L-xylo-3-Hexulose Reductase Is the Missing Link in the Oxidoreductive Pathway for D-Galactose Catabolism in Filamentous Fungi. *Journal of Biological Chemistry* **287**: 26010-26018.
Seiboth B & Metz B (2011) Fungal arabinan and L-arabinose metabolism. *Appl Microbiol Biot* **89**: 1665-1673.
Spickett CM, Smirnoff N & Pitt AR (2000) The biosynthesis of erythroascorbate in *Saccharomyces cerevisiae* and its role as an antioxidant. *Free Radical Bio Med* **28**: 183-192.
Tye R & Willetts A (1977) Fungal Growth on C1 Compounds - Quantitative Aspects of Growth of a Methanol-Utilizing Strain of *Trichoderma-Lignorum* in Batch Culture. *Appl Environ Microbiol* **33**: 758-761.
Yurimoto H, Lee B, Yano T, Sakai Y & Kato N (2003) Physiological role of S-formylglutathione hydrolase in C-1 metabolism of the methylotrophic yeast *Candida boidinii*. *Microbiol-Sgm* **149**: 1971-1979.
