# Supplementary material for: Evolution and comparative genomics of the most common Trichoderma species
Source: BMC Genomics. 2019 Jun 12;20:485. doi: 10.1186/s12864-019-5680-7 (PMC6560777; doi:10.1186/s12864-019-5680-7)
Supplement: Supplementary file 14 — Polysaccharide decomposition by Trichoderma. (PDF 201 kb) [file 12864_2019_5680_MOESM14_ESM.pdf]

Polysaccharide decomposition by *Trichoderma*

Contents

Polysaccharide decomposition by *Trichoderma* 1
Plant cell wall degradation 1
Fungal cell wall polysaccharide degradation 3
Degradation of α-glucans 3
Degradation of uronic acid polymers 4
References 4

Plant cell wall degradation

The glycosyl hydrolases involved in plant polysaccharide degradation – such as cellulases, xylanases, and pectinases - and some of the associated accessorial enzymes (e.g. copper-dependent cellulose monooxygenases) and their evolution has recently been described for nine *Trichoderma* spp. in detail (Druzhinina *et al.*, 2018), and these data will therefore not be repeated here.

Concerning the accessory enzymes (Levasseur *et al.*, 2013), we identified 62 – 87 to be (some putatively though) involved plant polysaccharide degradation. Among them, three auxiliary enzymes of family AA9 (representing copper-dependent lytic polysaccharide monooxygenases, (LPMOs (Ferreira *et al.*, 2015)), have already been described in detail in *T. reesei* (Kubicek & Kubicek, 2016) and orthologs are present in all other *Trichoderma* species. Of the remaining 12 families so far incepted, eight are also present in all *Trichoderma* spp. Families AA2 and AA4, which comprise lignin peroxidases and vanillin alcohol oxidases, are absent from *Trichoderma* which is consistent with the fact that *Trichoderma* is unable to degrade lignin. However, *Trichoderma* spp. contain AA7 glucooligosaccharide oxidases, which underlines the importance of oxidative glucose catabolism as reported above, and also AA8 iron reductases. The latter consist of a cytochrome domain (protoheme IX) of spectral class b, and have implicated into cellulose degradation by Fenton chemistry (Eastwood *et al.*, 2011). This mechanism has so far been described only for brown rot fungi (Cragg *et al.*, 2015), but data suggesting the occurrence of this mechanism also in *T. reesei* and *N. crassa* have recently been presented (Schmoll *et al.*, 2012, Bischof *et al.*, 2013).

With regards to the deacetylation of hemicelluloses, all *Trichoderma* spp. contain members of the CE5 family which comprises - although not exclusively - acetylxylan esterases. Interestingly, CE8 pectin methylesterases are absent from species of section Longibrachiatum but all *Trichoderma* spp. contain a single copy of the CE15 4-O-methyl-glucuronyl methylesterase.

Numbers of carbohydrate esterases, auxiliary activities and polysaccharide lyases in *Trichoderma*

Enzyme type	Family	Enzyme	*Trichoderma* spp. *											
			R	L	C	P	H	G	F	V	A	M	S	U
Carbhydrate esterases	CE1	acetyl xylan esterase, feruloyl esterase and others	5	5	5	5	7	7	7	7	6	5	6	6
	CE1/2		1				1			2	2	2	2	2
	CE1/3		1				1			2	2	2	2	2
Auxiliary activities	AA3	GMC oxidoreductases					1	1						
	AA3/2	glucose- and aryl-alcohol oxidase	9	9	9	9	16	16	15	14	9	9	9	9
	AA3/3	alcohol oxidase	2	2	2	2	2	2	2	2	2	2	2	2
	AA5/1	glyoxal oxidase								1	1	1	1	1
	AA6	1,4-benzoquinone reductase	1	1	1	1	1	1	1	1	1	1	1	1
	AA7	gluco- or chitooligosaccharide oxidase	1	1	1	1	1	1	1	2	1	1	1	1
	AA8	iron reductase	1	1	1	1	3	2	2	6	5	3	4	3
	AA9	lytic cellulose monooxygenase	3	3	3	3	3	3	3	3	2	2	2	2
	AA11	lytic chitin monooxygenase	3	3	3	3	3	3	3	3	2	2	2	2
	AA12	pyrroloquinoline quinone-dependent oxidoreductase	1	1	1	1	1	1		1	1		1	
	PL1/4	pectate lyase									1	1		1
	PL20	endo-β-1,4-glucuronan lyase	2	2	2	2	2	2	2	2	2	2	2	2
Polysaccharide lyases	PL7/4	ß-mannuronate and alginate lyase	2	2	2	2	4	4	4	3	3	3	3	3
	PL8	hyaluronate lyase	1	1	1	1	1	1	1	1	1	1	1	1
Total			33	31	31	31	47	44	41	50	41	37	39	38

Trichoderma one-letter code: P, T. parareesei; R, T. reesei, L, T. longibrachiatum, C, T. citrinoviride, V, T. virens, H, T. harzianum; F. T. afroharzianum, G, T. guizhouense; A, T. atroviride; M, T. gamsii; S, T. asperellum; U, T. hamatum

Carbohydrate binding modules (CBM) are generally believed to function by concentrating the respective partner enzyme at their dedicated substrates and so enhance the catalytic efficacy, what is particularly important for the degradation of refractory substrates (Boraston *et al.*, 2004). However, although CBMs have been defined as modules within the primary structures of carbohydrate-active enzymes, this paradigm is now diluted by several findings of independent CBM proteins (Charnock *et al.*, 2002, Flint *et al.*, 2004, Vaaje-Kolstad *et al.*, 2005, Abbott *et al.*, 2008, Abramyan & Stajich, 2012). The CAZyme database currently hosts 81 CBM families, of which 12 are found in *Trichoderma* (Figure 8A). Four of them (CBM1, CBM13, CBM43 and CBM66) are found as domains in cellulolytic and hemicellulolytic enzymes. CBM1 is present in most of the cellulases, several hemicellulases, but also some of the acetyl esterases, one of the AA9 enzymes, the expansin-like protein swollenin and the protein CIP1 (whose function in cellulose depolymerisation has not yet been revealed), illustrating its importance for efficient cellulose breakdown. In addition, CBM42 domains, reported to bind to α-L-arabinofuranoside residues, are present in the GH54 α-L-arabinofuranosidases (Ribeiro *et al.*, 2010).

The CBM66 domain has been shown in bacteria to bind to nonreducing terminal fructose residues in fructans (Cuskin *et al.*, 2012), but in *Trichoderma* it is associated with GH43 α-L-arabinofuranosidases. This link between arabinan and fructan degradation is difficult to explain because these two polymers do not occur in close vicinity or association. However, the CBM66 domain has high similarity to the laminin G domains, which serve as receptors for various structures including α-dystroglycans (Givant-Horwitz *et al.*, 2005). We therefore consider it likely that the CBM66 domains in fungal α-L-arabinofuranosidases bind to a different structure than fructosides. Two independent CBM66 proteins (one only in species of section *Longibrachiatum*) are also present in the *Trichoderma* genomes, but both lack a signal peptide. Their function is therefore intracellular.

Finally, *Trichoderma* contains 3-4 CBM13 proteins that are supposed to bind xylan (Leskinen *et al.*, 2005). In *Aspergillus* spp. CBM13 domains occur in α–D-galactosidases, but the *Trichoderma* CBM13 proteins occur as individual proteins. Like the CBM66 proteins, they also lack a signal peptide and thus cannot act outside of the cells, and their true function needs further investigation.

Fungal cell wall polysaccharide degradation

Fungal cell walls are made up by proteins and polysaccharides, chitin and ß-1,3/1,6-glucans thereby comprising the highest amount. The cell wall is subject to turnover during growth and development, and fungi consequently possess a variety of chitinases and ß-glucanases. In addition, *Trichoderma* as a mycoparasite needs these enzymes for digestion of host cell walls too. It is therefore not surprising that chitinases and ß-glucanases, as already reported earlier [12, 16] represent the CAZymes that are most abundant in *Trichoderma* (Figure 7).

Intriguingly, the GH18-B and GH18-C chitinases (which are distinguished by the presence of a CBM1 cellulose-binding module and of CBM18/CBM50 (=LysM) chitin binding modules, respectively; (Hartl *et al.*, 2012)) show a strong difference between the three *Trichoderma* sections and clades (Figure 8). The combination of one or more CBM18 and CBM50 is unique to species of HV, and may serve to increase the overall affinity to the polymer (Abramyan & Stajich, 2012). The addition of a CBM1 module to chitinases of subgroup 18-B has been shown to enhance binding and chitinase activity on insoluble chitin, which is probably due to the fact that three aromatic amino acids (W, F and Y) of CBM1, known to be required for binding to cellulose, are also required for binding to chitin (Limon *et al.*, 2001).

Thirteen CBM50 domains – but none of the CBM18 domains - also occur as independent proteins, consist of up to 5 tandem copies and were most abundant in HV. Expression of these independent CBM modules has been shown in the case of CBM50 and *T. atroviride* (Gruber *et al.*, 2011).

2-4 genes encoding CE4 chitin deacetylases were also found (see above), of which two contain 2 and 3, respectively, CBM18 chitin binding modules that flank the catalytic domain. Acetyl esterases containing CBM18 modules have so far only been reported for the amphibian pathogen *Batrachochytrium dendrobatidis* (Liu & Stajich, 2015), but a BLASTP analysis shows that they are present in several other Sordariomycetes too. They deacetylate chitin to chitosan, which can lead to the formation of a chitosan layer on top of chitin (Cord-Landwehr *et al.*, 2016), thus protecting the cell wall against either its own chitinases and those from competing or host organisms, and prevent the recognition of the fungus by plants. In another fungus, *Pochonia chlamydospora*, chitosanases were demonstrated to be important for its nematophagous activity (Aranda-Martinez *et al.*, 2016).

Finally, all *Trichoderma* spp. possess 3-4 genes encoding proteins of family AA11 that comprises lytic chitin monooxygenase, thereby completing the tools for chitin degradation. None of them however contains an X278 domain, as described for the enzyme from *A. oryzae* (Hemsworth *et al.*, 2014), or another chitin binding domain (CBM18, CBM50).

Degradation of α-glucans

In contrast to the degradation of plant cell wall polysaccharides, the ability of *Trichoderma* to degrade starch and other α-linked polysaccharides has not been systematically studied yet. Starch is the major energy storage of plants, and is composed of two distinct glucose polymers: amylose, comprising essentially unbranched α-(1→4)-linked glucose residues, and the larger and branched amylopectin, produced by the formation of α-(1→6) linkages

between adjoining straight glucan chains on an α-(1→4) backbone (Miao *et al.*, 2015). Despite the simplicity of this chemical structure, amylopectin and amylose molecules are organized radially in a supramolecular assembly thereby forming water insoluble granules that vary in size and crystallinity (Buleon *et al.*, 1998). This renders them poorly accessible to the active sites of amylolytic enzymes. To degrade starch, the GH inventory of *Trichoderma* displays several GH13 (subfamily 1 and 40, respectively) α-amylases and GH31 α-glucosidases, two GH15 glucoamylases and a single GH133 amylo-α-1,6-glucosidase, of which 3, 2, 4 and 1, respectively, occur in the *Trichoderma* core genome. Two of the GH15 and one of the GH13, all belong to the core genes, contain carbohydrate binding domains (see below).

In addition, a single GH4 α-glucosidase occurs in species of the *T. harzianum* complex. GH4 has so far been found only in bacterial and archaeal taxa (Hall *et al.*, 2009), but a BLASTP search revealed that an orthologue occurs also in *Talaromyces stipitatus* and *Aspergillus sydowii* with higher similarity and lower E-value than from anaerobic bacteria of the phylum *Chloroflexi*, which are frequently found in anaerobic environments containing plant biomass (Tian *et al.*, 2017). It is therefore thinkable that some Eurotiales obtained this gene from bacteria by HGT, from which is was subsequently laterally transferred to *Trichoderma*. Despite of the fact that the closest neighbours have been annotated as α–galactosidases, a function of the fungal GH4 enzyme is difficult to predict because this GH family contains α-glycosidases and 6-phospho-α-and -β-glycosidases (Hall *et al.*, 2009). However, a function in the degradation of phosphorylated glycosides is less likely because the GH4 protein contains a signal peptide and is therefore secreted.

Three of the starch degrading enzymes also contained CBMs, i.e. CBM48 (that binds amorphous starch like glycogen (Janecek *et al.*, 2011)) which is present in one GH13, and a granular starch binding CBM20 (Nekiunaite *et al.*, 2016) that occurs in both GH15 glucoamylases. This illustrates the importance of glucamylase in the attack of the initially recalcitrant granular starch. Another starch-binding CBM - CBM21 (Chou *et al.*, 2006) - occurred only as individual protein.

Interestingly, members of the recently discovered AA13 family that encodes a lytic starch monooxygenase (Vu *et al.*, 2014), and which are present in most Sordario- and Eurotiomycetes (data not shown), were not found in *Trichoderma*.

Degradation of uronic acid polymers

Cleavage of glycosidic bonds by ß-elimination is an important mechanism in the decomposition of uronic acid polymers and has been well demonstrated for pectin (Sutherland, 1995). It is performed by polysaccharide lyases (PL). The presence of pectate lyases in *Trichoderma* has recently been described (Druzhinina *et al.*, 2018). However, *Trichoderma* also possesses PL7 alginate lyases, PL8 hyaluronate lyases, and a PL20 endo-ß-1,4-glucuronan lyase (se above). Alginates are components of the in the cell walls of brown algae, and present in bacterial biofilms, whereas hyaluronic acid is a key component of the envelope of epithelial cells in mammals. The true substrate specificity of any of these enzymes has not yet been investigated but their presence suggests that *Trichoderma* may have the capability of degrading the envelope of algal and mammalian cells.

References

Abbott DW, Eirin-Lopez JM & Boraston AB (2008) Insight into ligand diversity and novel biological roles for family 32 carbohydrate-binding modules. *Mol Biol Evol* **25**: 155-167.

Abramyan J & Stajich JE (2012) Species-Specific Chitin-Binding Module 18 Expansion in the Amphibian Pathogen *Batrachochytrium dendrobatidis*. *Mbio* **3**.
Aranda-Martinez A, Lenfant N, Escudero N, Zavala-Gonzalez EA, Henrissat B & Lopez-Llorca LV (2016) CAZyme content of *Pochonia chlamydosporia* reflects that chitin and chitosan modification are involved in nematode parasitism. *Environ Microbiol* **18**: 4200-4215.
Bischof R, Fourtis L, Limbeck A, Gamauf C, Seiboth B & Kubicek CP (2013) Comparative analysis of the *Trichoderma reesei* transcriptome during growth on the cellulase inducing substrates wheat straw and lactose. *Biotechnol Biofuels* **6**: 127.
Boraston AB, Bolam DN, Gilbert HJ & Davies GJ (2004) Carbohydrate-binding modules: fine-tuning polysaccharide recognition. *Biochem J* **382**: 769-781.
Buleon A, Colonna P, Planchot V & Ball S (1998) Starch granules: structure and biosynthesis. *Int J Biol Macromol* **23**: 85-112.
Charnock SJ, Bolam DN, Nurizzo D, Szabo L, McKie VA, Gilbert HJ & Davies GJ (2002) Promiscuity in ligand-binding: The three-dimensional structure of a *Piromyces* carbohydrate-binding module, CBM29-2, in complex with cello- and mannohexaose. *Proc Natl Acad Sci U S A* **99**: 14077-14082.
Chou WI, Pai TW, Liu SH, Hsiung BK & Chang MDT (2006) The family 21 carbohydrate-binding module of glucoamylase from Rhizopus oryzae consists of two sites playing distinct roles in ligand binding. *Biochemical Journal* **396**: 469-477.
Cord-Landwehr S, Melcher RL, Kolkenbrock S & Moerschbacher BM (2016) A chitin deacetylase from the endophytic fungus *Pestalotiopsis sp.* efficiently inactivates the elicitor activity of chitin oligomers in rice cells. *Sci Rep* **6**: 38018.
Cragg SM, Beckham GT, Bruce NC*, et al.* (2015) Lignocellulose degradation mechanisms across the Tree of Life. *Curr Opin Chem Biol* **29**: 108-119.
Cuskin F, Flint JE, Gloster TM*, et al.* (2012) How nature can exploit nonspecific catalytic and carbohydrate binding modules to create enzymatic specificity. *Proc Natl Acad Sci USA* **109**: 20889-20894.
Druzhinina IS, Chenthamara K, Zhang J*, et al.* (2018) Massive lateral transfer of genes encoding plant cell wall-degrading enzymes to the mycoparasitic fungus *Trichoderma* from its plant-associated hosts. *PLOS Genetics* **14**: e1007322.
Eastwood DC, Floudas D, Binder M*, et al.* (2011) The plant cell wall-decomposing machinery underlies the functional diversity of forest fungi. *Science* **333**: 762-765.
Ferreira P, Carro J, Serrano A & Martinez AT (2015) A survey of genes encoding H2O2-producing GMC oxidoreductases in 10 Polyporales genomes. *Mycologia* **107**: 1105-1119.
Flint J, Nurizzo D, Harding SE, Longman E, Davies GJ, Gilbert HJ & Bolam DN (2004) Ligand-mediated dimerization of a carbohydrate-binding molecule reveals a novel mechanism for protein-carbohydrate recognition. *J Mol Biol* **337**: 417-426.
Givant-Horwitz V, Davidson B & Reich R (2005) Laminin-induced signaling in tumor cells. *Cancer Lett* **223**: 1-10.
Gruber S, Vaaje-Kolstad G, Matarese F, Lopez-Mondejar R, Kubicek CP & Seidl-Seiboth V (2011) Analysis of subgroup C of fungal chitinases containing chitin-binding and LysM modules in the mycoparasite *Trichoderma atroviride*. *Glycobiology* **21**: 122-133.

Hall BG, Pikis A & Thompson J (2009) Evolution and Biochemistry of Family 4 Glycosidases: Implications for Assigning Enzyme Function in Sequence Annotations. *Mol Biol Evol* **26**: 2487-2497.
Hartl L, Zach S & Seidl-Seiboth V (2012) Fungal chitinases: diversity, mechanistic properties and biotechnological potential. *Appl Microbiol Biot* **93**: 533-543.
Hemsworth GR, Henrissat B, Davies GJ & Walton PH (2014) Discovery and characterization of a new family of lytic polysaccharide monooxygenases. *Nat Chem Biol* **10**: 122-126.
Janecek S, Svensson B & MacGregor EA (2011) Structural and evolutionary aspects of two families of non-catalytic domains present in starch and glycogen binding proteins from microbes, plants and animals. *Enzyme Microb Tech* **49**: 429-440.
Kubicek CP & Kubicek EM (2016) Enzymatic deconstruction of plant biomass by fungal enzymes. *Curr Opin Chem Biol* **35**: 51-57.
Leskinen S, Mantyla A, Fagerstrom R, Vehmaanpera J, Lantto R, Paloheimo M & Suominen P (2005) Thermostable xylanases, Xyn10A and Xyn11A, from the actinomycete *Nonomuraea flexuosa*: isolation of the genes and characterization of recombinant Xyn11A polypeptides produced in *Trichoderma reesei*. *Appl Microbiol Biot* **67**: 495-505.
Levasseur A, Drula E, Lombard V, Coutinho PM & Henrissat B (2013) Expansion of the enzymatic repertoire of the CAZy database to integrate auxiliary redox enzymes. *Biotechnology for Biofuels* **6**.
Limon MC, Margolles-Clark E, Benitez T & Penttila M (2001) Addition of substrate-binding domains increases substrate-binding capacity and specific activity of a chitinase from *Trichoderma harzianum*. *FEMS Microbiol Lett* **198**: 57-63.
Liu P & Stajich JE (2015) Characterization of the Carbohydrate Binding Module 18 gene family in the amphibian pathogen *Batrachochytrium dendrobatidis*. *Fungal Genet Biol* **77**: 31-39.
Miao M, Jiang B, Cui SW, Zhang T & Jin Z (2015) Slowly Digestible Starch—A Review. *Critical Reviews in Food Science and Nutrition* **55**: 1642-1657.
Nekiunaite L, Arntzen MO, Svensson B, Vaaje-Kolstad G & Abou Hachem M (2016) Lytic polysaccharide monooxygenases and other oxidative enzymes are abundantly secreted by *Aspergillus nidulans* grown on different starches. *Biotechnology for Biofuels* **9**.
Ribeiro T, Santos-Silva T, Alves VD, Dias FMV, Luis AS, Prates JAM, Ferreira LMA, Romao MJ & Fontes CMGA (2010) Family 42 carbohydrate-binding modules display multiple arabinoxylan-binding interfaces presenting different ligand affinities. *Bba-Proteins Proteom* **1804**: 2054-2062.
Schmoll M, Tian C, Sun J, Tisch D & Glass NL (2012) Unravelling the molecular basis for light modulated cellulase gene expression - the role of photoreceptors in *Neurospora crassa*. *Bmc Genomics* **13**: 127.
Sutherland IW (1995) Polysaccharide Lyases. *FEMS Microbiol Rev* **16**: 323-347.
Tian T, Tam NFY, Zan Q, Cheung SG, Shin PKS, Wong YS, Zhang L & Chen Z (2017) Performance and bacterial community structure of a 10-years old constructed mangrove wetland. *Mar Pollut Bull* **124**: 1096-1105.

Vaaje-Kolstad G, Horn SJ, van Aalten DMF, Synstad B & Eijsink VGH (2005) The non-catalytic chitin-binding protein CBP21 from *Serratia marcescens* is essential for chitin degradation. *Journal of Biological Chemistry* **280**: 28492-28497.
Vu VV, Beeson WT, Span EA, Farquhar ER & Marletta MA (2014) A family of starch-active polysaccharide monooxygenases. *Proc Natl Acad Sci USA* **111**: 13822-13827.
