# Supplementary material for: Evolution and comparative genomics of the most common Trichoderma species
Source: BMC Genomics. 2019 Jun 12;20:485. doi: 10.1186/s12864-019-5680-7 (PMC6560777; doi:10.1186/s12864-019-5680-7)
Supplement: Supplementary file 16 — Secondary metabolism genes of Trichoderma. (PDF 318 kb) [file 12864_2019_5680_MOESM16_ESM.pdf]

Secondary metabolism-related genes of *Trichoderma*

Contents

Polyketide synthases 1
Non-ribosomal peptide synthases (NRPS) 1
Terpenoids 3
Absence of alkaloid biosynthesis 3
References 4

Polyketide synthases

The twelve *Trichoderma* genomes harbour 39 PKS-synthases, of which 9 – 20 genes occur in individual species: species from SL have the lowest, whereas species from HV contain the highest number of PKS-encoding genes (**Additional File 16**). Thirteen, 13 and 10 PKSs were thereby present in the all species of HV, TS and SL, respectively, but only five of them present in the core genome. With the exception of PKS4, which is involved in conidial pigmentation, defence and stress resistance (Atanasova *et al.*, 2013) – none of them have been functionally characterized. Interestingly, four of them are otherwise only known from distantly related fungi such as Eurotio- or Leotiomycetes, thus suggesting possibilities for the massive gene loss or lateral gene transfer that occurred during the evolution of these genes. Further five genes are specifically only found in species of HV and ST, and two PKSs (SOR1 and SOR2, catalysing the biosynthesis of sorbicillinoids; (Druzhinina *et al.*, 2016)) are present only in species of SL. The remaining PKSs are dispersed among the 12 species in a clade/section-specific manner.

Non-ribosomal peptide synthases (NRPS)

A phylogenetic analysis of the twelve *Trichoderma* genomes revealed between 11 (*T. longibrachiatum*) and 29 (*T. virens* and *T. asperellum*) NRPS-encoding genes (**Additional File 16**). Fifteen, 12 and 11 of them occurred in all species of the HV clade and the LB and ST sections, respectively. Six of them occurred in all three sections (see below and **Additional File 16**).

With respect to the NRPS present in the *Trichoderma* core genome, two of them encoded a large and a small peptaibol (peptides containing α-aminoisobutyric acid and a C-terminal 1,2-amino alcohol) synthase (Duclohier, 2007)), respectively, which are unique for the mycoparasitic species of the Hypocreales; (Quandt *et al.*, 2015)). It indicates that these large proteins evolved at least 170 Mya. The other 7 NRPSs have next neighbors in all Hypocreales, and therefore most likely evolved by vertical transfer.

The core genome also contained two siderophore synthases (a SID3-like ferricrocin synthase and an NPS6-like siderophore synthase; cf. (Haas, 2014)). Iron acquisition is an important component of microbial competition, especially in habitats with high populations of different

microbes (Kosman, 2003). The intracellular siderophore ferricrocin is responsible for storage of iron (Wallner *et al.*, 2009), whereas the extracellular siderophore produced by NPS6 is also a virulence factor in some plant pathogens (Oide *et al.*, 2006). The competition for iron could also be a mechanism contributing to *Trichoderma* mycoparasitism, as it was shown to be important for control of *Fusarium* wilt of tomato by *T. asperellum* (Segarra *et al.*, 2010). Both siderophore synthesizing NRPSs are also known to protect fungi from oxidative stress (Brandon *et al.*, 2015, Li *et al.*, 2016).

Further, the core genome contained an NRPS with high similarity to sirodesmin synthase from *Leptosphaeria maculans* (Gardiner *et al.*, 2004), and four unknown NRPS (Additional File 16). Sirodesmin belongs to the epipolythiodioxopiperazine (ETP) class of toxins produced by fungi including mammalian and plant pathogens (Fox & Howlett, 2008). However, the *Trichoderma* sirodesmin synthase is – unlike in other fungi - not located in a gene cluster (Patron *et al.*, 2007), and it can therefore not be deduced that the product of this synthase is indeed similar to sirodesmin

However, we detected an NRPS synthesizing the ETP gliotoxin that was originally found only in *T. reesei* and *T. virens* (Patron *et al.*, 2007). It is present in all species of SL and HV, and the arrangement of the biosynthetic cluster is also conserved (although some rearrangement occurred in *T. afroharzianum* and *T. virens* (Figure 1).

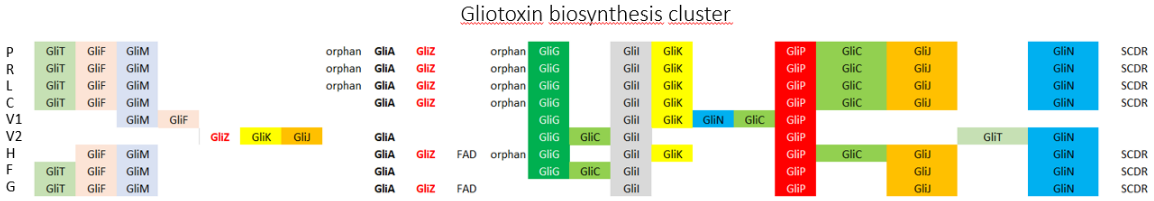

Figure 1. Architecture of the putative gliotoxin biosynthesis cluster in species of SL and HV. Trichoderma *species are abbreviated by a single letter: P,* T. parareesei*; R,* T. reesei*, L,* T. longibrachiatum*, C,* T. citrinoviride*, V,* T. virens*, H,* T. harzianum*; F.* T. afroharzianum*, G,* T. guizhouense*. Occurrence of genes in the clusters is shown from 5' to 3', with the exception of* T. reesei *and* T. afroharzianum *where the order is 3' to 5'. Abbreviations (GliC and GliF cytochrome P450 monooxygenases; GliG, glutathione S-transferase; GliI, aminotransferase; GliJ, depeptidase; GliK, γ-glutamyl-cyclotransferase; GliM, O-methyltransferase; GliZ, Zn2Cys6 transcriptional regulator; GliA, MSF (in* T. virens *ABC-) transporter; GliN, methyltransferase; GliP, NRPS). The clusters are located on the following scaffolds, and the described genes can be retrieved from there:* T. reesei *1:2720000-2770000;* T. parareesei *LFMI010000476:12100-51100;* T. longibrachiatum*, 9:102000-137000,* T. citrinoviride *18:198000-245000;* T. harzianum*, 8:11000-38000;* T. guizhouense*, LVVK01000003:556794-605014;* T. afroharzianum *JOKZ01000238.1:8850-48000;* T. virens *Gv 29-8 (V1), complete scaffold 47; T. virens G20-4VIB (V2), EF429246. Other abbreviations: FAD, FAD-dependent monooxygenase; SCDR, short chain dehydrogenase/reductase.*

Four consecutively located genes are missing in *T. virens* Gv29-8, which is most likely due to the fact that the *T. virens* cluster is located on a very short scaffold (scaffold 47) whose 3' end is in the middle of the gliotoxin cluster. Although the missing genes were not found elsewhere in the *T. virens* genome, we are convinced that they are present because the isolate Gv29-8 that was used for genome sequencing produces gliotoxin (Djonovic *et al.*, 2006). Interestingly, the cluster of isolate G20-4VIB (V2 in Figure 1), obtained by cosmid cloning (Patron *et al.*, 2007), showed a strong re-arrangement and also missed some genes. This suggests that the gliotoxin biosynthetic cluster of *T. virens* may undergo gene shuffling. The presence of this cluster in two sections of *Trichoderma* that evolved in parallel about 50 Mya makes it unlikely that it does not serve a function. Gliotoxin belongs also to the ETP class of peptides, and was originally found in *T. virens* where it was shown to be fungistatic (Djonovic *et al.*, 2006), and is also known as avirulence factor of the opportunistic human pathogen *Aspergillus fumigatus* (Eurotiales) where it suppresses macrophage immune function (Schlam *et al.*, 2016).

Terpenoids

Terpenoid secondary metabolites are synthesized from five-carbon isopentenyl units (Bian *et al.*, 2017). The twelve *Trichoderma* spp. revealed an arsenal between 6 (*T. hamatum, T. reesei* and *T. longibrachiatum*) and 12 (*T. virens*) terpenoid synthase genes. In contrast to the numbers of NRPSs and PKSs, which are smallest in species from SL, the genomes of *T. citrinoviride* and *T. parareesei* contained 8 and 9 terpenoid synthases, respectively, the numbers equalling that in some species of HV (*T. harzianum, T. guizhouense, T. afroharzianum*). The terpenoid synthases formed 18 clusters, three of which (a lanosterol synthase, a pentalene synthase and a presilphi-perfolan-8-ß-ol synthase) were shared by all species and thus part of the core genome. Of these, the pentalene synthase has next neighbors only in the Eurotiomycetes and may therefore also have been derived by lateral gene transfer. The other two core genes were present in Hypocreales and thus likely derived by vertical transfer. Among the remaining 15 clusters, six were shared between species from SL and HV, and one cluster was shared HV and ST. ST contained three, and SL only a single section-specific cluster.

Two terpenoid synthases, including the well characterized sesquiterpene synthase VIR4 (Trivi2:56195; (Crutcher *et al.*, 2013), and a kaurene synthase from *T. asperellum* were only found in a single species (Figure 2).

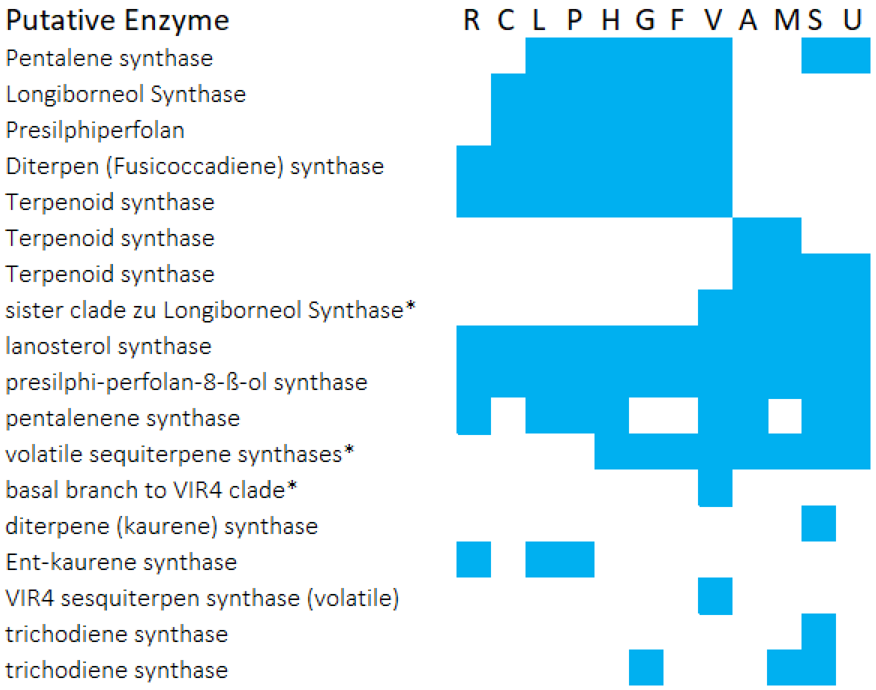

Figure 2 Presence of terpenoid synthases in different Trichoderma clades and species (for one-letter code see Figure 1; A, T. atroviride; M, T. gamsii; S, T. asperellum; U, T. hamatum). The yellow boxed terpenoid synthases are considered to be part of the core genome. For accession numbers see Additional File 16

T. asperellum and *T. guizhouense* also contained two and one gene, respectively, encoding a trichothecene synthase (Additional file 16). Biosynthesis of trichothecenes by *Trichoderma* has been documented for *T. brevicompactum* and *T. arundinaceum*, and these two species also contained the genes encoding the respective processing enzymes in clusters that resembled those in *Fusarium* spp. (Cardoza *et al.*, 2011). However a BLASTP in *T. asperellum* and *T. guizhouense* did not detect the presence of these processing genes, neither in close vicinity to

the synthase gene, nor elsewhere in the genome, and we therefore assume that the trichothecene synthase genes present in these two species may either be evolutionary relicts or serve other purposes.

Absence of alkaloid biosynthesis

In contrast to the above described synthases, *Trichoderma* seems not to synthesize alkaloids because we could not find the precursor dimethylallyl tryptophan synthases (DMATS; (Yu & Li, 2012)) within any genome of *Trichoderma* spp., neither by BLASTP using the respective enzymes from *Claviceps purpurea, Metarhizium robertsii* (Hypocreales) or *Aspergillus fumigatus*, nor by searching for the InterPro numbers (IPR017795 (Aro_prenylTrfase_DMATS) or IPR012148 (fungal type DMATS).

References

Atanasova L, Knox BP, Kubicek CP, Druzhinina IS & Baker SE (2013) The Polyketide Synthase Gene pks4 of *Trichoderma reesei* Provides Pigmentation and Stress Resistance. *Eukaryotic Cell* **12**: 1499-1508.

Bian GK, Deng ZX & Liu TG (2017) Strategies for terpenoid overproduction and new terpenoid discovery. *Curr Opin Biotech* **48**: 234-241.

Brandon M, Howard B, Lawrence C & Laubenbacher R (2015) Iron acquisition and oxidative stress response in aspergillus fumigatus. *Bmc Syst Biol* **9**.

Cardoza RE, Malmierca MG, Hermosa MR, Alexander NJ, McCormick SP, Proctor RH, Tijerino AM, Rumbero A, Monte E & Gutierrez S (2011) Identification of Loci and Functional Characterization of Trichothecene Biosynthesis Genes in Filamentous Fungi of the Genus *Trichoderma*. *Appl Environ Microbiol* **77**: 4867-4877.

Crutcher FK, Parich A, Schuhmacher R, Mukherjee PK, Zeilinger S & Kenerley CM (2013) A putative terpene cyclase, vir4, is responsible for the biosynthesis of volatile terpene compounds in the biocontrol fungus *Trichoderma virens*. *Fungal Genetics and Biology* **56**: 67-77.

Djonovic S, Pozo MJ, Dangott LJ, Howell CR & Kenerley CM (2006) Sm1, a proteinaceous elicitor secreted by the biocontrol fungus *Trichoderma* virens induces plant defense responses and systemic resistance. *Mol Plant Microbe In* **19**: 838-853.

Druzhinina IS, Kubicek EM & Kubicek CP (2016) Several steps of lateral gene transfer followed by events of 'birth-and-death' evolution shaped a fungal sorbicillinoid biosynthetic gene cluster. *BMC Evol Biol* **16**: 269.

Duclohier H (2007) Peptaibiotics and Peptaibols: An Alternative to Classical Antibiotics? *Chemistry & Biodiversity* **4**: 1023-1026.

Fox EM & Howlett BJ (2008) Biosynthetic gene clusters for epipolythiodioxopiperazines in filamentous fungi. *Mycological Research* **112**: 162-169.

Gardiner DM, Cozijnsen AJ, Wilson LM, Pedras MSC & Howlett BJ (2004) The sirodesmin biosynthetic gene cluster of the plant pathogenic fungus *Leptosphaeria maculans*. *Mol Microbiol* **53**: 1307-1318.

Haas H (2014) Fungal siderophore metabolism with a focus on *Aspergillus fumigatus*. *Nat Prod Rep* **31**: 1266-1276.

Kosman DJ (2003) Molecular mechanisms of iron uptake in fungi. *Mol Microbiol* **47**: 1185-1197.

Li Y, Wang ZK, Liu X, Song ZY, Li R, Shao CW & Yin YP (2016) Siderophore Biosynthesis but Not Reductive Iron Assimilation Is Essential for the Dimorphic Fungus *Nomuraea rileyi* Conidiation, Dimorphism Transition, Resistance to Oxidative Stress, Pigmented Microsclerotium Formation, and Virulence. *Front Microbiol* **7**.

Oide S, Moeder W, Krasnoff S, Gibson D, Haas H, Yoshioka K & Turgeon BG (2006) NPS6, Encoding a Nonribosomal Peptide Synthetase Involved in Siderophore-Mediated Iron Metabolism, Is a Conserved Virulence Determinant of Plant Pathogenic Ascomycetes. *The Plant Cell* **18**: 2836-2853.

Patron NJ, Waller RF, Cozijnsen AJ, Straney DC, Gardiner DM, Nierman WC & Howlett BJ (2007) Origin and distribution of epipolythiodioxopiperazine (ETP) gene clusters in filamentous ascomycetes. *BMC Evol Biol* **7**.

Quandt CA, Bushley KE & Spatafora JW (2015) The genome of the truffle-parasite *Tolypocladium ophioglossoides* and the evolution of antifungal peptaibiotics. *Bmc Genomics* **16**.

Schlam D, Canton J, Carreno M, Kopinski H, Freeman SA, Grinstein S & Fairn GD (2016) Gliotoxin Suppresses Macrophage Immune Function by Subverting Phosphatidylinositol 3,4,5-Trisphosphate Homeostasis. *Mbio* **7**.

Segarra G, Casanova E, Aviles M & Trillas I (2010) *Trichoderma asperellum* Strain T34 Controls Fusarium Wilt Disease in Tomato Plants in Soilless Culture Through Competition for Iron. *Microb Ecol* **59**: 141-149.

Wallner A, Blatzer M, Schrettl M, Sarg B, Lindner H & Haas H (2009) Ferricrocin, a Siderophore Involved in Intra- and Transcellular Iron Distribution in *Aspergillus fumigatus*. *Appl Environ Microbiol* **75**: 4194-4196.

Yu X & Li SM (2012) Prenyltransferases of the Dimethylallyltryptophan Synthase Superfamily. *Method Enzymol* **516**: 259-278.
