# Supplementary material for: Evolution and comparative genomics of the most common Trichoderma species
Source: BMC Genomics. 2019 Jun 12;20:485. doi: 10.1186/s12864-019-5680-7 (PMC6560777; doi:10.1186/s12864-019-5680-7)
Supplement: Supplementary file 18 — Small cysteine-rich secreted effector proteins in Trichoderma. (PDF 210 kb) [file 12864_2019_5680_MOESM18_ESM.pdf]

Small cysteine-rich secreted effector proteins

Contents

Small cysteine-rich secreted effector proteins 1
The list of SSCPs in *Trichoderma* (SP – signal peptide) 2
References 21

To identify SSCPs, we screened the genomes of the 12 *Trichoderma* spp. for proteins that contained a signal peptide, were less than 300 amino acids long and in which cysteine residues made up for at least 5 % of all amino acids. The latter value was chosen from the fact that the proteome of the 12 species exhibited an average cysteine content of 1.43 ± 0.37 %, and we considered a roughly threefold increase over this value content as “cysteine rich”. The number of so detected SSCPs is shown below and Table 7, with *T. asperellum* containing 125 and *T. parareesei* only containing 27. It is also striking that – in contrast to other protein groups - their number showed no clade specific pattern, and the variation was species-specific. This is also corroborated by the finding of only 21 SSCPs in the core genome. Irrespective of this, the number of SSCPs in *Trichoderma* is considerably smaller than that found in other fungal genomes (Stergiopoulos & de Wit, 2009).

Hydrophobins are small (6-8 kDa), secreted proteins that are characterized by a specific arrangement of 8 cysteine residues (C1 – C8), and which possess the ability to alter the hydrophobicity/hydrophily of surfaces. Dependent on the spacing of C4 and C5 and their solubility, they are usually grouped into two classes (Wosten, 2001, Linder *et al.*, 2005). *Trichoderma* is very rich in hydrophobins, ranging from 7 in species from section *Longibrachiatum* to 16 in *T. atroviride* of section *Trichoderma*. Thirteen of them can be categorized as class II hydrophobins (Kubicek *et al.*, 2008), of which 6 are present in the core genome (see Additional File 9). They include orthologues of *T. reesei* HFB1 - HFB5 (Kubicek *et al.*, 2008), and a larger (>12 kDa) hydrophobin that has so far not been detected. Interestingly, the latter contains an N-terminal, cysteine-rich domain and a C-terminal domain consisting to 50 % of A, P, S and T, which flank the hydrophobin domain which – untypically for class II hydrophobins – contains a very short (6 aa) helix. This hydrophobin is strongly conserved also within most Sordariomycetes, suggesting that it may serve an important yet unknown function.

Four class II HFBs are specifically found only in species from section *Trichoderma*, and three exclusively in species of HV. In addition, the sections *Longibrachiatum* and HV share the presence of a class II HFB with a long, P/G-rich N-terminus which has in *T. reesei* been named HFB6 (Kubicek *et al.*, 2008).

Trichoderma does not contain class I hydrophobins. Instead, species of HV and ST contain three hydrophobins that belong to a recently defined subclass with properties of both classes I and II (Seidl-Seiboth *et al.*, 2011). In the latter paper, two such genes were also reported to occur in *T. longibrachiatum*, but we could not find any of them in its genome. The sequences used in (Seidl-Seiboth *et al.*, 2011) were obtained by translated of ESTs from a *bone-fide T. longibrachiatum* strain (Vizcaino *et al.*, 2007). We have therefore analysed

several other ESTs of this strain by blastx, and the best hits were always obtained with *T. virens* instead of species from the SL. We therefore believe that the EST used in the above paper is in fact not from *T. longibrachiatum,* and this new subclass is in view of our results therefore absent from SL.

Cerato-platanin proteins are also fungal-specific, small and secreted proteins but contain only four conserved cysteines, and are believed to be important for interaction with other organisms (Gaderer *et al.*, 2014) and eliciting defense reactions in plants (Djonovic *et al.*, 2006, Gaderer *et al.*, 2015). *Trichoderma* contains three of these proteins (EPL1, EPL2, EPL3), for which orthologs are present in each species.

The list of SSCPs in *Trichoderma* (SP – signal peptide)

Taxon	Accession number	SSCPs	SP length	score
T. afroharzianum		66		
	KKO96486		15	0.834
	KKO96498		19	0.811
	KKO96855		21	0.847
	KKO96914		19	0.894
	KKO97159		23	0.698
	KKO97203		18	0.907
	KKO97206		16	0.875
	KKO98188		19	0.525
	KKO98216		19	0.869
	KKO98316		18	0.751
	KKO98661		23	0.784
	KKO98965		20	0.811
	KKO98980		16	0.868
	KKO98988		20	0.849
	KKO99034		18	0.829
	KKO99268		18	0.707
	KKO99269		19	0.889
	KKO99411		16	0.818
	KKO99621		24	0.681
	KKO99642		16	0.837
	KKO99647		20	0.743
	KKO99806		16	0.729
	KKP00012		20	0.776
	KKP00107		18	0.86
	KKP00119		20	0.798
	KKP00132		20	0.804
	KKP00290		21	0.526
	KKP00321		20	0.942
	KKP00430		16	0.702
	KKP00438		20	0.719
	KKP00601		19	0.596

	KKP00624		18	0.842
	KKP00947		21	0.706
	KKP01427		16	0.786
	KKP01472		18	0.713
	KKP02296		18	0.748
	KKP02655		20	0.883
	KKP02884		21	0.84
	KKP03014		17	0.784
	KKP03205		19	0.728
	KKP03432		15	0.851
	KKP03437		23	0.82
	KKP03524		18	0.84
	KKP03527		20	0.626
	KKP03550		24	0.699
	KKP03759		17	0.785
	KKP03914		16	0.808
	KKP04275		18	0.812
	KKP04312		20	0.858
	KKP04674		16	0.709
	KKP04739		20	0.947
	KKP04958		17	0.542
	KKP05074		20	0.767
	KKP05132		19	0.84
	KKP05145		17	0.706
	KKP05664		20	0.683
	KKP05851		19	0.82
	KKP06130		19	0.766
	KKP06303		16	0.642
	KKP06401		16	0.824
	KKP06584		19	0.812
	KKP06880		17	0.889
	KKP06986		20	0.867
	KKP07389		26	0.689
	KKP07456		16	0.834
	KKP07499		22	0.718
T. atroviride		75		
	314699		16	0.847
	28723		18	0.842
	28931		22	0.720
	30929		17	0.765
	32713		21	0.890
	32903		19	0.761
	35461		20	0.948

	40838		21	0.827
	42766		15	0.730
	45244		18	0.742
	45489		16	0.884
	46849		18	0.781
	48461		19	0.753
	48890		20	0.709
	50405		17	0.885
	55116		16	0.847
	77418		18	0.682
	77489		16	0.806
	82345		15	0.871
	84221		19	0.518
	84995		18	0.818
	85197		23	0.548
	85327		16	0.478
	86583		19	0.824
	87502		20	0.818
	89743		19	0.821
	89816		20	0.784
	90157		19	0.688
	90673		18	0.861
	91362		21	0.773
	94066		19	0.652
	94099		17	0.734
	138574		20	0.604
	146062		19	0.746
	171091		14	0.452
	212005		18	0.663
	214851		17	0.702
	219909		23	0.468
	224623		19	0.707
	231060		20	0.840
	254731		21	0.848
	255048		22	0.720
	256070		17	0.517
	256403		16	0.784
	256835		15	0.754
	258206		16	0.723
	258295		15	0.787
	259618		21	0.658
	259624		21	0.477
	271339		20	0.777

	281086		22	0.748
	297515		17	0.658
	297727		27	0.481
	297979		21	0.711
	298185		35	0.552
	298319		34	0.485
	299222		18	0.525
	299224		19	0.802
	299543		16	0.782
	300373		21	0.506
	300654		19	0.598
	300702		18	0.879
	300903		16	0.592
	301270		16	0.815
	302423		19	0.871
	302587		16	0.809
	302682		18	0.510
	303083		21	0.709
	304287		15	0.472
	305231		19	0.749
	306761		16	0.867
	310262		20	0.748
	310562		15	0.826
	322584		15	0.838
	323147		20	0.849
T. gamsii		42		
	XP_018657641		21	0.693
	XP_018663666		19	0.687
	XP_018663627		26	0.783
	XP_018663607		15	0.818
	XP_018656268		20	0.948
	XP_018660026		18	0.874
	XP_018661344		19	0.541
	XP_018657678		17	0.823
	XP_018657659		20	0.714
	XP_018662057		18	0.567
	XP_018666430		20	0.708
	XP_018666449		19	0.824
	XP_018666534		19	0.801
	XP_018661955		20	0.815
	XP_018661943		19	0.571
	XP_018664531		20	0.867
	XP_018656117		19	0.792

	XP_018656118		20	0.803
	XP_018662733		21	0.773
	XP_018665576		19	0.760
	XP_018660790		18	0.835
	XP_018662912		15	0.679
	XP_018666007		19	0.846
	XP_018665139		19	0.761
	XP_018665166		19	0.750
	XP_018658201		21	0.759
	XP_018658242		16	0.914
	XP_018655748		17	0.848
	XP_018658076		21	0.813
	XP_018663406		17	0.633
	XP_018663333		18	0.655
	XP_018665857		16	0.868
	XP_018664132		17	0.874
	XP_018664130		17	0.862
	XP_018665054		16	0.763
	XP_018665041		23	0.756
	XP_018662424		20	0.784
	XP_018663141		17	0.721
	XP_018658788		17	0.785
	XP_018661493		15	0.783
	XP_018657555		23	0.659
	XP_018657347		19	0.610
	XP_018658877		19	0.726
	XP_018658836		19	0.868
	XP_018658393		15	0.826
	XP_018659872		21	0.806
	XP_018659426		16	0.811
	XP_018665698		19	0.821
	XP_018665700		18	0.886
	XP_018666205		15	0.675
	XP_018657023		20	0.769
	XP_018655736		21	0.701
T. guizhouense		44		
	OPB44820		17	0.889
	OPB36613		20	0.942
	OPB37186		16	0.913
	OPB37502		18	0.876
	OPB37525		21	0.738
	OPB37603		16	0.839
	OPB37634		18	0.663
	OPB38530		16	0.773
	OPB38637		18	0.843

	OPB38772		19	0.859
	OPB38899		18	0.748
	OPB38900		18	0.839
	OPB39042		19	0.836
	OPB39052		19	0.596
	OPB39529		19	0.847
	OPB40230		20	0.878
	OPB40410		16	0.835
	OPB40452		22	0.723
	OPB40515		18	0.793
	OPB40661		16	0.852
	OPB40679		19	0.827
	OPB40918		17	0.784
	OPB41939		19	0.812
	OPB42521		20	0.792
	OPB42647		17	0.720
	OPB43857		15	0.813
	OPB43872		20	0.680
	OPB44008		19	0.822
	OPB44528		18	0.780
	OPB44600		16	0.812
	OPB44696		16	0.867
	OPB46132		16	0.890
	OPB46673		23	0.686
	OPB41390		17	0.557
	OPB41244		23	0.726
	OPB44951		15	0.861
	OPB39663		18	0.748
	OPB43682		20	0.819
	OPB37929		18	0.795
	OPB41745		24	0.708
	OPB36274		20	0.847
	OPB36111		16	0.702
	OPB36040		20	0.705
	OPB35941		19	0.681
T. harzianum		113		
	132934		18	0.867
	195		20	0.840
	247		18	0.700
	1760		19	0.596
	1820		20	0.775
	3176		21	0.736
	5619		20	0.729
	7726		15	0.918

	10061		21	0.793
	10849		19	0.889
	10918		16	0.784
	11493		20	0.838
	14808		16	0.777
	19060		17	0.466
	72403		18	0.852
	74377		21	0.743
	76763		16	0.817
	79424		16	0.913
	79555		18	0.871
	82972		20	0.950
	83008		19	0.847
	86900		16	0.858
	87195		23	0.638
	89031		19	0.772
	89146		16	0.855
	89933		20	0.865
	93744		18	0.783
	94876		16	0.832
	97657		17	0.770
	98114		18	0.783
	98997		19	0.800
	99275		16	0.782
	102121		22	0.641
	134143		19	0.801
	142486		20	0.680
	144956		16	0.832
	147959		16	0.847
	152397		23	0.637
	157431		19	0.850
	170055		16	0.702
	198819		31	0.784
	212147		17	0.537
	231529		25	0.550
	234055		19	0.507
	234588		17	0.450
	244374		19	0.757
	244717		21	0.579
	314749		17	0.527
	327585		21	0.753
	372941		23	0.656
	373844		27	0.511

	389440		17	0.485
	394493		28	0.486
	415456		20	0.682
	416142		27	0.702
	426066		19	0.830
	433808		21	0.524
	443708		18	0.525
	451526		21	0.595
	476937		16	0.816
	478239		24	0.644
	479033		20	0.700
	480242		23	0.558
	481706		28	0.609
	485265		20	0.613
	488463		20	0.842
	489366		16	0.852
	492015		17	0.827
	492105		17	0.844
	493416		17	0.907
	495727		17	0.776
	496184		20	0.838
	496231		23	0.831
	496268		23	0.689
	497684		19	0.748
	498376		14	0.703
	499123		16	0.868
	501444		18	0.687
	502416		19	0.850
	502599		17	0.557
	502685		16	0.700
	503468		16	0.834
	503784		32	0.750
	504228		24	0.580
	504679		19	0.833
	505024		24	0.626
	505052		17	0.919
	505375		21	0.782
	505665		24	0.479
	505679		20	0.777
	505981		19	0.857
	506080		24	0.736
	507026		22	0.661
	508128		21	0.925

	508663		18	0.823
	508974		19	0.714
	509631		21	0.668
	510010		19	0.560
	510508		20	0.776
	511478		17	0.890
	512173		18	0.746
	512550		19	0.601
	512586		24	0.691
	514362		18	0.786
	519288		30	0.490
	519857		19	0.710
	523481		18	0.669
	525011		17	0.718
	530682		15	0.813
	533861		18	0.758
	535541		18	0.784
	539293		31	0.526
	544145		24	0.908
T. longibrachiatum		89		
	1402025		16	0.885
	8542		16	0.830
	38581		11	0.639
	75082		26	0.534
	80155		46	0.535
	91205		17	0.722
	96360		23	0.665
	154710		19	0.643
	158578		22	0.630
	172014		21	0.568
	201078		26	0.538
	223269		29	0.574
	238281		23	0.517
	244812		25	0.482
	300564		25	0.609
	329082		21	0.652
	334975		18	0.562
	341074		33	0.549
	346097		17	0.858
	361622		26	0.527
	1006893		18	0.818
	1028342		19	0.669
	1047641		16	0.833

	1058805		17	0.565
	1062690		26	0.683
	1063970		21	0.656
	1070379		24	0.546
	1086601		28	0.601
	1110868		19	0.524
	1136070		21	0.529
	1150927		19	0.889
	1154811		22	0.636
	1183905		26	0.698
	1187726		18	0.641
	1191645		23	0.628
	1266014		21	0.509
	1321471		17	0.786
	1328082		16	0.613
	1334050		16	0.794
	1340025		18	0.753
	1341182		17	0.841
	1342360		17	0.864
	1347716		16	0.799
	1356080		16	0.786
	1357105		19	0.746
	1373787		16	0.740
	1378939		19	0.742
	1392226		25	0.499
	1393193		16	0.903
	1394941		18	0.633
	1396680		22	0.675
	1397045		19	0.835
	1397521		15	0.854
	1397785		23	0.774
	1397796		20	0.726
	1398368		19	0.522
	1399398		19	0.705
	1399466		28	0.606
	1399735		17	0.803
	1399879		16	0.795
	1400006		23	0.639
	1400891		21	0.875
	1400902		26	0.781
	1401130		22	0.886
	1401407		16	0.867
	1401525		18	0.955

	1401645		17	0.827
	1401876		21	0.641
	1402256		19	0.476
	1402359		24	0.712
	1402572		20	0.717
	1404410		18	0.697
	1404415		18	0.723
	1404451		19	0.789
	1404595		19	0.712
	1404658		19	0.664
	1405283		21	0.918
	1405395		20	0.815
	1405397		29	0.581
	1405473		30	0.645
	1405997		30	0.484
	1421968		19	0.885
	1424428		21	0.705
	1426882		19	0.763
	1437539		17	0.827
	1439862		16	0.881
	1448530		25	0.665
	1460034		19	0.799
T. reesei		39		
	109911		14	0.805
	50323		16	0.778
	54407		24	0.704
	66077		20	0.836
	70840		19	0.671
	73173		15	0.691
	79448		18	0.602
	103135		18	0.804
	103174		19	0.702
	103393		17	0.657
	104293		15	0.605
	104401		16	0.768
	105311		18	0.803
	105533		16	0.711
	106453		16	0.712
	106538		23	0.571
	106615		15	0.582
	106662		22	0.592
	107131		19	0.944
	107347		21	0.869

	108663		21	0.594
	108684		18	0.609
	109231		19	0.648
	109253		19	0.693
	109255		18	0.730
	111205		16	0.718
	111495		15	0.707
	111499		18	0.748
	111915		16	0.876
	112037		15	0.785
	119989		14	0.754
	120479		17	0.953
	120697		17	0.786
	121135		14	0.748
	121177		18	0.835
	121739		18	0.743
	123236		20	0.745
	123967		15	0.786
	124295		18	0.910
T. virens		65		
	91716		22	0.704
	19266		21	0.874
	19757		16	0.771
	27800		19	0.862
	29260		16	0.829
	31923		18	0.737
	32462		19	0.688
	32688		22	0.872
	38080		23	0.679
	48810		17	0.850
	49849		16	0.824
	51487		11	0.513
	53684		16	0.756
	55099		20	0.701
	57903		24	0.682
	58093		19	0.586
	58578		16	0.798
	58944		23	0.634
	60531		16	0.748
	61653		19	0.779
	62229		25	0.603
	65112		18	0.782
	66299		16	0.851

	66518		23	0.796
	68245		16	0.679
	68434		19	0.846
	69117		20	0.783
	70311		20	0.737
	72996		16	0.844
	79197		19	0.894
	80895		24	0.706
	83985		16	0.703
	84522		26	0.675
	87351		19	0.594
	91466		16	0.836
	92071		18	0.900
	92434		18	0.818
	92793		17	0.886
	93159		16	0.886
	110875		16	0.871
	111642		19	0.882
	111693		19	0.703
	111979		17	0.716
	121648		18	0.709
	139640		16	0.527
	140013		18	0.669
	143417		18	0.747
	151784		17	0.721
	153055		17	0.764
	181575		18	0.772
	199191		20	0.664
	203083		20	0.776
	214571		19	0.880
	215664		21	0.497
	216814		17	0.666
	216987		19	0.830
	218116		17	0.824
	220441		19	0.806
	220864		20	0.947
	222643		17	0.628
	222876		18	0.887
	223574		17	0.887
	223913		15	0.840
	224489		17	0.675
	225030		22	0.898
T. parareesei		29		

	OTA06008		17	0.836
	OSZ99916		15	0.854
	OTA00002		21	0.753
	OTA00133		19	0.719
	OTA00252		19	0.773
	OTA00542		17	0.770
	OTA00644		17	0.899
	OTA00659		16	0.875
	OTA01064		19	0.731
	OTA01214		20	0.945
	OTA02273		19	0.695
	OTA02329		19	0.802
	OTA02386		23	0.616
	OTA02552		16	0.891
	OTA02752		16	0.807
	OTA03335		19	0.679
	OTA03470		17	0.841
	OTA03699		16	0.836
	OTA04052		17	0.714
	OTA04103		18	0.744
	OTA04385		19	0.848
	OTA04651		16	0.720
	OTA04742		18	0.819
	OTA05122		21	0.882
	OTA05223		19	0.837
	OTA05364		17	0.789
	OTA05368		26	0.675
	OTA05568		16	0.836
	OTA07131		17	0.785
T. citrinoviride		50		
	1100855		16	0.815
	5227		19	0.874
	5235		16	0.675
	8349		18	0.700
	16079		16	0.811
	17627		19	0.931
	17796		19	0.655
	18747		19	0.739
	46602		16	0.921
	88347		16	0.508
	147689		19	0.806
	169621		22	0.612
	1086954		22	0.681
	1092251		13	0.626

	1101056		11	0.639
	1102229		20	0.768
	1107525		15	0.837
	1109864		16	0.746
	1111500		16	0.792
	1111637		17	0.827
	1112845		20	0.718
	1114798		18	0.720
	1119292		19	0.779
	1125125		18	0.757
	1127293		19	0.750
	1129790		18	0.718
	1130164		22	0.601
	1136340		30	0.637
	1138585		16	0.837
	1138588		20	0.788
	1139264		20	0.949
	1144258		20	0.754
	1144537		21	0.876
	1155954		23	0.884
	1159901		21	0.898
	1160463		16	0.792
	1160941		22	0.836
	1162118		15	0.899
	1162782		21	0.920
	1163232		16	0.892
	1163406		19	0.775
	1164830		19	0.849
	1165528		23	0.516
	1169208		16	0.848
	1169425		19	0.875
	1169823		19	0.826
	1171563		17	0.897
	1177777		16	0.876
	1198765		15	0.814
	1199549		18	0.955
T. asperellum		125		
	59495		12	0.490
	22516		18	0.715
	27615		20	0.724
	28394		20	0.779
	31204		19	0.624
	31301		18	0.746

	31425		15	0.890
	32032		23	0.718
	32222		17	0.701
	32345		16	0.805
	32550		19	0.746
	36514		16	0.755
	37831		17	0.734
	39409		17	0.746
	39645		16	0.792
	42434		19	0.837
	45639		19	0.588
	49234		30	0.873
	49818		18	0.643
	52328		19	0.827
	53400		23	0.764
	53970		19	0.772
	54018		18	0.795
	54114		27	0.666
	54127		19	0.814
	54184		16	0.726
	54328		15	0.826
	54798		16	0.767
	55019		24	0.668
	55026		19	0.659
	56705		20	0.771
	57108		15	0.757
	57227		15	0.808
	57390		19	0.883
	57392		18	0.596
	57894		16	0.761
	58285		16	0.516
	59378		19	0.583
	59451		16	0.894
	59966		18	0.889
	60347		19	0.816
	60885		16	0.774
	61323		16	0.875
	61427		18	0.759
	62096		20	0.755
	62649		17	0.851
	62892		17	0.684
	62906		24	0.734
	63139		22	0.924

	63211		18	0.766
	67114		22	0.720
	68465		22	0.715
	68659		21	0.749
	69080		20	0.862
	70064		19	0.787
	72036		22	0.570
	76519		25	0.584
	77473		20	0.948
	79195		19	0.855
	79480		21	0.520
	80972		16	0.871
	83007		19	0.735
	83987		18	0.840
	85019		19	0.717
	85087		15	0.698
	90491		15	0.690
	127130		19	0.481
	127371		22	0.758
	134808		23	0.491
	137174		17	0.838
	137492		16	0.919
	140010		21	0.610
	141005		19	0.802
	144631		18	0.777
	153750		23	0.475
	154964		20	0.795
	160296		22	0.860
	167230		20	0.919
	169160		16	0.726
	178063		19	0.817
	189193		21	0.766
	192721		16	0.620
	194232		18	0.879
	215139		18	0.827
	219551		27	0.515
	223108		20	0.485
	223341		20	0.546
	225444		17	0.610
	230258		17	0.665
	245387		20	0.673
	248206		20	0.760
	254425		16	0.454

	255498		18	0.957
	260136		24	0.530
	262100		15	0.698
	269305		20	0.468
	272418		17	0.518
	280186		26	0.576
	300403		22	0.532
	302363		19	0.805
	314258		22	0.679
	315796		30	0.794
	316126		26	0.592
	320233		16	0.650
	334543		17	0.811
	338113		16	0.621
	372405		25	0.666
	383665		30	0.509
	392456		18	0.554
	396221		36	0.576
	398992		18	0.616
	400716		30	0.596
	403403		16	0.683
	408531		35	0.491
	408961		20	0.522
	413218		16	0.669
	415421		20	0.851
	416739		32	0.656
	421281		23	0.626
	425737		28	0.573
	426769		17	0.698
	431832		18	0.648
	433671		15	0.615
	441255		19	0.548
	445739		20	0.793
T. hamatum		62		
	35		24	0.833
	635		16	0.779
	791		26	0.774
	813		16	0.802
	834		19	0.742
	989		16	0.696
	1031		15	0.891
	1419		18	0.656
	1508		18	0.809

	1698		21	0.614
	1905		15	0.854
	2191		22	0.828
	2619		20	0.882
	2807		16	0.743
	3196		17	0.611
	3293		19	0.755
	3374		21	0.852
	3890		16	0.708
	3953		17	0.702
	5251		19	0.806
	5324		17	0.669
	5515		17	0.640
	5724		17	0.773
	5959		15	0.895
	6082		17	0.892
	6163		16	0.885
	6389		18	0.807
	6600		15	0.692
	6613		16	0.895
	6629		18	0.634
	6718		18	0.671
	6926		16	0.914
	7100		20	0.763
	7336		17	0.811
	7432		17	0.768
	7665		20	0.851
	7782		19	0.503
	7788		24	0.668
	8228		20	0.948
	8489		23	0.760
	8598		17	0.671
	8703		16	0.677
	8815		17	0.618
	8822		16	0.826
	9237		16	0.790
	9238		18	0.584
	9380		17	0.757
	9451		19	0.759
	9566		20	0.805
	9721		21	0.707
	9748		18	0.647
	9950		20	0.947

	10003		16	0.783
	10072		19	0.912
	10211		17	0.728
	10454		21	0.782
	10462		15	0.904
	10494		18	0.777
	10534		17	0.632
	10667		21	0.785
	10669		18	0.766
	10727		20	0.822

References

Djonovic S, Pozo MJ, Dangott LJ, Howell CR & Kenerley CM (2006) Sm1, a proteinaceous elicitor secreted by the biocontrol fungus *Trichoderma virens* induces plant defense responses and systemic resistance. *Mol Plant Microbe In* **19**: 838-853.

Gaderer R, Bonazza K & Seidl-Seiboth V (2014) Cerato-platanins: a fungal protein family with intriguing properties and application potential. *Appl Microbiol Biot* **98**: 4795-4803.

Gaderer R, Lamdan NL, Frischmann A, Sulyok M, Krska R, Horwitz BA & Seidl-Seiboth V (2015) Sm2, a paralog of the *Trichoderma* cerato-platanin elicitor Sm1, is also highly important for plant protection conferred by the fungal-root interaction of Trichoderma with maize. *BMC Microbiol* **15**.

Kubicek CP, Baker S, Gamauf C, Kenerley CM & Druzhinina IS (2008) Purifying selection and birth-and-death evolution in the class II hydrophobin gene families of the ascomycete *Trichoderma/Hypocrea*. *BMC Evol Biol* **8**.

Linder MB, Szilvay GR, Nakari-Setala T & Penttila ME (2005) Hydrophobins: the protein-amphiphiles of filamentous fungi. *FEMS Microbiol Rev* **29**: 877-896.

Seidl-Seiboth V, Gruber S, Sezerman U, Schwecke T, Albayrak A, Neuhof T, von Dohren H, Baker SE & Kubicek CP (2011) Novel Hydrophobins from *Trichoderma* Define a New Hydrophobin Subclass: Protein Properties, Evolution, Regulation and Processing. *J Mol Evol* **72**: 339-351.

Stergiopoulos I & de Wit PJGM (2009) Fungal Effector Proteins. *Annu Rev Phytopathol* **47**: 233-263.

Vizcaino JA, Redondo J, Suarez MB, Cardoza RE, Hermosa R, Gonzalez FJ, Rey M & Monte E (2007) Generation, annotation, and analysis of ESTs from four different *Trichoderma* strains grown under conditions related to biocontrol. *Appl Microbiol Biot* **75**: 853-862.

Wosten HAB (2001) Hydrophobins: Multipurpose proteins. *Annu Rev Microbiol* **55**: 625-646.
